# Cancer-associated fibroblasts (CAFs) derived from MFAP2 promote CRC proliferation and metastasis while suppressing CD8^+^ T cell-mediated antitumor immunity

**DOI:** 10.1038/s41419-026-08413-w

**Published:** 2026-01-30

**Authors:** Xu Zhang, Yuxiang Fei, Chunqi Xie, Tao Li, Ben Niu, Zihao Yang, Mengwei Song, Fanjun Meng, Hongting Diao, Jing Ji, Qianming Du, Chao Liu

**Affiliations:** 1 Department of Pharmacy, Chengdu Integrated TCM & Western Medicine Hospital, Chengdu University of TCM, 18# Wanxiang East Road, Chengdu, 610041 P.R. China; 2 Department of Pharmacy, Nanjing First Hospital, Nanjing Medical University, Nanjing, 210006 P.R. China; 3 Jiangsu Key Laboratory of Marine Pharmaceutical Compound Screening, College of Pharmacy, Jiangsu Ocean University, Lianyungang, 222001 P.R. China; 4 General Clinical Research Center, Nanjing First Hospital, Nanjing Medical University, Nanjing, 210006 P.R. China; 5 School of Basic Medicine and Clinical Pharmacy, China Pharmaceutical University, Nanjing, 211198 P.R. China; 6 School of Nursing, Nanjing Medical University, Nanjing, 211168 P.R. China; 7 College of Pharmacy, Taizhou University, Taizhou, 225300 P.R. China

**Keywords:** Cancer microenvironment, Drug development, Colon cancer, Immune cell death, Cancer microenvironment

## Abstract

Colorectal cancer (CRC) ranks among the most prevalent malignancies of the digestive system, with the intricate tumor immune microenvironment (TIME) emerging as a key determinant of poor prognosis. Cancer-associated fibroblasts (CAFs), a central constituent of the tumor microenvironment, critically influence tumorigenesis and progression by orchestrating immunosuppression through cytokine secretion and other mechanisms. This study investigates the multifaceted interplay between CAFs and the immune system to identify novel therapeutic targets and improve prognostic outcomes for CRC patients. Through comprehensive analyses of clinical samples and public database data, we identified elevated MFAP2 expression in both CRC tissues and fibroblasts. Mechanistically, we established that CAFs-derived MFAP2 interacts with integrin β8 (ITGB8) on cancer cell surfaces, activating the integrin-FAK-ERK1/2 signaling cascade to drive CRC progression. Furthermore, ERK1/2 phosphorylates and activates the transcription factor ETS2, which upregulates the expression of CYP27A1, an enzyme that modulates lipid metabolism and suppresses CD8^+^ T cell function via liver X receptor beta (LXRβ) signaling. These findings elucidate a novel MFAP2-ITGB8-FAK-ERK1/2-ETS2-CYP27A1-LXRβ signaling axis, significantly activated by CAFs-derived MFAP2 in both in vitro and in vivo models, contributing to immune exhaustion and tumor progression. This axis offers significant therapeutic and prognostic potential for CRC, providing critical insights into CAF-mediated immune modulation and paving the way for targeted immunotherapeutic strategies.

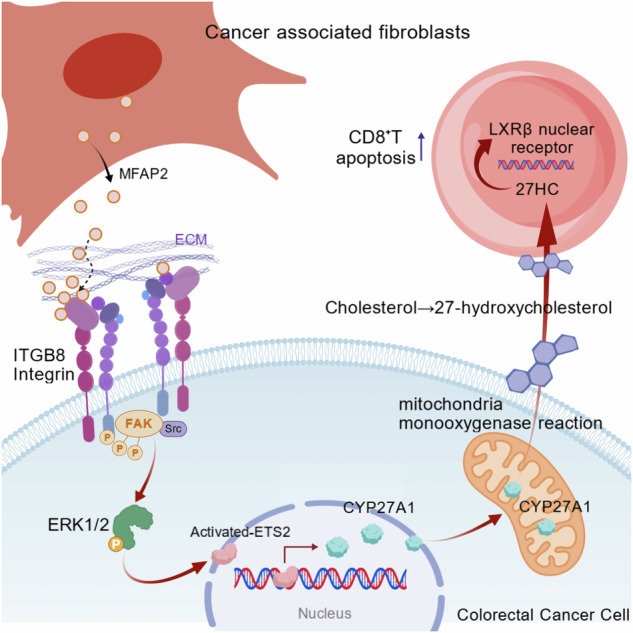

## Introduction

Colorectal cancer (CRC) is the third most commonly diagnosed cancer worldwide and the second leading cause of cancer-related mortality [1]. The development of CRC is primarily associated with lifestyle factors such as smoking, unhealthy diet, excessive alcohol consumption, obesity, and physical inactivity [2]. Data suggest that by 2040, the global incidence of CRC will increase by 3.2 million new cases, and mortality will rise by 1.6 million deaths, significantly contributing to the growing healthcare burden worldwide [3].

Despite significant advancements in surgical treatment, immunotherapy, radiation therapy, and pharmacotherapy, the improvement in the 5-year relative survival rate for CRC patients has been limited in recent decades [4]. Approximately 20% of CRC patients present with distant metastasis at the time of diagnosis, with the liver being the most common site of metastatic spread [5, 6]. Curative surgical resection and chemotherapy are the standard treatment approaches for patients with CRC liver metastasis (CRLM) [7]. However, due to factors such as tumor location and size, presence of other comorbidities, and inoperability, surgical treatment is only applicable in 10–20% of cases, with a 5-year survival rate as low as 30% [8, 9].

Therefore, there is an urgent clinical need for effective biomarkers and novel therapeutic targets to improve the diagnosis and treatment of CRC and CRLM. A deeper understanding of the molecular mechanisms underlying the progression of CRC and liver metastasis will contribute to enhancing the success rate of CRC treatments and improving overall patient survival.

Cancer is not merely a genetic disease, but a complex ecosystem in which progression is not an isolated event, but involves multiple genetic mutations and alterations in cellular proliferation [10]. The tumor microenvironment (TME) includes surrounding cells such as epithelial cells, cancer-associated fibroblasts (CAFs), endothelial cells (ECs), pericytes, and immune cells. These cells secrete various types of cytokines, which play a crucial role in the mechanisms of cancer initiation and metastasis [11]. The cellular signaling regulation mediated by the crosstalk between tumor cells and their microenvironment further promotes cancer progression [12].

CAFs are key components of TME, contributing to connective tissue proliferation, immune modulation, and the secretion of growth factors that can influence tumor progression. While CAFs often promote tumor growth, their role is complex, as certain subsets may also exert tumor-suppressive effects, highlighting the need for further investigation into their diverse functions. [13, 14]. CAFs are a heterogeneous cell population, including inflammatory CAFs (iCAFs) and myofibroblastic CAFs (myCAFs) [15]. These studies suggest that different CAF subtypes have distinct functions in the TME, and further research is needed to elucidate the crosstalk and regulatory mechanisms between tumor cells and CAFs [16, 17].

Numerous studies have shown that the number of tumor-infiltrating lymphocytes (TILs) in the TME, particularly CD8^+^ T cells, is highly correlated with the survival rate of patients with advanced CRC [18]. CD8^+^ T cells are powerful effector cells in anti-tumor immune responses, capable of specifically recognizing and killing cancer cells [19]. CD8^+^ T cells within tumors often exhibit dysfunction or exhaustion. Therefore, increasing CD8^+^ T cell infiltration and activity, as well as improving or even reversing CD8^+^ T cell exhaustion, has become a key strategy in immunotherapy for CRC.

Microfibril-associated protein 2 (MFAP2), also known as microfibril-associated glycoprotein 1 (MAGP1), is an essential component of extracellular elastic microfibrils [20]. MFAP2 consists of 183 amino acids and two structural domains: the N-terminal half, which is rich in proline and glutamine residues, and a 54-amino acid region at the C-terminal end [21]. MFAP2 interacts with extracellular proteins such as fibrillin, collagen VI, and biglycan. Intracellularly, it is involved in the regulation of downstream gene expression related to cell adhesion, motility, and matrix remodeling [22]. In recent years, the role of MFAP2 in cancer has gained increasing attention. For example, MFAP2 promotes gastric cancer proliferation through the integrin pathway, is associated with poor prognosis in hepatocellular carcinoma (HCC), and exacerbates ovarian cancer progression through the glycolytic process [23–25].

However, MFAP2 has mostly been studied as a component of the extracellular matrix (ECM) in the context of cancer progression, with limited research on its role in CRC. Therefore, we focused on elucidating the complex signaling pathways involving CAFs-derived MFAP2 and its relationship with CRC, as well as its effects on CD8^+^ T cells in the TME.

In this study, we identified elevated MFAP2 expression in both CRC tissues and fibroblasts. Mechanistically, we established that CAFs-derived MFAP2 interacts with integrin β8 (ITGB8) on cancer cell surfaces, activating the integrin–FAK–ERK1/2 signaling cascade to drive CRC progression. Furthermore, ERK1/2 phosphorylates and activates the transcription factor ETS2, which upregulates the expression of CYP27A1, an enzyme that modulates lipid metabolism and suppresses CD8^+^ T cell function via liver X receptor beta (LXRβ) signaling. These findings elucidate a novel MFAP2–ITGB8–FAK–ERK1/2–ETS2–CYP27A1–LXRβ signaling axis, significantly activated by CAFs-derived MFAP2 in both in vitro and in vivo models, contributing to immune exhaustion and tumor progression. This axis offers significant therapeutic and prognostic potential for CRC, providing critical insights into CAF-mediated immune modulation and paving the way for targeted immunotherapeutic strategies.

This study contributes to a deeper understanding of CAF-mediated immune suppression, provides valuable insights for the development of effective treatments for CRC and paves the way for clinical trials targeting the MFAP2–CYP27A1–LXRβ axis in CRC.

## Materials and methods

### Ethics statement

This study adhered to the principles outlined in the Declaration of Helsinki and received approval from the Ethics Committee of Nanjing First Hospital, Nanjing Medical University (Nanjing, China). The use of animals in the experiments was also approved by the Institutional Animal Care and Use Committee of Nanjing Medical University (Nanjing, China). Prior to conducting research using human samples, written informed consent was obtained from the patients who generously donated their samples. Furthermore, all human sample collections were conducted in accordance with the approval of the Institutional Research Ethics Committee.

In caring for the animals, strict compliance with the institutional guidelines of Nanjing First Hospital, Nanjing Medical University (Nanjing, China) was observed. The experimental protocol was approved by the Institutional Animal Care and Use Committee of Nanjing First Hospital, Nanjing Medical University (license number: SYXK (Su) 2021-0007). Throughout the study, the guidelines presented in the NIH guide for the Care and Use of Laboratory Animals (NIH Publications No. 80-23) were strictly followed. Meanwhile, all animal experiments were carried out in compliance with institutional ethical regulations and in strict accordance with the ARRIVE guidelines 2.0, ensuring both animal welfare and methodological transparency [26]. Every effort was made to minimize the number of mice used and to ensure their well-being and comfort. The assignments of animals to experimental groups were randomized to avoid any bias.

### Bioinformatics analysis

We used the GEPIA2 database to analyze the expression levels of MFAP2 in colon adenocarcinoma (COAD) samples, which included 349 healthy volunteers and 275 CRC patients. The analysis revealed that, when assessed based on the median gene expression level, MFAP2 expression was significantly higher in tumor samples compared to those from healthy volunteers.

Additionally, we divided the CRC samples into high and low MFAP2 expression groups based on the median gene expression levels. To assess the impact of MFAP2 expression on patient prognosis, we constructed Kaplan-Meier curves and calculated the Log-rank *P*-value to compare the overall survival rates between the two groups.

We further validated that fibroblasts are the primary source of MFAP2 using the tumor immune single-cell hub (TISCH) sequencing database (http://tisch.comp-genomics.org/). Similarly, in colon adenocarcinoma (COAD) samples from The Cancer Genome Atlas (TCGA) database, patients were stratified into high and low MFAP2 expression groups based on the median expression value. To explore the association between MFAP2 expression and the immune landscape of the TME, we conducted immune infiltration analysis using both CIBERSORT and TIMER algorithms. The CIBERSORT algorithm was applied to estimate the relative proportions of about 20 immune cell types within the TME. In addition, stromal score, immune score, and ESTIMATE score were calculated using the ESTIMATE algorithm to comprehensively evaluate the stromal and immune components in tumor tissues. These analyses provided insight into the relationship between MFAP2 expression and immune cell infiltration as well as the overall immune contexture in COAD. The sequencing data and R scripts used for the immune infiltration and microenvironment analyses are available from the corresponding author upon reasonable request.

We extracted and integrated single-cell RNA sequencing datasets from GSE225857, GSE178318, GSE221575, GSE110009, GSE235057, GSE166555, and GSE132465 with the aim of validating the cellular origin of MFAP2 at the single-cell level. This approach also assisted in identifying the predominant cell type or subtype responsible for the expression of our target gene of interest.

### CRC cell lines culture and reagent incubation

The SW620, SW480, HCT116, HT29, and MC38 cell lines were cultured in DMEM (ATCC, Rockville, MD, USA) supplemented with 10% fetal bovine serum (FBS, Hyclone, Logan, UT, USA) and penicillin-streptomycin solution (Thermo Fisher Scientific, Pittsburgh, PA, USA). The CT26 cell line was cultured in RPMI medium (Cytiva, Uppsala, Sweden) supplemented with 10% FBS and penicillin-streptomycin solution. Cells were maintained at 37 °C in a 5% CO_2_ incubator. All cell lines used in this study were obtained from the Cell Bank of the Chinese Academy of Sciences. The identity of each cell line was recently authenticated by short tandem repeat (STR) profiling, and all cell lines were routinely tested and confirmed to be free of mycoplasma contamination prior to experimentation.

The recombinant MFAP2 (rMFAP2, Thermo Fisher Scientific, Pittsburgh, PA, USA) was dissolved in DMEM containing 10% FBS to obtain a stock solution of 10 µg/mL (50×). SW620 and HT29 cells were incubated with rMFAP2 at a concentration of 200 ng/mL for 24 h, followed by functional assessment. For the incubation of small molecule inhibitors (FAK inhibitor, PF562271, Beyotime, Shanghai, China; Integrin inhibitor, Cilengitide TFA, Med Chem Express, Shanghai, China), the inhibitors were dissolved in culture medium containing DMSO at a final concentration not exceeding 0.1% for functional assessment.

### Isolation and culture of primary fibroblasts

Fresh tissue specimens from CRC patients, including both tumor and adjacent normal tissues, were obtained from the operating room and placed in DMEM complete medium containing 5% antibiotics at 4 °C. Subsequent CAFs extraction was performed in the laboratory. Vascular connective tissue and tumor necrotic areas were carefully removed from the tissue blocks. The tissue was then cut into 1 cm³ pieces. Using a 10 mL syringe, D-Hank’s solution was aspirated and gently flushed into the CRC tissue to wash the specimens slowly and thoroughly.

The flushing procedure was repeated until the wash solution became clear and colorless. The tissue pieces were then minced into smaller fragments and placed in the prepared digestion solution. The tissue was incubated at 37 °C on a shaking incubator set at 250 rpm for 1 h, with the centrifuge tube being gently inverted every 20 min. The digestion solution was transferred to a 100 µm cell strainer to remove the debris. The resulting digest filtrate was centrifuged at 1200 rpm for 5 min, followed by resuspension in PBS and three additional washings via centrifugation. CAFs derived from cancer tissues and normal fibroblasts (NFs) derived from adjacent normal tissues were purified through successive passages and can be cryopreserved. Cells up to the 10th passage were used for subsequent experiments.

To isolate mouse CAFs, CRC tissues were collected from BALB/c mice bearing CT26 tumor cells, following the same procedure described for the isolation of human-derived CAFs.

### shRNA/overexpression vector construction and transfection

shRNA sequences targeting MFAP2 (mouse and human), ITGB8 (mouse and human), ETS2 (mouse and human), and CYP27A1 (human) were cloned into the pLKO.1 vector, and the sequences are provided in Supplementary Table 1. pLKO.1 vectors, psPAX2 packaging plasmid and pMD2.G envelope plasmid were co-transfected into HEK293T cells using Lipofectamine 2000 (Invitrogen, USA). Viral supernatants were collected 48 h and 72 h after transfection, filtered through a 0.45 μm filter, and used to infect target cells (cancer cells, CAFs and CD8^+^ T cells as indicated in the result section) in the presence of 7.5 μg/mL polybrene. Infected cells were selected with puromycin (2 μg/mL) for 7–10 days to establish stable knockdown cell lines. Knockdown efficiency was confirmed by qPCR and Western blotting.

For ETS2 overexpression, specific primers were designed to amplify the full-length CDS of human or murine ETS2, incorporating BmtI (GCTAGC) and Acc65I (GGTACC) restriction sites to match the multiple cloning site of the expression vector. The amplified fragments were ligated into the pcDNA3.1-EGFP backbone (Thermo Fisher) to generate pcDNA3.1-EGFP-ETS2 for verification. The construct was further subcloned into a pLVX lentiviral vector, which was co-transfected with psPAX2 and pMD2.G plasmids into HEK293T cells for viral packaging. Viral supernatants were harvested, filtered, and concentrated before transduction into human or murine tumor cell lines. Stable ETS2-overexpressing cells were established by puromycin pressure selection and validated by EGFP fluorescence microscopy and Western blot.

### Establishment of orthotopic CRC model and the CRLM model

Male BALB/c mice (aged 6–8 weeks) and BALB/c nude mice (aged 6 weeks) were obtained from Charles River (Beijing, China) and Qizhen Laboratory Animal Technology Co., Ltd. (Hangzhou, China), respectively. Placing the mice in a light/dark cycle environment at 25 °C for one week to acclimate, while providing ad libitum access to food and water. Experimental animals were randomly assigned to groups using Microsoft Excel. Briefly, each animal was given a unique identification number, and the Excel RAND() function was applied to generate a random number for each ID. The list was then sorted according to the random numbers in ascending order. Animals were sequentially allocated into the designated groups based on this sorted order to ensure unbiased randomization.

During the establishment of the orthotopic CRC mouse model, mice were anesthetized with 2–3% isoflurane and placed on a heated surgical pad until they showed no response to tactile stimuli. In total, 5 × 10^5^ CAFs or shMFAP2-transfected CAFs were mixed with 5 × 10^5^ CT26-Luc cells suspended in 100 µL of 1640 medium (DMEM, Gibco, USA), and the cell mixture was injected into the cecal wall of BALB/c mice. Injections were performed under a microscope, with the needle tip positioned at an approximate 30 ° angle to the cecal wall, ensuring that the needle tip penetrated the cecal wall to a depth of 4 mm while avoiding bleeding or cellular reflux. Following the injection, the cecum was returned to the abdominal cavity, and the incision was sutured. Postoperative analgesia and antibiotics were administered to all mice. Four weeks after the injection, the mice were euthanized, and the tumor size was measured using calipers. Tumor volume was calculated using the formula: 1/2 × length × width².

A mouse model of CRLM was established, with preliminary procedures as described for the orthotopic model. A 1 cm incision was made in the skin and peritoneum just below the left rib cage using surgical scissors. The abdominal cavity was then accessed, and the spleen was exposed. Using curved forceps, the lower pole of the spleen was gently grasped and carefully elevated out of the abdominal cavity. A 31 G needle was inserted approximately 1.5 cm into the spleen, and 100 μL of cell suspension (1 × 10^6^ CT26 cells with or without an equal number of CAFs) was slowly injected. The injection site was gently pressed with a cotton swab for 2 min to ensure hemostasis. The spleen was then returned to the abdominal cavity, and the incision was sutured. Four weeks after the injection, the mice were euthanized, and the number of liver nodules was counted.

To establish the subcutaneous CRC model, CT26-Luc cells (1 × 10^6^) alone or mixed with an equal number of CAFs in 100 µL serum-free DMEM were injected subcutaneously into the right flank of BALB/c nude mice using a 27 G needle. Tumor growth was monitored every 2–3 days using calipers, and tumor volume was calculated as 1/2 × length × width². Mice were euthanized when tumors reached the endpoint criteria, and tumors were excised for further analysis.

rMFAP2 was dissolved in sterile PBS containing 1% BSA, and administered intraperitoneally at 0.1 mg/kg every other day (at a volume of 10 mL/kg). Administration was initiated when tumors became palpable. Vehicle controls received PBS containing 1% BSA on the same schedule.

### Establishment of co-culture system (CS)

Co-culture of two cell types was performed by seeding 1 × 10⁷ cells/mL of CAFs or shMFAP2-CAFs with 2 × 10⁶ cells/mL of SW620 or HT29 cells in a 24-well Transwell chamber. The co-culture was maintained for 10 days. SW620 or HT29 cells were implanted into the lower chamber, while CAFs or shMFAP2-CAFs were implanted into the upper chamber.

Isolation, activation, and expansion of human peripheral blood cytotoxic T cells (CD8^+^ T cells). Negative magnetic selection was performed using a CD8^+^ T cell isolation kit (Beyotime, Shanghai, China). CD8^+^ T cells were then activated for 5 days using antibodies against CD3 (2 μg/mL, Proteintech, USA) and CD28 (1 μg/mL, Proteintech, USA), along with IL-2 (MCE, 100 U/mL). After 48 h, cells were collected, centrifuged, and resuspended in RPMI complete medium supplemented with IL-7 (Immunotools, 25 ng/mL) at a concentration of 1 × 10⁶ cells/mL. The culture system was used for experiments between days 10 and 15.

After co-culture with CAFs, CRC cells were further co-cultured with 1 × 10^7^ cells/mL of syngeneic activated CD8^+^ T cells. The co-culture of effector cells and CRC cells was performed in 48-well polystyrene plates at a ratio of 5:1 (effector cells to CRC cells) for 8 h. After the co-culture, CD8^+^ T cells were separated from SW620 or HT29 cells by gently shaking the culture plate at 300 rpm for 3 cycles (3 min per cycle). Subsequently, an equal number of alive SW620 or HT29 cells were collected for invasion testing. Additionally, CCK-8 assays were performed on the remaining tumor cells to determine cell viability.

### Cell viability assay

CRC cells were subjected to various treatments, then harvested at a density of 1 × 10⁴ cells per well and seeded into a 96-well plate. To assess cell viability, 10 μL of CCK-8 reagent (Beyotime, Shanghai, China) was added to each well, and the plate was incubated at 37 °C for 2 h. Subsequently, the absorbance was measured at a wavelength of 450 nm using a microplate reader (Berthold, Germany). Each group was performed in triplicate wells, and each experiment was repeated at least three times to ensure the robustness and reliability of the results.

### GST protein pull-down assay

Recombinant GST-tagged MFAP2 (full-length) and His-tagged ITGB8 (full-length and fragments: 1-100 aa, 101-280 aa, 281-400 aa, 401-600 aa, 601-769 aa) were expressed in BL21 (DE3) Escherichia coli. Bacterial cultures were grown in LB medium supplemented with appropriate antibiotics at 37 °C. Protein expression was induced with 0.5 mM isopropyl-β-D-thiogalactopyranoside (IPTG) at 16 °C for 10 h. Bacterial cultures were harvested by centrifugation at 5000 rpm for 5 min at 4 °C, and the supernatant was discarded. The cell pellets were resuspended in PBS and centrifuged again at 5000 rpm for 5 min at 4 °C, followed by removal of the supernatant. The resuspended cells were lysed by sonication, and the lysates were transferred into centrifuge tubes. The lysates were centrifuged at 12,000 rpm for 10 min at 4 °C, and the supernatants were collected. To verify protein expression, a small aliquot of the supernatant was mixed with protein loading buffer, boiled at 100 °C for 3 min, and analyzed by SDS-PAGE.

GST-tagged proteins were purified using Glutathione-Sepharose 4B beads (GE Healthcare), while His-tagged proteins were purified using Ni–NTA agarose (Qiagen) following the manufacturer’s instructions (Thermo Fisher Scientific, Pittsburgh, PA, USA). The purified proteins were eluted using elution buffers containing reduced glutathione (10 mM) or imidazole (250 mM), respectively. Protein concentrations were determined using the Bradford assay, and proteins were aliquoted and stored at −80 °C until use.

Purified GST-MFAP2 (full-length) was immobilized on Glutathione–Sepharose 4B beads by incubating 20 µg of the protein with 30 µL of pre-washed beads in binding buffer (20 mM Tris-HCl, pH 7.5, 150 mM NaCl, 1 mM DTT, and 0.1% Triton X-100) (Beyotime, Shanghai, China) for 1 h at 4 °C with gentle rotation. The beads were then washed three times with binding buffer to remove unbound protein. Purified His-ITGB8 proteins (20 µg each; full-length or individual fragments) were added to the GST-MFAP2-bound beads and incubated for 2 h at 4 °C with gentle rotation. Following the incubation, the beads were washed five times with wash buffer (binding buffer supplemented with 300 mM NaCl) to remove non-specifically bound proteins. Bound proteins were eluted by boiling the beads in SDS loading buffer for 5 min at 95 °C. Eluted proteins were resolved by SDS-PAGE on a 10% polyacrylamide gel. Input samples (5% of the total input protein) were included to confirm protein expression and loading. GST alone was used as a negative control to evaluate non-specific binding of His-tagged ITGB8 proteins. All experiments were performed in triplicate.

### Western blot

The cells were rinsed three times with PBS, and then RIPA buffer (KeyGEN Bio TECH, Nanjing, China) was added to lyse the cells. The mixture was incubated on ice for 30 minutes. Centrifuge at 12,000*g* and 4 °C for 10 min, then carefully collect the supernatant. The protein concentration was determined using a BCA Protein Assay Kit (KeyGEN Bio TECH, Nanjing, China). Equal amounts of cell lysates were prepared using a protein loading buffer, and the samples were homogenized to a final concentration of 2 μg/μL. Subsequently, the lysates were subjected to electrophoresis in a 10% sodium dodecyl sulfate-polyacrylamide gel (SDS-PAGE) (Biosharp, Hefei, China) and transferred onto a polyvinylidene fluoride (PVDF) membrane (Millipore, Schwalbach, Germany) for immunoblotting.

To reduce nonspecific binding, the membrane was blocked with 5% non-fat milk at room temperature for 2 h. The membrane was then incubated with the primary antibody at 4 °C overnight. After incubation, the primary antibody was removed, and the membrane was washed three times with TBST (Servicebio, Wuhan, China), each wash lasting 10 min. The membrane was incubated with the secondary antibody for 2 h. The immunoreactive proteins were detected using an enhanced chemiluminescence (ECL) reagent (Thermo Fisher Scientific, Pittsburgh, PA, USA). The band intensities were quantitatively assessed using ImageJ software, and the relative expression levels of the target protein were normalized against the internal control protein levels. All uncropped gel and blot images corresponding to the figures in this study are included in the Supplemental Material.

### Molecular docking analysis

Protein–protein docking was performed to explore the potential interaction between MFAP2 and ITGB8. The amino acid sequences of human MFAP2 (UniProt ID: P55001) and ITGB8 (UniProt ID: P26012) were retrieved from the UniProt database (https://www.uniprot.org/), and corresponding protein structures in PDB format were obtained. Structural preprocessing was conducted using AutoDock Vina 4.2.6, including removal of water molecules and heteroatoms within 5 Å, addition of hydrogen atoms, and repair of terminal residues. The processed protein structures were subsequently submitted to the GRAMM protein–protein docking server (https://gramm.compbio.ku.edu/request), and docking results were ranked based on binding affinity scores. The top-ranked conformations were selected for further analysis. Structural visualization and interaction mapping were performed using PyMOL software (version 3.1.4.1).

### Site-directed mutagenesis

Plasmids encoding Flag-tagged MFAP2 wild-type (Flag-MFAP2-WT), His-tagged ITGB8 wild-type (His-ITGB8-WT), and His-tagged ITGB8 ARG57 mutant (His-ITGB8-ARG57) were transiently transfected into the cells using Lipofectamine 3000 (Thermo Fisher Scientific, Pittsburgh, PA, USA). An empty vector was used as a control where needed. Transfection efficiency was confirmed by Western blotting of the input samples. Forty-eight hours after transfection, the cells were harvested and lysed in cold lysis buffer (20 mM Tris-HCl, pH 7.5, 150 mM NaCl, 1% Triton X-100, 1 mM EDTA, 1 mM PMSF, and protease inhibitor cocktail) (Beyotime, Shanghai, China) for 30 min on ice. During lysis, the samples were gently vortexed every 10 min. The lysates were clarified by centrifugation at 12,000 rpm for 10 min at 4 °C, and the supernatants were collected for immunoprecipitation.

For immunoprecipitation, 1 mg of total protein was incubated with 20 µL of anti-Flag M2 agarose beads (Sigma-Aldrich) overnight at 4 °C with gentle rotation. The beads were washed five times with wash buffer (lysis buffer containing 300 mM NaCl) to remove non-specific binding proteins. Finally, the bound proteins were eluted by boiling the beads in SDS loading buffer for 5 min at 95 °C. Both the immunoprecipitated samples and input samples (5% of total lysate) were resolved on 10% SDS-PAGE gels, and a Western blot was performed to detect.

### Enzyme-linked immunosorbent assay (ELISA)

The concentration of MFAP2 in cell culture supernatants was determined using a specific MFAP2 ELISA kit (Jymbio, China). The supernatant from CAFs was collected and centrifuged at 3000*g* for 10 min to remove any cellular debris. The MFAP2 concentration was then measured according to the manufacturer’s instructions for the ELISA kit. Following the assay, the supernatant was aliquoted and stored at −80 °C for further analysis.

The concentration of 27-HC was measured in both cell culture supernatants and tumor tissues using a specific 27-HC ELISA kit (AFSBio, JL52473). The procedure for collecting the supernatant mirrored that used for MFAP2 detection.

For tumor tissue analysis, tissues were harvested from experimental animals immediately post-mortem. The tissues were thoroughly washed in ice-cold PBS to remove residual blood. Subsequently, the tumors were weighed and homogenized in an appropriate volume of ice-cold lysis buffer using a tissue homogenizer. The homogenate was then centrifuged at 10,000*g* for 15 min at 4 °C to separate the supernatant from cellular debris. The supernatant was carefully collected and stored at −80 °C until analysis.

### Immunohistochemistry (IHC)

The deparaffinized and rehydrated sections were placed in an antigen retrieval solution containing 10 mM sodium citrate (pH 6.0). The slides were heated in a microwave oven: high power for 5 min, followed by low power for 15 min. After heating, the slides were allowed to cool naturally to room temperature for antigen retrieval. PBS was used to wash the sections three times, with each wash lasting 5 min. A hydrophobic barrier was drawn around the sections using a liquid barrier pen. To block nonspecific binding, after PBS washing, normal animal non-immune serum was applied, and the sections were incubated at room temperature for 1 h, followed by gentle tapping to remove excess serum. The sections were then incubated overnight at 4 °C with the primary antibody against MFAP2 (diluted 1:300).

The primary antibody was removed, and the sections were washed with PBS three times, each wash lasting 5 min. The secondary antibody was then applied, and the sections were incubated at room temperature for 30 min in a humidity chamber. Immunostaining was performed using DAB (KeyGEN Biotech, Nanjing, China) for the color development reaction. Nuclei were stained with hematoxylin (Beyotime, Shanghai, China) for 1–3 min, followed by rinsing with running water to allow bluing. The tissue sections were subjected to dehydration and clearing treatments. An appropriate amount of neutral resin (Solarbio, Beijing, China) was applied to the slides, and coverslips were placed and gently pressed to remove air bubbles. The slides were then examined under a microscope to observe the expression of MFAP2 protein in CRC cells and the tumor stroma.

### Immunofluorescence

The coverslips (22 × 22 mm) were immersed in 75% ethanol for 2 h. According to the experimental groups, cells were seeded at a density of 5 × 10^5^ cells per well in a 6-well plate. After 24 h, the supernatant was discarded, and 500 μL of 4% paraformaldehyde (Beyotime, Shanghai, China) was added for fixation for 15 min. The cells were washed three times with PBS, followed by the addition of 0.1% Triton X-100, and left to incubate for 10 min. Blocking was performed at room temperature for 30 min using the blocking solution. The primary antibody was then added, and the cells were incubated overnight at 4 °C.

After incubation, the cells were washed with PBS, and the secondary antibody was added for 1 h in the dark. Blocking was then performed at room temperature for 30 min using serum blocking solution. The blocking solution was discarded, and the secondary primary antibody was added. The cells were incubated overnight at 4 °C. After incubation, the cells were treated with fluorescence-conjugated secondary antibody and incubated in the dark for 1 h. The cells were then washed three times with PBS, placed on glass slides, and mounted with DAPI (KeyGEN Biotech, Nanjing, China). Observation was performed under a laser scanning confocal microscope, and random fields of view were selected for imaging.

The antibody information and dilution ratios used in this study are provided in Supplementary Tables 2 and 3.

### Invasion assay

The cold serum-free medium and Matrigel stock solution were mixed at a ratio of 5:1, gently mixed by pipetting, and kept on ice. In total, 50 μL of the diluted Matrigel gel was added to the upper chamber of the transwell, gently tilted to ensure even spreading, and incubated at 37 °C for 4 h in a cell culture incubator. After the Matrigel had fully solidified, the unsolidified liquid in the upper chamber was aspirated, and 70 μL of culture medium was gently added. The chamber was then incubated at 37 °C for 30 min. The transwell chamber was removed, and the liquid in the upper chamber was carefully aspirated. Then, 200 μL of single-cell suspension was gently added along the wall of the upper chamber containing 0.5% FBS (to avoid the interference from cell proliferation in the process of cell invasion). Three replicates were set up for each condition. In total, 600 μL of culture medium containing 10% FBS was added to the lower chamber of each well in a 24-well plate.

The upper chamber was washed three times with PBS, and the lower chamber was immersed in PBS for 5 min each time. In total, 700 μL of methanol (SCRC, Shanghai, China) was added to the lower chamber of a new 24-well plate, and the cleaned transwell chambers were placed into the plate and fixed for 30 min. The methanol was discarded, and 700 μL of 1% crystal violet (Beyotime, Shanghai, China) was added to the lower chamber. The chamber was then left to stand for 30 min. Gently wipe off the cells that have not passed through the inner layer of the chamber using a damp cotton swab. Then, invert the chamber and allow it to air-dry naturally or dry it in a low-temperature oven. Subsequently, cell counting was performed using an inverted microscope (Nikon, Japan).

### Flow cytometry

CD8⁺ T cells were washed with PBS and centrifuged at 2000 rpm for 5 min. After counting, 1 × 10⁵ cells were resuspended in 0.5 mL of binding buffer and stained with Annexin V-FITC (5 μL) and propidium iodide (PI, 5 μL). The samples were incubated at room temperature in the dark for 15 min, followed by analysis on a flow cytometer (Beckman Coulter, USA) to assess apoptosis.

For surface marker analysis, CD8⁺ T cells were incubated with anti-CD8 antibody (5 μL; Proteintech, Rosemont, IL, USA) at room temperature for 30 min in the dark. Cells were then washed with PBS to remove unbound antibodies and resuspended in 400 μL PBS for flow cytometric detection.

Tumor tissues derived from the cecum and liver were mechanically dissociated and enzymatically digested at 37 °C in a rotating incubator (250 rpm) for 1 h. The digestion was interrupted every 20 min by tube inversion to ensure uniform breakdown. After digestion, the cell suspension was filtered through a 100 μm mesh to remove debris. The filtrate was centrifuged at 1200 rpm for 5 min, and the resulting pellet was washed twice with PBS. Isolated cells were then processed for downstream assays as described above.

### Statistical analysis

The sample size determination was based on experimental designs reported in previously published high-quality studies with similar methodologies [27–30]. Statistical analyses were performed using SPSS software (version 26.0, Chicago, IL, USA) and GraphPad Prism (version 9.5, San Diego, CA, USA). Survival curves were compared using the log-rank test. Normality of the data distribution was assessed using the Shapiro–Wilk test, and homogeneity of variances was evaluated by the *F*-test (for two-group comparisons) or Levene’s test (for multiple-group comparisons). For comparisons between two groups, a two-tailed unpaired Student’s *t*-test was introduced. For multiple group comparisons (*n* ≥ 3), one-way analysis of variance (ANOVA) was conducted, followed by the LSD post hoc test. In cases where variance homogeneity was not met, Welch’s ANOVA was applied, followed by the Games–Howell post hoc test. Data are expressed as mean ± standard deviation (SD) from at least three independent experiments. A *P*-value < 0.05 was considered statistically significant. During experiments, investigators responsible for outcome assessment and data analysis were blinded to group allocation. Randomization and group assignment were performed by a separate researcher not involved in data collection, ensuring unbiased assessment.

## Results

### MFAP2 expression is upregulated in CRC and CAFs

**Fig. 1:**
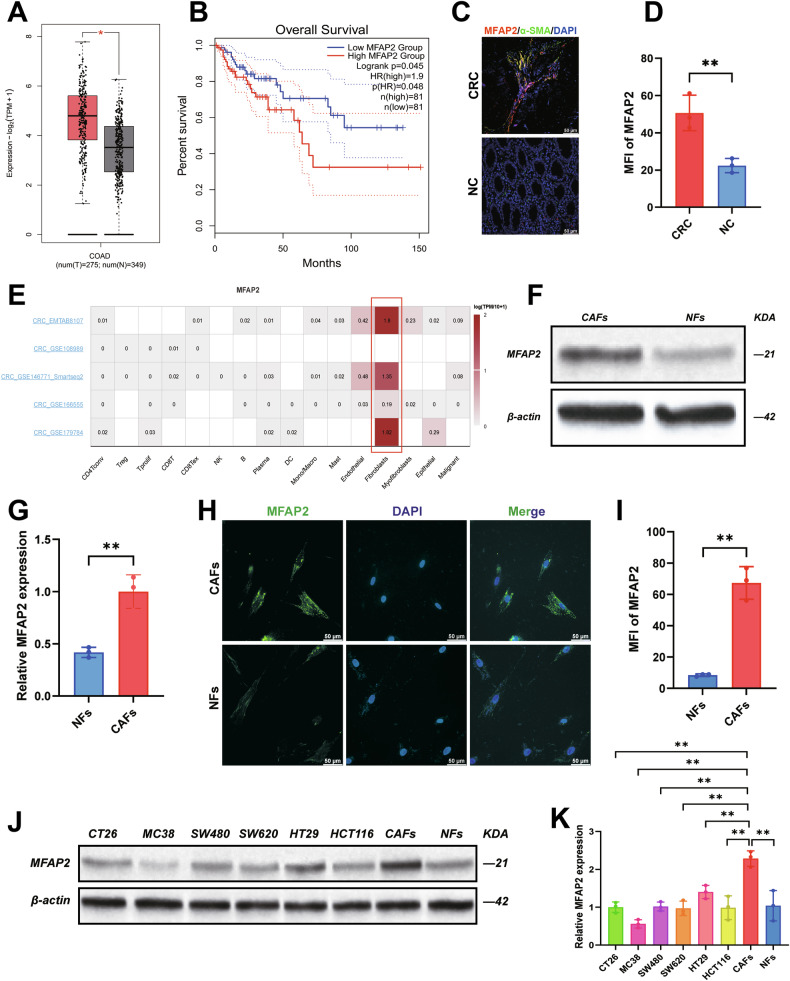
MFAP2 expression is upregulated in CRC and CAFs. **A** Gene expression database analysis of MFAP2 mRNA in CRC as compared to normal colon (NC) (the data presented in the box plots are represented using the interquartile range method). **B** MFAP2 gene expression is associated with overall survival in CRC. **C** Representative images of MFAP2 immunohistochemical photos of NC/CRC tissue sections (scale bar, 50 μm). **D** Quantitative analysis of IHC staining of MFAP2 via determined the accumulated optical density (AOD) of the positive area. **E** The enrichment of MFAP2 in different cell types in the CRC scRNA-seq dataset. **F** Representative Western blot bands of MFAP2 in primary NFs and CAFs. **G** Quantitative analysis of the grayscale value of MFAP2 (β-Actin was set as internal control) in primary NFs and CAFs. **H** Representative images of immunofluorescence staining depicting MFAP2 expression in NFs and CAFs. **I** Quantitative analysis of the optical density of MFAP2. **J** Representative Western blot bands of MFAP2 expression in various colon cancer cell lines, CAFs and NFs. **K** Representative Western blot bands of MFAP2 in various colon cancer cell lines, CAFs and NFs. Data are shown as mean ± SD, *n* = 3 or 6, *P < 0.05, **P < 0.01.

First, we found through the TCGA database that MFAP2 is significantly overexpressed in CRC tissues compared to adjacent normal tissues (Fig. 1A, *P* < 0.05). In addition, survival analysis revealed that high MFAP2 expression is associated with poor prognosis in CRC patients (Fig. 1B, *P* = 0.048). Immunofluorescence staining of tumor clinical samples revealed that MFAP2 expression is significantly higher in the fibroblasts of CRC tissues compared to that of normal colon tissues (Fig. 1C, D, *P* < 0.01).

Using the tumor immune single-cell hub (TISCH 2.0) sequencing database (http://tisch.comp-genomics.org/), we found that MFAP2 is significantly enriched in fibroblasts (Fig. 1E). Next, we isolated primary CAFs and normal fibroblasts (NFs) from CRC and adjacent normal colon tissues (NC), and performed MFAP2 expression analysis using Western blotting and immunofluorescence (the identification of human and mouse NFs and CAFs was shown in Fig. S1). The results showed that the expression of MFAP2 was significantly higher in CAFs compared with NC (Fig. 1F–I, *P* < 0.01). Subsequently, the expression of MFAP2 in various CRC cell lines and fibroblasts was determined using Western blotting, the results of which revealed that, compared to tumor cell lines and NFs, MFAP2 was highly expressed by CAFs (Fig. 1J, K, *P* < 0.01). Moreover, we also performed subtype annotation of CAFs based on established marker genes corresponding to distinct CAF subsets. This analysis revealed that CAFs within the included clinical tumor samples could be classified into myCAF, matrix-associated CAF (matCAFs), iCAF, antigen-presenting CAF (apCAF), proliferative CAF (proCAF), metabolic CAF, and normal fibroblasts (NF) populations. As can be seen in Fig. S2, when MFAP2-positive CAFs were highlighted on the UMAP projection, matrix CAFs contributed predominantly to the overall MFAP2 expression, although MFAP2 expression was observed across various CAF subpopulations.

### CAFs-derived MFAP2 promotes CRC proliferation and invasion

**Fig. 2:**
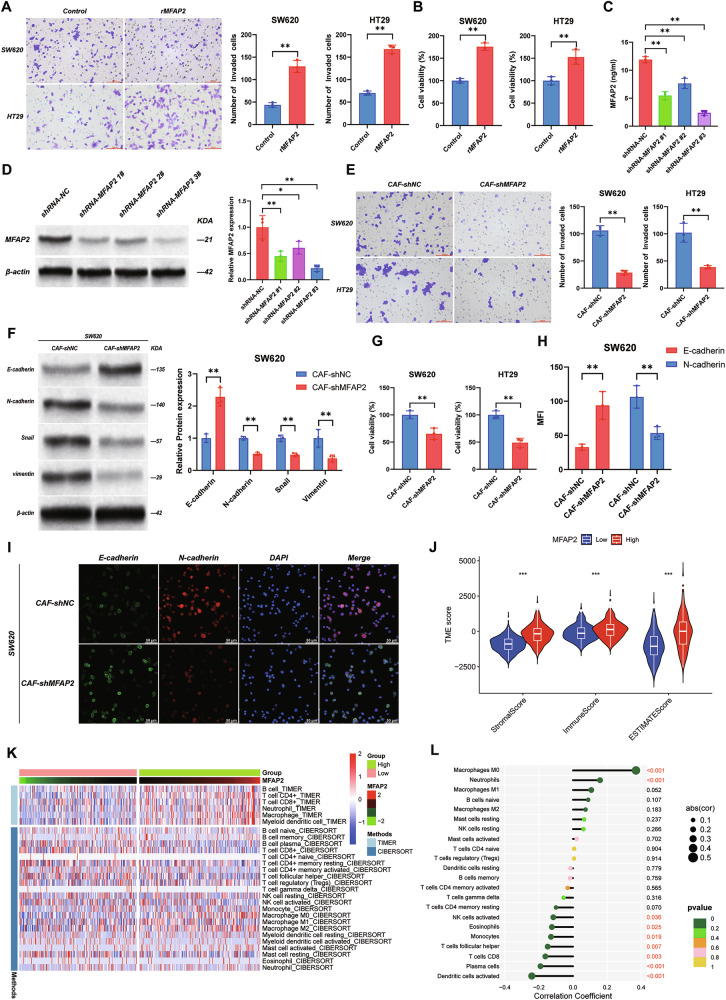
MFAP2 derived from CAFs promotes proliferation and invasion in CRC. **A** The invasion of SW620/HT29 cells after co-incubation with MFAP2. **B** The proliferation of SW620/HT29 cells after MFAP2 incubation. **C** MFAP2 protein expression after transfection with shRNA. **D** The secretion of MFAP2 after transfection with shRNA. The invasion (**E**) and proliferation (**G**) of SW620/HT29 cells after co-culture with MFAP2 knockdown CAFs. **F** Western blot detection of E-cadherin, N-cadherin, Vimentin, and Snail protein expression levels in SW620 cells after coculture with CAFs-shMFAP2. **H**, **I** Immunofluorescence detection of E-cadherin, N-cadherin protein expression levels in SW620 cells after co-culture with CAFs-shMFAP2. **J** TIMER analyses MFAP2 scores in the immune microenvironment. **K** CIBERSORT analyses the relationship between MFAP2 and immune cells. **L** TCGA database analysis of MFAP2 in relation to immune cells. Data are shown as mean ± SD, *n* = 3, *P < 0.05, **P < 0.01, ***P < 0.001.

To investigate whether MFAP2 affects CRC progression, we introduced recombinant human MFAP2 protein (rMFAP2) and evaluated its impact on CRC cell viability and invasion. The results showed that upon addition of MFAP2, the cancer cells exhibited significantly enhanced invasion and increased viability (Fig. 2A, B, *P* < 0.01), suggesting that MFAP2 promotes the motility of CRC cells. Furthermore, to verify whether the pro-tumorigenic effect of CAFs on CRC is mediated by MFAP2, we established a co-culture system using CAFs and CRC cell lines (SW620 and HT29). Among the three candidate sequences tested, we selected shMFAP2 3# for further study, as it effectively downregulated MFAP2 protein levels (Fig. 2C, D, *P* < 0.01).

The coculture results indicated that knockdown of MFAP2 in CAFs inhibited the invasion ability (Fig. 2E, *P* < 0.01) and viability (Fig. 2G, *P* < 0.01) of SW620 and HT29 cells. Subsequently, we examined the epithelial–mesenchymal transition (EMT) markers in CRC cells and found that knockdown of MFAP2 in CAFs could reverse the EMT process induced by CAFs (Fig. 2F, Fig. S3A, B, Fig. S4 and Fig. S5, *P* < 0.01). The immunofluorescence results were consistent with the Western blotting results (Fig. 2H, I, Fig. S3C, D, Fig. S4 and Fig. S5, *P* < 0.01).

As shown in Fig. S6A, MFAP2 is indeed highly expressed and secreted by CAFs, in contrast to its relatively low expression in tumor cells (*P* < 0.001). Functional assays further demonstrated that the introduction of neutralizing antibody against MFAP2 significantly attenuated the invasion of tumor cells (Fig. S6B, C, *P* < 0.01). These findings confirm the central role of CAFs-derived MFAP2 in enhancing tumor progression and validate its potential as a therapeutic target. Therefore, these findings suggest that MFAP2 derived from CAFs is a key factor driving the growth and metastasis of CRC cells.

Next, we analyzed the relationship between MFAP2 and the immune microenvironment in colon cancer using the TCGA database, and found that high expression of MFAP2 was significantly associated with the immune microenvironment (Fig. 2J, P < 0.001). The analysis revealed that high expression of MFAP2 was negatively correlated with CD8^+^ T cells (Fig. 2K, L). Based on the median expression level of MFAP2, samples were stratified into MFAP2-low (MFAP2^-^CAFs) and MFAP2-high (MFAP2^+^CAFs) groups, and the expression of MFAP2 in MFAP2^-^CAFs and MFAP2^+^CAFs was highlighted in the UMAP plots (Fig. S7A–C). Subsequently, we analyzed the correlation between high and low MFAP2 expression groups and immune cells, which showed that CD8^+^ T cells were mainly enriched in the MFAP2^−^CAFs group (Fig. S7D, E). Correlation analysis demonstrated a negative association between MFAP2 expression in CAFs and the abundance of CD8^+^ T cells within the TME (*r* = −0.407, Fig. S7F, G), suggesting that elevated MFAP2 expression may contribute to an immune-excluded phenotype by limiting cytotoxic T cell infiltration. Next, we established a coculture system to evaluate the impact of MFAP2 on cell interactions within the TME. The coculture results showed that MFAP2 secreted by CAFs significantly enhanced the invasion ability (Fig. S3E–G, *P* < 0.01) and viability (Fig. S3H, I, *P* < 0.01) of tumor cells under the stress of active CD8^+^ T cells. These results suggest that MFAP2 might promote CRC progression through CD8^+^ T cell-mediated antitumor responses.

Meanwhile, we have conducted additional in vivo experiments using nude mice to further validate the functional role of MFAP2^+^CAFs in tumor progression after excluding the influence of immune cells. Both liver metastasis and subcutaneous tumor models were established using SW620 cells. As shown in Fig. S8A, B, in the liver metastasis model, co-injection of CAFs with SW620 cells markedly increased the number of visible liver nodules compared to SW620 cells alone (*P* < 0.01). However, knockdown of MFAP2 in CAFs (CAFs-shMFAP2) significantly reduced metastatic burden (*P* < 0.01), and this inhibitory effect was partially reversed by the addition of rMFAP2 (*P* < 0.01). Consistently, in the subcutaneous tumor model (Fig. S8C, D), SW620 cells co-injected with CAFs-shNC exhibited enhanced tumor growth (*P* < 0.01), whereas CAFs-shMFAP2 markedly suppressed tumor volume (*P* < 0.01), which was significantly reversed by the administration of rMFAP2, restoring tumor growth to a level comparable to the control.

### CAFs-derived MFAP2 promotes CRC progression in vivo

**Fig. 3:**
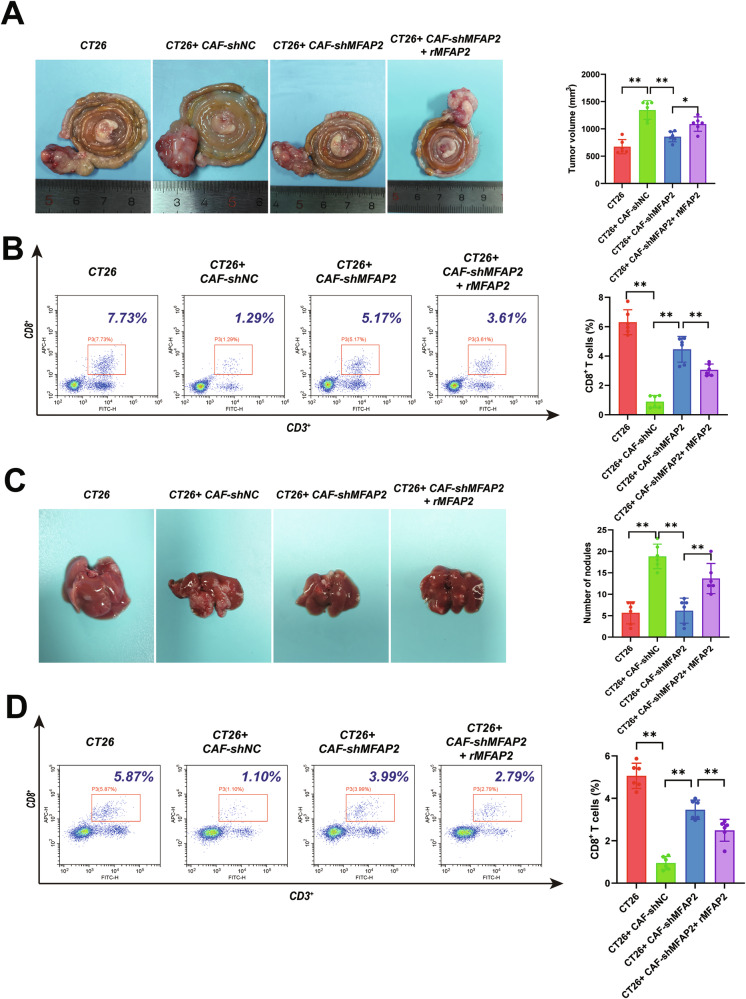
MFAP2 derived from CAFs promoted tumor growth/metastasis and immune evasion in vivo. **A** Gross appearance of colorectal tumors using the Swiss roll technique and quantification of tumor volume. **B** Representative images and quantification of flow cytometric analysis of CD3^+^ CD8^+^ T cells in the orthotopic tumor. **C** Gross appearance of liver metastasis and quantification of the number of liver tumor nodules. **D** Representative images and quantification of flow cytometric analysis of CD3^+^CD8^+^ T cells in metastatic liver tumor nodules. Data are shown as mean ± SD, *n* = 6, *P < 0.05, **P < 0.01.

To evaluate the effect of CAFs-derived MFAP2 on the progression of CRC in vivo, we established mouse models of orthotopic colon cancer and liver metastasis. CAFs with MFAP2 knockdown were cocultured with CT26-Luc cells for 10 days, followed by injection (1 × 10^6^ CT26: 1 × 10^6^ CAF) into the cecum of BALB/c mice. The tumor volume in the cecum was measured at the study endpoint (As can be seen in Fig. S9, the tumor volume changes are primarily attributed to alterations in tumor cell abundance). As shown in Fig. 3A, compared to mice injected with CT26 cells alone, the tumor volume in the CT26 + CAFs group was significantly increased (*P* < 0.01). After knockdown of MFAP2 in CAFs, the tumor volume was significantly reduced, while injection of rMFAP2 resulted in a significant increase in tumor volume (*P* < 0.05). In addition, we quantified the number of infiltrating lymphocytes in the tumors using flow cytometry. As shown in Fig. 3B, compared to the CT26 group, the proportion of CD8^+^ T cells was significantly reduced in the CT26 + CAFs-shNC group (*P* < 0.01). After knockdown of MFAP2 in CAFs, the proportion of infiltrating CD8^+^ T cells was significantly increased (*P* < 0.01). However, injection of rMFAP2 reversed this effect and blocked the immune infiltration induced by shMFAP2 (Fig. 3B, Fig. S10A, B).

Similarly, in the liver metastasis model, we injected CT26 cells along with CAFs into the spleens of mice and measured the number of tumor nodules in the liver at the study endpoint. As shown in Fig. 3C, knockdown of MFAP2 in CAFs significantly inhibited liver tumor growth (*P* < 0.01), whereas injection of rMFAP2 resulted in a significant increase in the number of liver tumor nodules (*P* < 0.01). Additionally, we also quantified the number of lymphocytes in the liver metastatic tumors using flow cytometry. As shown in Fig. 3D, compared to the CT26 + CAFs-shNC group (*P* < 0.01), knockdown of MFAP2 in CAFs significantly increased the proportion of infiltrating CD8^+^ T cells (*P* < 0.01). However, injection of rMFAP2 led to a significant reduction in the number of infiltrating CD8^+^ T cells, indicating that inhibition of MFAP2 might suppress liver metastasis of the tumor by raising the infiltration of CD8^+^ T cells. The in vivo imaging results were consistent with the above findings (Fig. S11A–C). What’s more, when CT26 was injected alone, the addition of rMFAP2 also reduced the infiltration of CD8^+^ T cells and increased the orthotopic tumor volume (Fig. S12A–D*P* < 0.01). These findings indicate a possible association between CAFs-derived MFAP2 and reduced CD8⁺ T cell function, which may be linked to CRC progression and metastasis.

### CAFs-derived MFAP2 promotes CRC progression through ITGB8

**Fig. 4:**
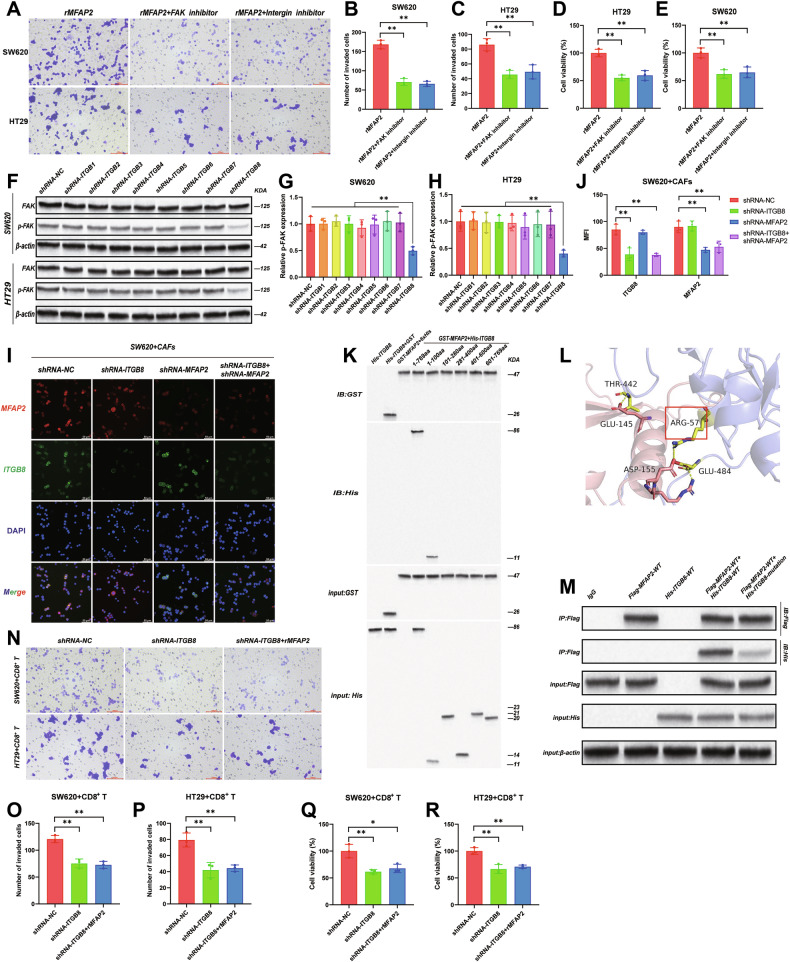
CAFs-derived MFAP2 promotes CRC progression through ITGB8. **A** Representative image of the Transwell invasion assay using SW620 and HT29. Quantitative analysis of the number of SW620 cells (**B**) and HT29 cells (**C**) that invaded through the membrane. Cell viability of SW620 cells (**D**) and HT29 cells (**E**) after co-incubation with rMFAP2, FAK inhibitor or integrin inhibitor. **F** FAK and p-FAK expression after transfection with shRNA–ITGB1–ITGB8. Quantitative analysis of phosphorylated FAK (p-FAK) relative to total FAK in SW620 cells (**G**) and HT29 cells (**H**). **I** Representative immunofluorescence images showing the expression and localization of ITGB8 (green) and MFAP2 (red) in SW620 cells. **J** Quantification of immunofluorescence signal intensity of ITGB8 and MFAP2. Immunofluorescence detection of the interaction between MFAP2 and ITGB8 in SW620 cells from each group. **K** GST-pulldown assays were performed to detect the interaction between MFAP2 and ITGB8. **L** Molecular docking visualizes the site of interaction of MFAP2 with ITGB8. **M** Site-directed mutagenesis experiments validate the effect of ARG57 on the interaction between ITGB8 and MFAP2. **N** Representative images showing invasive cells in the transwell invasion assay after the ITGB8 knockdown and rMFAP2 supplement after the co-culture with CAFs and CD8^+^ T cells successively. Quantification of invaded SW620 cells (**O**) and HT29 cells (**P**) in the transwell assay. Cell viability of SW620 cells (**Q**) and HT29 cells (**R**) was quantitatively assessed using CCK8 after the co-culture with CAFs and CD8^+^ T cells successively. Data are shown as mean ± SD, *n* = 3, *P < 0.05, **P < 0.01.

An important step in tumor cell migration is the adhesion to the ECM through specific focal adhesion sites, a process primarily mediated by integrin signaling [31, 32]. The existing literature reports that MFAP2 in gastric cancer can promote tumor cell migration through the integrin α5β1/FAK/ERK pathway [24]. Therefore, we first validated whether MFAP2 affects CRC progression through the integrin-FAK pathway. CRC cell lines were incubated with rMFAP2 and subsequently treated with FAK inhibitor and integrin inhibitor followed by invasion and cell viability assay, the results of which showed that cell invasion and cell viability in SW620 and HT29 were both significantly reduced under the interference of FAK inhibitor and integrin inhibitor (Fig. 4A–E, *P* < 0.01), suggesting that MFAP2 may exert its effect through the integrin pathway.

Since integrins are transmembrane receptors that mediate the connection between cells and the ECM, typically composed of α and β subunits [33], we primarily focused on investigating the interaction between MFAP2 and the integrin β subunit in this study (the distribution of ITGB8 is mainly enriched in cancer cell clusters, Fig. S13). To investigate the role of integrins in MFAP2-mediated signaling, ITGB1–ITGB8 were individually knocked down in CRC cell lines (the knockdown efficiency was verified in Fig. S14A–H), followed by incubation with rMFAP2. Among them, knockdown of ITGB8 markedly attenuated FAK phosphorylation (Fig. 4F–H, *P* < 0.01), suggesting that ITGB8 is critical for MFAP2-induced FAK activation.

Subsequently, CRC cells were co-cultured with CAFs, and immunofluorescence analysis revealed co-localization of MFAP2 and ITGB8 (Fig. 4I). When either of the proteins was knocked down, the co-localization was weakened (Fig. 4I, J, Fig. S14I, J, *P* < 0.01). These results suggest that MFAP2 might exert its biological effects through the FAK pathway via binding to the integrin membrane receptor ITGB8 (knockdown of ITGB1–ITGB8 showed no direct effect on basal cell viability under standard culture conditions, Fig. S14K).

As shown in Fig. 4K, MFAP2 primarily interacts with the 1–100 amino acid region of ITGB8, where the specific interaction site, ARG57, was predicted through protein docking using the online website (http://gramm.compbio.ku.edu/) and online visualization (http://www.ebi.ac.uk/msd-srv/prot_int/) (Fig. 4L). Next, it was found that the interaction between ITGB8 and MFAP2 was significantly weakened after mutating ARG57 in ITGB8 by site-directed mutagenesis experiments (Fig. 4M).

Next, we co-cultured CRC cells with CAFs and CD8^+^ T cells, successively. As shown in Fig. 4N–P, knockdown of ITGB8 in CRC cells significantly reduced the invasive ability of tumor cells that remained viable after coculture with CD8⁺ T cells (*P* < 0.01). However, when rMFAP2 was added, there were no significant changes in tumor cell invasion. Comparable results were observed when evaluating cell viability (Fig. 4Q, R). These results suggested that MFAP2 might primarily bind to ITGB8 and exerts its biological effects on cell invasion/proliferation and immune evasion in a FAK-dependent manner.

### MFAP2 promotes CRC progression through the ERK1/2-ETS2 pathway

Previous studies have reported that FAK can receive signals from transmembrane receptors on the cell surface, including integrins, cytokines, growth factors, and G protein-coupled receptors. Subsequently, FAK is activated in various cellular processes, initiating a cascade of downstream signaling events [34]. For example, FAK promotes the growth and metastasis of liver cancer stem cells through the extracellular signal-regulated kinase (ERK1/2) pathway [35]. ERK1/2 plays a crucial role in regulating cell migration by relaying signals from FAK in various cell types [36]. ETS family transcription factors are key downstream effectors of the RAS/MAPK signaling pathway, with ETS1 and ETS2 being particularly important [37, 38]. ETS1 and ETS2 are phosphorylated by ERK1/2 at Thr38 and Thr72, respectively, and thus serve as alternative targets of the RAS/MAPK pathway [39]. Here, we primarily discuss the role of ETS2 in CRC.

**Fig. 5:**
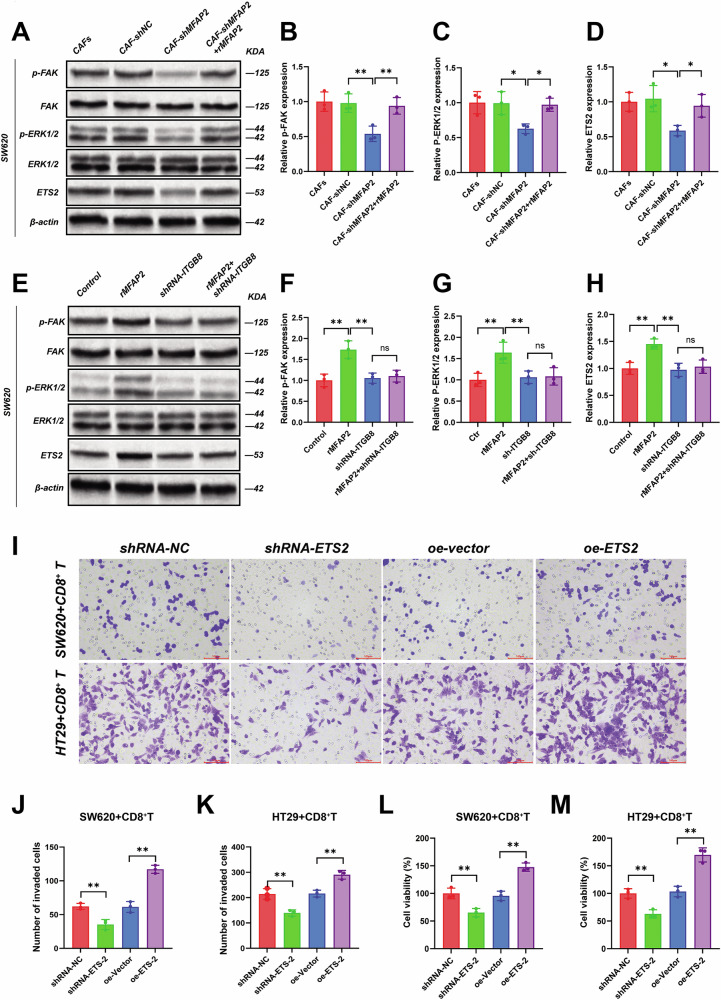
CAFs-derived MFAP2 promotes CRC progression through the ETS2 pathway. **A** Western blot analysis of p-FAK, total FAK, p-ERK1/2, total ERK1/2, and ETS2 expression in SW620 cells co-cultured with CAFs (with MFAP2 knockdown or/and rMFAP2 incubation). Quantification of p-FAK (**B**), p-ERK1/2 (**C**), and ETS2 (**D**) protein expression levels normalized to total protein or β-actin. **E** Western blot analysis of p-FAK, total FAK, p-ERK1/2, total ERK1/2, and ETS2 in SW620 cells treated with rMFAP2 or sh-ITGB8, or the combination. Quantification of p-FAK (**F**), p-ERK1/2 (**G**), and ETS2 (**H**) expression. (**I**) Representative images of Transwell invasion assays in SW620 and HT29 cells co-cultured with CD8⁺ T cells after ETS2 knockdown or ETS2 overexpression. Scale bars = 50 μm. Quantification of the number of invaded SW620 (**J**) and HT29 (**K**) cells under the indicated conditions. CCK-8 assay showing the cell viability of SW620 (**L**) and HT29 (**M**) cells cocultured with CD8⁺ T cells after ETS2 modulation. Data are presented as mean ± SD. *P < 0.05, **P < 0.01.

First, we co-cultured CAFs with CRC cell lines and found that MFAP2 derived from CAFs significantly increased the levels of p-FAK, p-ERK1/2, and ETS2. When MFAP2 was knocked down in CAFs, this effect was reversed (Fig. 5A–D, Fig. S15A–D, *P* < 0.01), which was rescued via the addition of rMFAP2 (*P* < 0.01). Next, we knocked down ITGB8 in CRC cell lines and incubated them with rMFAP2. rMFAP2 was found to increase the levels of p-FAK, p-ERK1/2, and ETS2 (Fig. 5E–H, Fig. S15E–H, *P* < 0.01). Moreover, the exogenous rMFAP2 had no effect on the levels of p-FAK, p-ERK1/2, and ETS2 after ITGB8 knockdown (Fig. 5E–H, Fig. S15E–H, *P* > 0.05), which suggested that MFAP2 might primarily participate in CRC progression through the ITGB8–FAK–ERK1/2–ETS2 dependent pathway.

Liver metastasis model using BALB/c mice via spleen injection was established to assess the impact of ITGB8 knockdown in tumor cells under different conditions. As shown in Fig. S16A, B, co-injection with CAFs or supplementation with rMFAP2 significantly increased the number of liver metastatic nodules in comparison with the CT26-shNC group (*P* < 0.01). Compared with the CT26-shNC + CAFs or CT26-shNC + rMFAP2 group, knockdown of ITGB8 in CT26 cells significantly reduced the number of gross liver metastases (*P* < 0.01), suggesting that CAFs-derived MFAP2 might increase metastatic burden in an ITGB8-dependent manner.

To further assess the immune evasion, we analyzed the infiltration of CD8⁺ T cells within liver metastases by flow cytometry using BALB/c. As shown in Fig. S16C, D, co-injection with CAFs or supplementation with rMFAP2 significantly reduced the infiltration of CD8^+^ T cells in comparison with the CT26-shNC group (*P* < 0.01), which was significantly reversed by ITGB8 knockdown, indicating that CAFs-secreted MFAP2 might promote tumor progression, and that ITGB8 knockdown could restore immune infiltration, thereby inhibiting metastatic outgrowth.

Next, the efficiency of ETS2 shRNA and overexpression was verified in advance, and the knockdown of ETS2 showed no obvious effect on cancer cell lines’ viability (Fig. S15I–L and Fig. S17A, B). We cocultured CRC cell lines with CD8^+^ T cells; the results showed that knockdown of ETS2 significantly reduced tumor cell viability and invasion under the pressure of activated CD8^+^ T cells (Fig. 5I–M, *P* < 0.01), which was reversed by overexpression of ETS2 (*P* < 0.01).

To further investigate the involvement of ETS2 in the MFAP2-mediated pro-tumorigenic effect, ETS2 was silenced in CT26 cells. As shown in Fig. S11D–F, silencing ETS2 in CT26 cells abrogated the enhancement of liver metastasis and orthotopic tumor growth induced by CAFs or rMFAP2 (*P* < 0.01). Quantitative analysis confirmed that both CAF- and rMFAP2-mediated promotion of tumor burden was dependent on ETS2 expression in tumor cells, as evidenced by significantly reduced bioluminescent signals in the shETS2 groups (*P* < 0.01). These data demonstrate that CAFs-derived MFAP2 might promote CRC progression and metastasis via a CAF–tumor–ETS2 axis.

### ETS2 inhibits CD8^+^ T cells through the cholesterol metabolism pathway

**Fig. 6:**
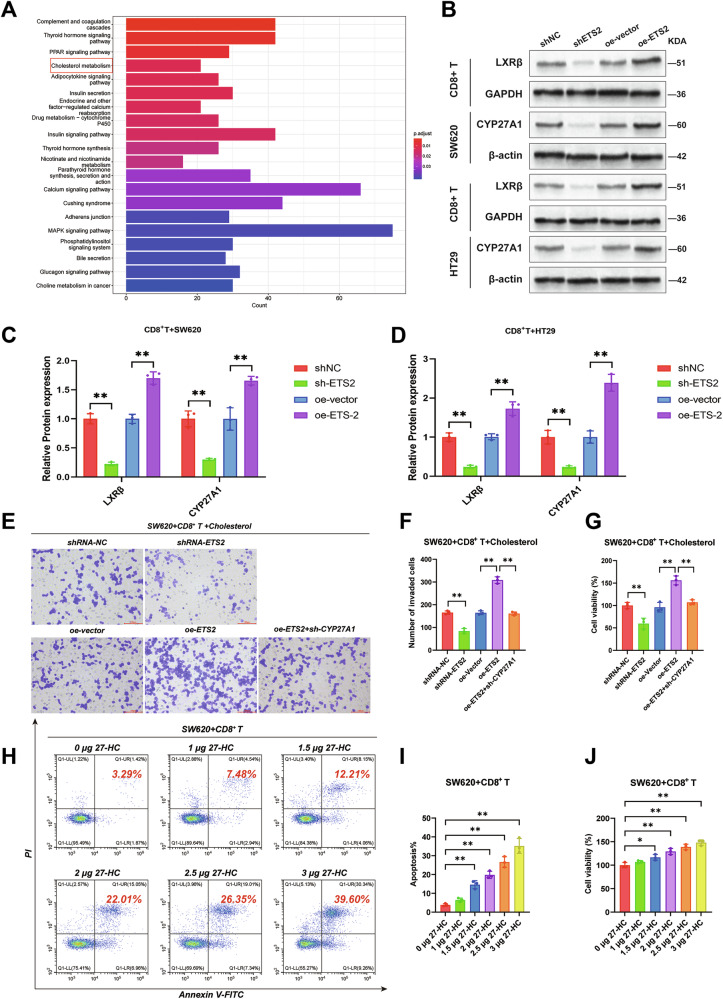
ETS2 inhibits CD8^+^ T cells through the cholesterol metabolism pathway. **A** Enrichment analysis of KEGG pathway in CRLM. **B** Representative immunoblot images of CYP27A1 and LXRβ protein expression in tumor cell lines or CD8^+^ T cells following ETS2 knockdown or overexpression in tumor cells. Quantification of CYP27A1 and LXRβ protein expression after knockdown or overexpression of ETS2 in SW620 cells (**C**) and HT29 cells (**D**). **E** Representative images of invaded cells stained with crystal violet in the Transwell invasion assay. **F** Quantification of invaded cells in the Transwell invasion assay. **G** Quantification of cell viability in SW620 under different intervention conditions. **H** Representative flow cytometry plots of apoptosis staining in CD8⁺ T cells treated with different concentrations of 27-HC. **I** Quantification of apoptotic CD8⁺ T cells following treatment with increasing concentrations of 27-HC. **J** Cell viability of SW620 was quantified under various treatment conditions. Data are shown as mean ± SD, *n* = 3, *P < 0.05, **P < 0.01.

By analyzing the GEO database (GSE41258) and performing KEGG pathway analysis, we found that CRLM is highly associated with cholesterol metabolism (Fig. 6A). Moreover, the cholesterol metabolic enzyme Cytochrome P450, family 27, subfamily A, polypeptide 1 (CYP27A1) was identified within the cholesterol metabolism pathway. CYP27A1 is a widely expressed mitochondrial enzyme that catalyzes the hydroxylation of cholesterol at the C-27 position, producing 27-hydroxycholesterol (27-HC) and bile acids. 27-HC is a type of oxysterol [40, 41].

Cholesterol metabolism significantly contributes to cancer progression. For instance, cholesterol-derived tumor metabolites such as 6-oxo-cholesterol-3β and 5α-diol bind to glucocorticoid receptors in breast cancer, thereby promoting tumor growth [42]. Other metabolites also facilitate tumor cell proliferation, migration, and invasion [43, 44]. Cholesteryl esters (CE) and oxysterols are abundant cholesterol metabolites in the TME, including 27-HC and 24-hydroxycholesterol (24-HC), which exert broad functions by binding to nuclear receptors such as LXR and ROR49 [45, 46]. Cholesterol-derived oxysterols exert different effects on tumor-infiltrating immune cells. For example, in breast cancer, 27-HC can attract polymorphonuclear neutrophils and γδ T cells, while simultaneously depleting CD8^+^ T cells, thereby promoting tumor metastasis [47, 48]. Activated T cell proliferation also relies on elevated cholesterol metabolism levels. SREBP2 signaling has been shown to be crucial for CD8^+^ T cell proliferation and effector function, while LXR signaling negatively regulates T cell activation [49, 50]. Endogenous activators of Liver X Receptors (LXRα and LXRβ) are oxysterols and the cholesterol biosynthesis pathway. LXRα is highly expressed in the liver, adipose tissue, and macrophages, while LXRβ is widely expressed [51, 52]. Therefore, oxysterols abundant in the TME may suppress T cell-mediated anti-tumor immunity by activating LXRβ.

First, we predicted the transcription factor of CYP27A1 to be ETS2 using PROMO. Next, we co-cultured CRC cells with CD8^+^ T cells, and upon knockdown of ETS2, the levels of CYP27A1 in tumor cells and LXRβ in CD8^+^ T cells were significantly reduced (Fig. 6B–D, *P* < 0.01). However, overexpression of ETS2 resulted in a significant increase in the expression levels of both proteins (*P* < 0.01). Next, we cocultured CRC cells with CD8⁺ T cells in the presence of cholesterol to evaluate the inhibitory effect of CD8⁺ T cells on tumor cells. The results showed that knockdown of ETS2 reduced tumor cell invasion (*P* < 0.01) and viability (*P* < 0.01) after the stress of CD8^+^ T cells. While overexpression of ETS2 significantly enhanced tumor cell invasion and viability (Fig. 6E–G, Fig. S18A–C, *P* < 0.01). However, knockdown of CYP27A1 resulted in a marked decrease in tumor cell invasion and viability (*P* < 0.01), indicating that overexpression of ETS2 might exert effects through the ETS2–CYP27A1 axis. Subsequently, CD8⁺ T cells were treated with a gradient of 27-HC concentrations. A dose-dependent increase in CD8⁺ T cell apoptosis was observed (*P* < 0.01), accompanied by a significant enhancement in tumor cell viability (Fig. 6H–J, Fig. S18D–F, *P* < 0.05 and *P* < 0.01).

To elucidate the role of MFAP2 in regulating cholesterol metabolite production within the TME, we first measured 27-HC levels in co-culture supernatants of CT26 colon carcinoma cells and CAFs with stable knockdown of MFAP2. ELISA analysis revealed that CT26 cells cocultured with control CAFs exhibited significantly elevated 27-HC levels compared to CT26 cells alone (Fig. S19A, *P* < 0.01). In contrast, MFAP2 silencing in CAFs (shMFAP2) markedly reduced 27-HC concentrations. Importantly, reconstitution with rMFAP2 protein (shMFAP2 + rMFAP2) partially restored 27-HC production (*P* < 0.01).

We next examined whether this regulatory effect persists in vivo. Orthotopic tumor models were established by co-injecting CT26 cells with corresponding CAF subtypes into syngeneic mice. Consistent with the in vitro findings, tumors formed in the presence of MFAP2-deficient CAFs exhibited significantly lower intratumoral 27-HC levels compared to those co-injected with control CAFs (*P* < 0.01), while rMFAP2 supplementation restored 27-HC accumulation in tumor tissues (Fig. S19B, *P* < 0.01).

These results indicate that MFAP2-expressing CAFs are a critical regulator of tumor-derived 27-HC, suggesting a potential mechanism by which the stromal compartment modulates cholesterol metabolism and immunomodulatory signaling in the TME.

### MFAP2 promotes CRC progression in vivo through the ETS2 pathway

**Fig. 7:**
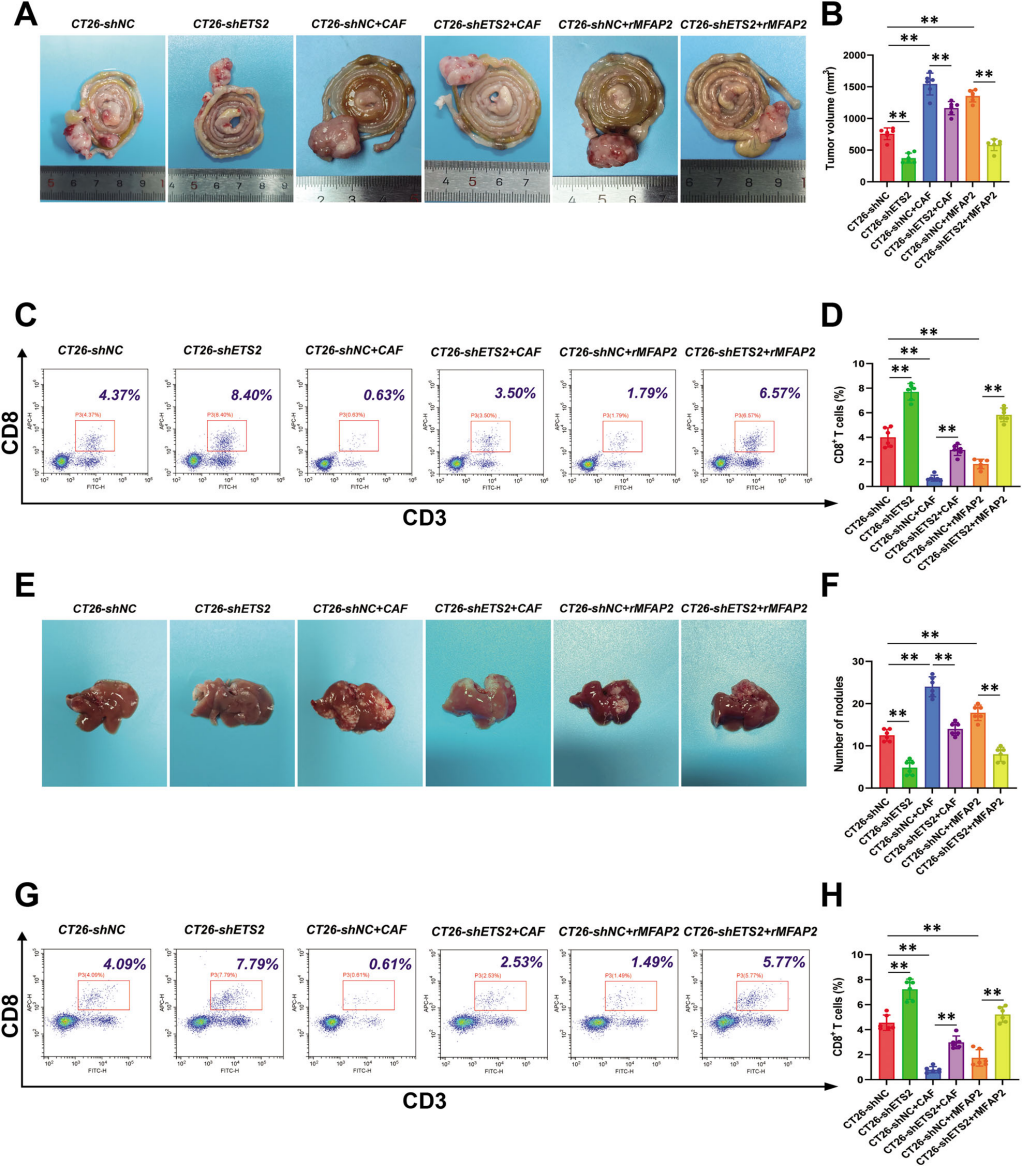
MFAP2 promotes CRC progression through ETS2 in vivo. **A** Gross appearance of colorectal tumors using the Swiss roll technique and quantification of tumor volume. **B** Quantitative analysis of orthotopic intestinal tumor volumes. **C** Representative flow cytometry plots assessing the proportion of CD3⁺CD8⁺ double-positive T cells in orthotopic intestinal tumor tissues. **D** Quantitative analysis of CD3⁺CD8⁺ double-positive T cells in orthotopic intestinal tumor tissues by flow cytometry. **E** Representative images of gross pathology for liver metastases from CRC. **F** Quantitative analysis of the number of liver metastasis nodules. **G** Representative flow cytometry data assessing the proportion of CD3⁺CD8⁺ double-positive T cells in liver metastasis tissues. **H** Quantitative analysis of CD3⁺CD8⁺ double-positive T cells in liver metastasis tissues. Data are shown as mean ± SD, *n* = 6, **P < 0.01.

To further validate the role of MFAP2 in regulating CRC through ETS2 in vivo, we established a syngeneic CRC model using CT26 cells. As shown in Fig. 7A, B, compared to the group injected with CT26 cells alone, the combination of CT26 cells and CAFs significantly promoted CRC growth (*P* < 0.01). However, after transfecting CT26 cells with shETS2, the tumor growth-promoting effect induced by CAF co-injection was significantly attenuated (*P* < 0.01). In addition, compared to the group injected with CT26 cells alone, the addition of rMFAP2 significantly promoted tumor growth (*P* < 0.01), while ETS2 knockdown reversed this effect (*P* < 0.01). Next, flow cytometry was used to assess changes in (CD3^+^CD8^+^ T cells) infiltration within tumor tissues (Fig. 7C-D). Co-injection of CAFs or rMFAP2 led to a decrease in the proportion of CD3^+^CD8^+^ T cells in tumor tissues, compared with the CT26 alone group (*P* < 0.01). However, ETS2 knockdown reversed CAFs or rMFAP2-mediated immune suppression, evidenced by significantly increased CD3^+^CD8^+^ T cell rates (*P* < 0.01).

Meanwhile, a murine model of CRLM was established to further evaluate the in vivo relevance of ETS2. As shown in Fig. 7E, F, compared to mice injected with CT26 cells alone, co-injection with CAFs or administration of rMFAP2 markedly enhanced hepatic metastatic burden (*P* < 0.01). Notably, this pro-metastatic effect was significantly attenuated upon ETS2 knockdown in CT26 cells (*P* < 0.01). To assess the immunological impact of ETS2, we further analyzed CD3⁺CD8⁺ T cell infiltration within liver metastases by flow cytometry (Fig. 7G, H). Both CAFs and rMFAP2 led to a significant reduction in intratumoral CD3^+^CD8⁺ T cell proportions (*P* < 0.01), suggesting an immunosuppressive microenvironment. Similarly, ETS2 silencing in tumor cells effectively reversed this suppression, restoring CD3⁺CD8⁺ T cell infiltration (*P* < 0.01). Collectively, these results demonstrate that CAFs-derived MFAP2 promotes CRC metastasis and inhibits CD8⁺ T cell-mediated immune surveillance in an ETS2-dependent manner in vivo.

## Discussion

The TME is a dynamic ecosystem that profoundly influences cancer development, progression, and metastasis. Among its cellular components, CAFs play a pivotal role in orchestrating tumor behavior through secreted factors, ECM remodeling, and immune modulation [10]. In this study, we identified MFAP2 as a significantly upregulated protein in CRC tissues, with enrichment in CAFs, and demonstrated its association with poor prognosis in CRC patients. These findings, validated through the TCGA database, underscore MFAP2 as a potential biomarker in CRC and highlight its role in CAF-CRC crosstalk within the TME. Our mechanistic investigations revealed that MFAP2 exerts immunosuppressive effects via the ITGB8 integrin receptor on CRC cells, activating the FAK–ERK1/2–ETS2–CYP27A1–LXRβ signaling axis, which promotes sterol secretion and CD8^+^ T cell exhaustion. These results provide novel insights into the molecular mechanisms underlying CAF-mediated immune evasion and tumor progression in CRC, offering potential therapeutic targets for immunotherapy.

Our study builds on the growing body of evidence that CAFs shape the TME through secreted proteins and signaling molecules [53]. MFAP2, a component of extracellular elastic microfibrils, has been implicated in EMT and tumor progression across various cancers. MFAP2 has emerged as a critical oncogenic factor across multiple cancer types, contributing to tumor progression through diverse mechanisms. In ovarian cancer, MFAP2 promotes malignancy by activating the FOXM1/β-catenin signaling axis, thereby enhancing aerobic glycolysis (the Warburg effect) [23]. This metabolic reprogramming is accompanied by increased glucose uptake, lactate production, and tumor growth, and MFAP2 overexpression is correlated with poor patient prognosis. Similarly, in HCC, MFAP2 facilitates tumor cell proliferation, with elevated expression levels being significantly associated with reduced overall survival [25]. Functional studies have further demonstrated that MFAP2 knockdown suppresses tumor growth and is positively correlated with regulatory T cells (Tregs) and immune checkpoints such as CTLA4 and TIGIT, highlighting its potential as a prognostic biomarker and therapeutic target. In gastric cancer, MFAP2 expression is markedly upregulated in tumor tissues and correlates with advanced stage and poor prognosis [54]. Mechanistically, it activates the PI3K/AKT signaling pathway to promote cell proliferation, invasion, and migration [55]. In osteosarcoma, MFAP2 drives EMT via activation of Notch1 signaling, enhancing migratory and invasive capabilities [56]. In esophageal squamous cell carcinoma (ESCC), MFAP2 contributes to tumor aggressiveness by upregulating COX-2, with overexpression linked to increased proliferation, invasion, and worse clinical outcomes [57]. Similarly, in oral squamous cell carcinoma (OSCC), MFAP2 promotes tumor progression by enhancing autophagy-dependent activation of the Wnt/β-catenin pathway, leading to elevated autophagic flux, proliferation, and invasion [58]. Beyond its roles in tumor cells, MFAP2 also marks a distinct subset of CAFs in gastric cancer that exhibit immunosuppressive properties and contribute to immune evasion [59]. These MFAP2⁺ CAFs are associated with resistance to both immunotherapy and chemotherapy, shaping an unfavorable TME and portending poor clinical outcomes. Together, these findings underscore the multifaceted oncogenic roles of MFAP2 and its significance in tumor metabolism, immune modulation, and therapeutic resistance across cancers. In CRC, our findings further extend the oncogenic role of MFAP2 by demonstrating that it is secreted by CAFs and activates the FAK-ERK1/2 signaling pathway via ITGB8, culminating in ETS2-dependent upregulation of the cholesterol-metabolizing enzyme CYP27A1. Elevated CYP27A1 expression promotes sterol secretion, which in turn activates liver X receptor β (LXRβ) signaling and drives CD8⁺ T cell exhaustion. These findings highlight a novel CAF-mediated metabolic–immunological axis orchestrated by MFAP2, reinforcing its role in shaping a tumor-permissive microenvironment and contributing to immune evasion and therapeutic resistance in CRC.

Integrin signaling plays a pivotal role in tumor progression by mediating cell–ECM interactions and transducing bidirectional signals that regulate diverse cellular processes, including proliferation, survival, migration, invasion, and therapeutic resistance. Dysregulated expression of integrins, particularly β1, β3, and αV subunits, has been observed across multiple cancer types and is associated with enhanced activation of downstream signaling pathways such as FAK/SRC, PI3K/AKT, and MAPK/ERK, which contribute to tumor cell growth and survival. Moreover, integrin signaling facilitates EMT, cytoskeletal remodeling, and MMP expression, thereby promoting invasiveness and metastasis [60–62]. In the TME, integrins are essential in shaping immunosuppressive niches by interacting with TGF-β and promoting the activation of CAFs and ECM remodeling [63, 64]. Given their central role in integrating intracellular signals and microenvironmental cues, integrins have emerged as promising therapeutic targets.

Given its established association with the ECM, MFAP2 is likely to interact with integrin receptors, leading to the activation of downstream kinases such as FAK and ERK1/2, and ultimately enhancing the transcriptional activity of ETS2 [65]. In our study, treatment with rMFAP2 resulted in the activation of the FAK–ERK1/2-ETS2 axis, which was accompanied by increased expression of CYP27A1, a mitochondrial enzyme involved in cholesterol metabolism, which catalyzes the hydroxylation of cholesterol at the C27 position, producing 27-HC. Cholesterol upregulates the expression of inhibitory immune checkpoint receptors such as PD-1, TIM-3, and LAG-3 on CD8^+^ T cells, and enhances the transcriptional activity of stress-responsive transcription factors, which drives exhaustion-related gene programs. Importantly, reducing cholesterol levels or disrupting cholesterol metabolism in T cells reverses the exhausted phenotype and restores their anti-tumor activity. This study established a mechanistic link between integrin signaling and the production of 27-HC, an immunosuppressive oxysterol known to induce CD8^+^ T cell exhaustion within the TME.

Despite the insights provided by our study, several limitations should also be taken into consideration. We acknowledged that other signaling pathways that influence EMT were not exhaustively examined in the present work. As shown in Fig. S20, there was a significant increase in phosphorylated Smad2 and Smad3 upon rMFAP2 treatment in both HT29 and SW620 cell lines (*P* < 0.01) compared with the tumor cell line alone group and albumin (ALB) control group, suggesting that TGF/Smad signaling might be partially involved. To comprehensively address this issue, future research should incorporate multi-omics approaches to conduct a thorough and extensive exploration of other pathways that may influence the biological role of MFAP2. While our research elucidates the pivotal role of MFAP2 secreted by CAFs in promoting CRC progression and immune evasion, it is critical to acknowledge that immune cell activity can also be autonomously regulated or directly modulated by CRC cells during tumor progression [10]. Specifically, CRC cells may orchestrate immune suppression through mechanisms independent of CAFs, such as the secretion of immunosuppressive cytokines or direct interactions with immune cells [64]. As shown in Fig. S21, MFAP2 knockdown in CAFs significantly increased the proportion of Granzyme B⁺IFN-γ⁺ CD8⁺ T cells and reduced their apoptosis (in the CAFs-CD8^+^ T cell system), indicating enhanced cytotoxicity. Supplementation with rMFAP2 partially reversed these effects, suggesting that CAFs-derived MFAP2 suppresses CD8⁺ T cell function and promotes apoptosis, thereby contributing to an immunosuppressive tumor microenvironment. These findings highlight a direct regulatory role of CAFs on CD8⁺ T cells, which should be fully considered in future studies. Meanwhile, as the integrin family comprises both α and β subunits, our study primarily focuses on the interaction between MFAP2 and the β subunit. The specific roles and potential synergistic effects of the α subunit remain underexplored.

Collectively, our study unveils a novel mechanism whereby MFAP2, secreted by CAFs, activates the ITGB8–FAK–ERK1/2–ETS2–CYP27A1–LXRβ signaling pathway, leading to the suppression of CD8^+^ T cell function and promoting malignant tumor progression in CRC. These findings provide critical insights into the role of CAFs in modulating anti-tumor immunity and highlight promising new avenues for developing more effective immunotherapeutic strategies for CRC.

## Supplementary information

Supplementary materials

Original Raw Data Western blots

## Data Availability

All data supporting this study are presented in this article and in its Supplementary materials.

## Code availability

The codes employed for the bioinformatics analyses in this paper are not publicly available due to data security considerations, but can be obtained from the corresponding author upon reasonable request.
