# Supplementary material for: Cancer-associated fibroblasts (CAFs) derived from MFAP2 promote CRC proliferation and metastasis while suppressing CD8+ T cell-mediated antitumor immunity
Source: Cell Death Dis. 2026 Jan 30;17(1):159. doi: 10.1038/s41419-026-08413-w (PMC12877200; doi:10.1038/s41419-026-08413-w)
Supplement: Supplementary file 1 — Supplementary materials [file 41419_2026_8413_MOESM1_ESM.pdf]

Supplementary Materials

Cancer-associated fibroblasts (CAFs) derived MFAP2 promotes CRC proliferation and metastasis while suppressing CD8$^+$ T cell-mediated anti-tumor immunity

Xu Zhang[1, #], Yuxiang Fei[3, #, *], Chunqi Xie[2, #], Tao Li[4], Ben Niu[2], Zihao Yang[2], Mengwei Song[2], Fanjun Meng[5], Hongting Diao[1, *], Jing Ji[1, 2, *], Qianming Du[4, 5, *], Chao Liu[3, 5, *]

[1] Department of Pharmacy, Chengdu Integrated TCM & Western Medicine Hospital, Chengdu University of TCM, 18# Wanxiang East Road, Chengdu, 610041, P.R. China; School of Nursing, Nanjing Medical University, Nanjing, 211168, P.R. China; College of Pharmacy, Taizhou University, Taizhou, 225300, P.R. China.

[2] Jiangsu Key Laboratory of Marine Pharmaceutical Compound Screening, College of Pharmacy, Jiangsu Ocean University, Lianyungang, 222001, P.R. China;

[3] Department of Pharmacy, Nanjing First Hospital, Nanjing Medical University, Nanjing, 210006, P.R. China.

[4] General Clinical Research Center, Nanjing First Hospital, Nanjing Medical University, Nanjing, 210006, P.R. China.

[5] School of Basic Medicine and Clinical Pharmacy, China Pharmaceutical University, Nanjing, 211198, P.R. China.

[#] These authors contributed equally for this article.

[*] To whom correspondence should be addressed.

Chao Liu, Department of Pharmacy, Nanjing First Hospital, Nanjing Medical University, Nanjing 210029, P.R. China. Email: liuchaogermany@126.com; Qianming Du, General Clinical Research Center, Nanjing First Hospital, Nanjing Medical University, Nanjing 210029, P.R. China. Email: duqianming@njmu.edu.cn; Jing Ji, Jiangsu Key Laboratory of Marine Pharmaceutical Compound Screening, College of Pharmacy, Jiangsu Ocean University, Lianyungang, 222005, P.R. China; College of Pharmacy, Taizhou University, Taizhou, Jiangsu 225300, P.R. China. Email: jijing@jou.edu.cn; Hongting Diao, School of Nursing, Nanjing Medical University, Nanjing, 211166, P.R. China. Email: dht@njmu.edu.cn; Yuxiang Fei, Department of Pharmacy, Nanjing First Hospital, Nanjing Medical University, Nanjing 210029, P.R. China. Email: fei_yu_xiang@163.com.

Supplementary Figures

1. Identification of primary CAFs from human and mouse source

To verify the identity and activation status of isolated fibroblast populations, we performed immunofluorescence staining for Vimentin and α-SMA in both human and mouse-derived fibroblasts. As shown in Fig.S1, both normal fibroblasts (NFs) and cancer-associated fibroblasts (CAFs) exhibited positive staining for Vimentin, confirming their mesenchymal origin. Notably, α-SMA expression was markedly upregulated in CAFs compared to NFs in both human and mouse samples, indicating a more activated phenotype typical of tumor-associated fibroblasts. These findings validate the successful isolation and characterization of NFs and CAFs across species.

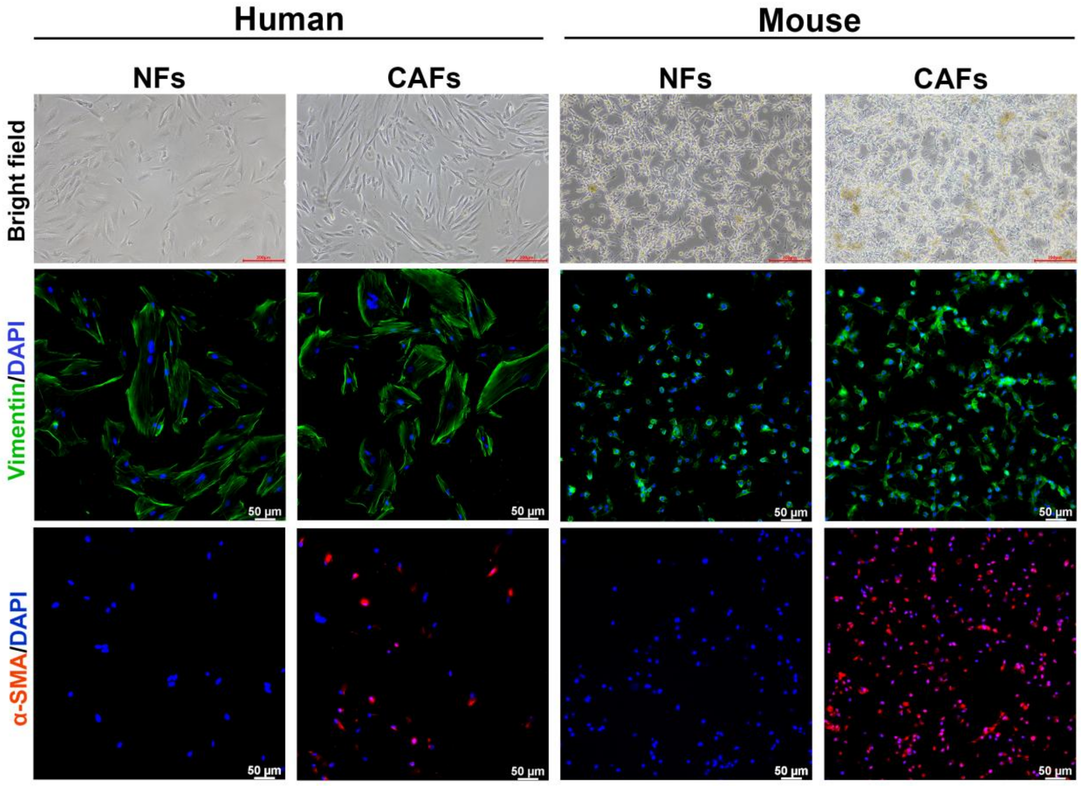

Fig.S1 Characterization of normal fibroblasts (NFs) and cancer-associated fibroblasts (CAFs) derived from human and mouse sources. The first row shows bright-field images illustrating the morphological characteristics of NFs and CAFs, Scale bar=200 μm. The second row presents immunofluorescence staining for Vimentin (green), a general fibroblast marker, Scale bar=50 μm. The third row shows α-SMA (red) expression, indicating fibroblast activation, which is markedly higher in CAFs than in NFs. Nuclei are counterstained with DAPI (blue), Scale bar=50 μm.

2. Distribution of MFAP2 expression in CAFs subpopulation

Moreover, we also performed subtype annotation of CAFs based on established marker genes corresponding to distinct CAF subsets. This analysis revealed that CAFs within the included clinical tumor samples could be classified into myCAF, matCAF,

iCAF, apCAF, proCAF, metabolic CAF, and normal fibroblasts (NF) populations (Fig.S2A). As can be seen in Fig.S2B, when MFAP2-positive CAFs were highlighted on the UMAP projection, matrix CAFs contributed predominantly to the overall MFAP2 expression although MFAP2 expression was observed across various CAF subpopulations.

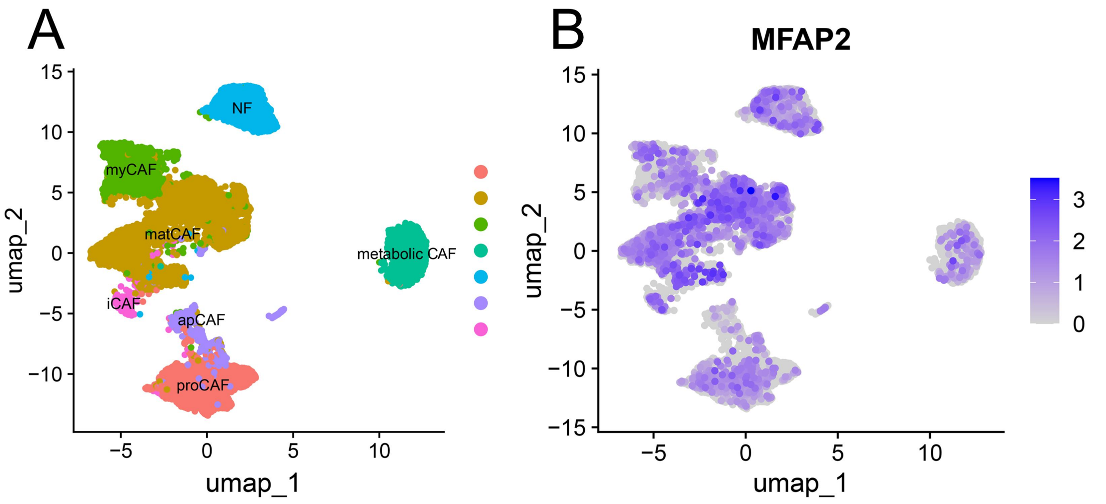

Fig.S2 MFAP2 expression in CAFs subpopulation. (A) The UMAP dimensionality reduction highlights distinct fibroblast subpopulations including myCAF, matCAF, iCAF, apCAF, proCAF, metabolic CAF, and normal fibroblasts (NF), based on canonical marker expression. (B) Expression distribution of MFAP2 across all fibroblast subpopulations shown in UMAP space.

3. MFAP2 derived from CAFs promotes proliferation and invasion in CRC

To investigate the functional role of MFAP2 derived from CAFs in CRC progression, we co-cultured HT29 cells with CAFs transduced with shRNA targeting MFAP2 (CAF-shMFAP2) or a non-targeting control (CAF-shNC). Western blot analysis revealed that CAF-shMFAP2 significantly increased the epithelial marker E-cadherin and decreased the mesenchymal markers N-cadherin, Snail, and Vimentin in HT29 cells (Fig.S3A-B), suggesting that MFAP2 promotes EMT. These findings were further corroborated by immunofluorescence staining, which showed elevated E-cadherin and reduced N-cadherin expression in HT29 cells co-cultured with CAF-shMFAP2, as compared to the control group (Fig.S3C-D, $P<0.01$).

To evaluate whether CAFs-derived MFAP2 influences the invasive capacity of CRC cells under the pressure of activated $CD8^+$ T cells, transwell invasion assays were conducted using HT29 and SW620 cell lines. As shown in Fig.S3E-G, silencing MFAP2 in CAFs (CAF-shMFAP2) significantly reduced the invasiveness of both cell lines ($P<0.01$). Notably, introduction of rMFAP2 into the co-culture system (CAF-shMFAP2+rMFAP2) partially restored the invasive phenotype ($P<0.01$).

Finally, we examined the impact of CAFs-derived MFAP2 on tumor cell viability in the presence of $CD8^+$ T cells. Co-culture of CAF-shMFAP2 with $CD8^+$ T cells significantly reduced the viability of HT29 and SW620 cells ($P<0.01$), whereas

addition of MFAP2 in CAFs reversed this reduction (Fig.S3H-I, P<0.01). These data suggest that CAFs-derived MFAP2 promotes tumor cell proliferation and invasion, and may also impair CD8^{+} T cell-mediated cytotoxicity.

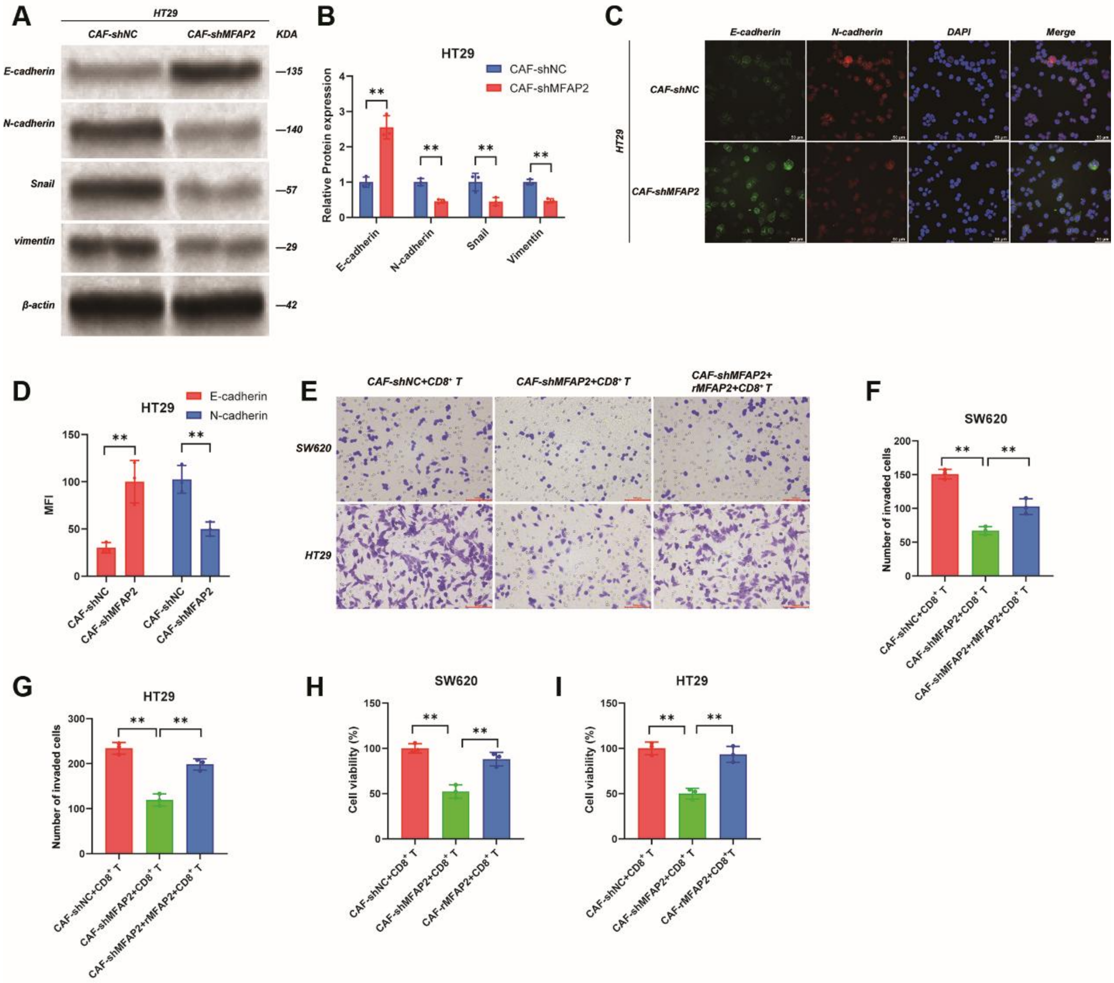

Fig.S3 CAFs-derived MFAP2 promotes epithelial-mesenchymal transition, invasion, and proliferation of CRC cells. (A) Western blot analysis of E-cadherin, N-cadherin, Vimentin, and Snail protein levels in HT29 cells after co-culture with CAFs under different conditions. (B) Quantification of protein expression in panel (A), normalized to β-actin. (C) Representative immunofluorescence images showing E-cadherin and N-cadherin expression in HT29 cells co-cultured with CAF-shNC or CAF-shMFAP2. (D) Quantification of mean fluorescence intensity (MFI) of E-cadherin and N-cadherin from panel (C). (E) Representative images of Transwell invasion assays for SW620 and HT29 cells following co-culture with CAFs under the indicated conditions. Quantification of invaded SW620 (F) and HT29 (G) cells. Cell viability of SW620 (H) and HT29 (I) after co-culture with CAF-shNC or CAF-shMFAP2 in the presence of CD8^{+} T cells were determined via CCK-8. Data are presented as mean ± SD, n=3, **P<0.01.

4. MFAP2 knockdown in CAFs alleviates CAFs-induced EMT phenotype in CRC cells

To verify the role of MFAP2 in CAF-induced EMT of CRC cells, we co-cultured

HT29 and SW620 cells with control CAFs, CAFs transduced with non-targeting shRNA (CAF-shNC), or MFAP2-knockdown CAFs (CAF-shMFAP2), followed by immunofluorescence staining for E-Cadherin and N-Cadherin. As shown in Fig.S4A and D, CRC cells co-cultured with CAFs or CAF-shNC exhibited decreased E-Cadherin and increased N-Cadherin expression ($P<0.01$). In contrast, co-culture with CAF-shMFAP2 partially reversed this phenotype, with elevated E-Cadherin and reduced N-Cadherin levels ($P<0.01$). Quantitative analysis of mean fluorescence intensity (MFI) confirmed these observations in both HT29 (Fig.S4B and C) and SW620 cells (Fig.S4E and F), suggesting that MFAP2 in CAFs is functionally involved in promoting EMT in CRC cells.

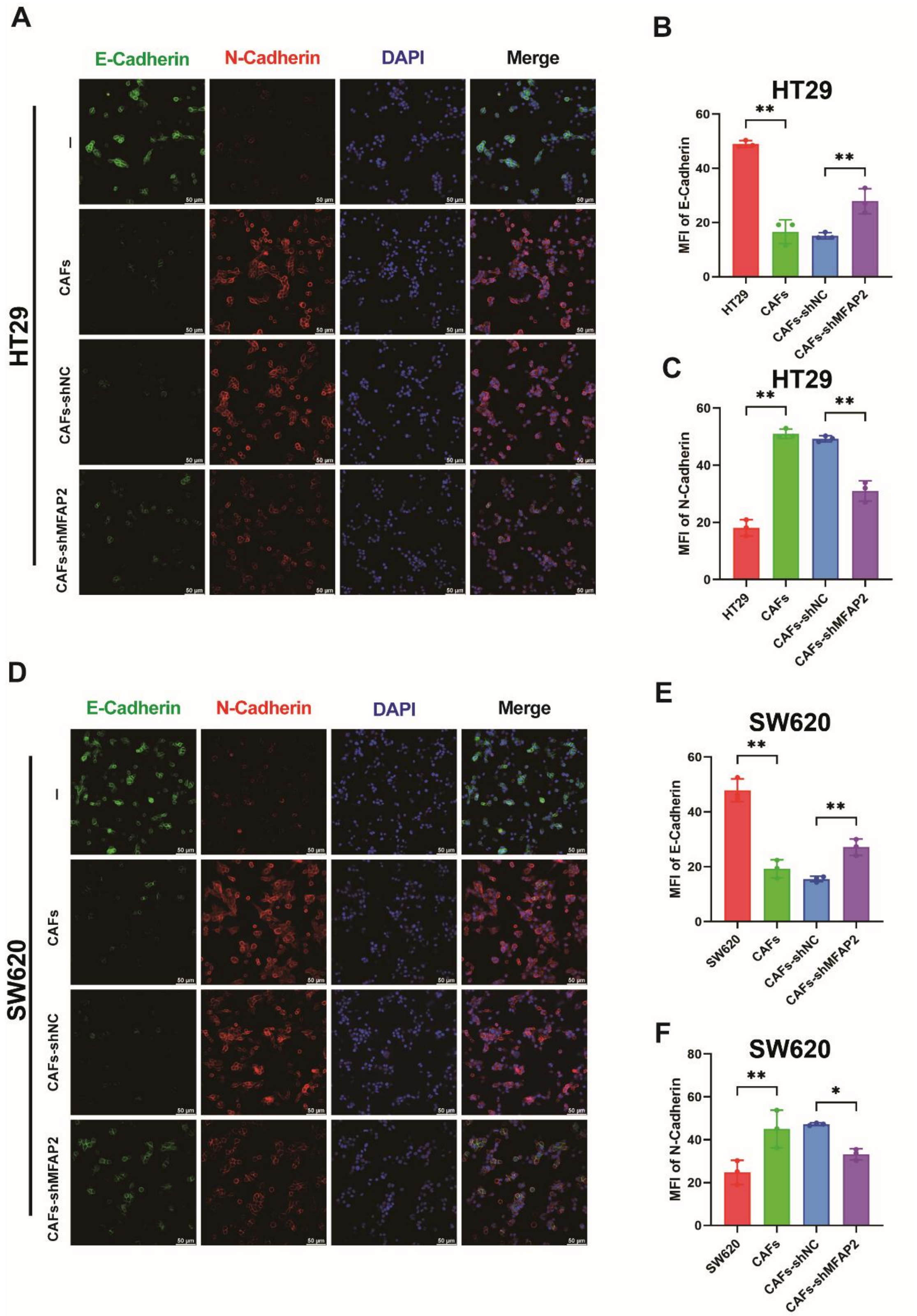

Fig.S4 MFAP2 knockdown in CAFs alleviates CAFs-induced EMT phenotype in CRC cells. Representative immunofluorescence images showing the expression of E-Cadherin (green), N-Cadherin (red) in HT29 (A) and SW620 (D) cells after co-culture with CAFs. Scale bar=50 μm. Quantification of E-Cadherin mean fluorescence intensity (MFI) in HT29 (B) and SW620 (E) cells under different

co-culture conditions. Quantification of N-Cadherin MFI in HT29 (C) and SW620 (F) cells. Data are presented as mean ± SD, n=3. *P<0.05, **P<0.01.

5. Knockdown of MFAP2 in CAFs suppresses EMT-related protein expression in CRC cells

To further validate the role of CAFs-derived MFAP2 in promoting EMT in CRC cells, we examined the expression of EMT-related proteins in HT29 and SW620 cells after co-culture with CAFs, CAF-shNC, or CAF-shMFAP2. As shown in the Western blot results (Fig.S5A and C), co-culture with CAFs or CAF-shNC markedly reduced E-Cadherin expression while increasing the expression of mesenchymal markers including N-Cadherin, Snail, and Vimentin, compared to HT29 and SW620 cells alone. Notably, knockdown of MFAP2 in CAFs significantly reversed these effects, as indicated by elevated E-Cadherin levels and decreased expression of N-Cadherin, Snail, and Vimentin.

Quantitative analysis of the band intensities (Fig.S5B and D) confirmed the statistical significance of these changes (P<0.05 and P<0.01), indicating that MFAP2 is required for the full induction of EMT-related protein expression in CRC cells mediated by CAFs.

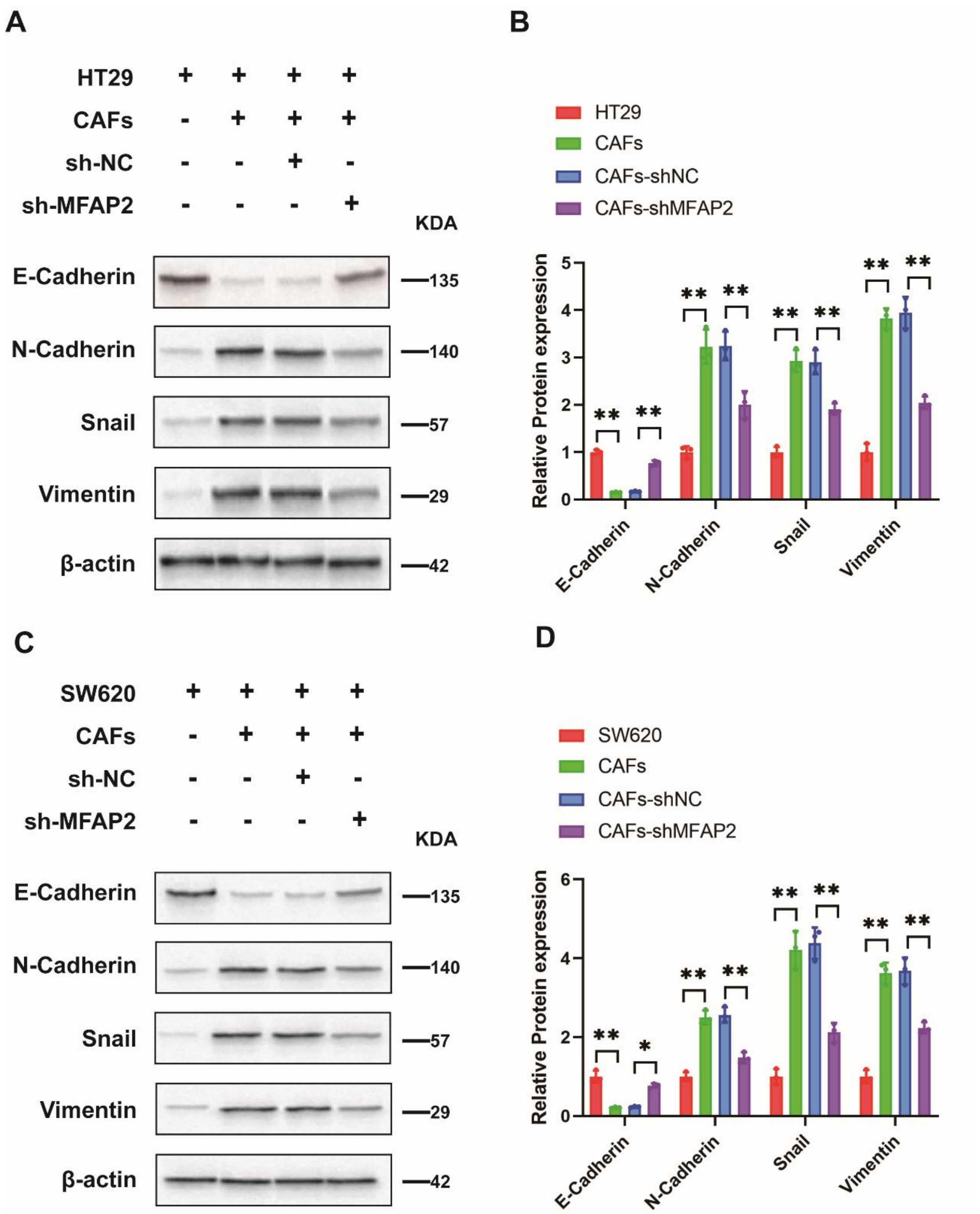

Fig.S5 Knockdown of MFAP2 in CAFs suppresses EMT-related protein expression in CRC cells. Representative Western blot analysis of EMT markers (E-Cadherin, N-Cadherin, Snail, and Vimentin) in HT29 (A) and SW620 (C) cells after co-culture with CAFs (β-actin was introduced as loading control). Quantitative analysis of the relative expression levels of EMT-related proteins in HT29 (B) and SW620 (D) cells. Data are shown as mean ± SD, n=3. **P*<0.05, ***P*<0.01.

6. Elevated secretion of MFAP2 by CAFs enhances CRC cell invasiveness

To explore the source and functional relevance of MFAP2 in the tumor microenvironment, we first assessed MFAP2 secretion levels in conditioned media

(CM) from CRC cell lines (SW620 and HT29) and CAFs. ELISA results revealed that CAFs secreted significantly higher levels of MFAP2 compared to both CRC cell lines ($P<0.001$, Fig.S6A), suggesting that CAFs are a major source of MFAP2 within the tumor stroma.

To further investigate the functional role of CAFs-derived MFAP2 in promoting CRC cell invasion, neutralizing antibodies against MFAP2 were applied in a transwell invasion assay. As shown in Fig.S6B and quantified in Fig.S6C, blockade of MFAP2 in CAF-CM significantly reduced the invasive ability of both HT29 and SW620 cells compared to IgG controls ($P<0.01$), highlighting the pro-invasive role of CAF-secreted MFAP2 in CRC progression.

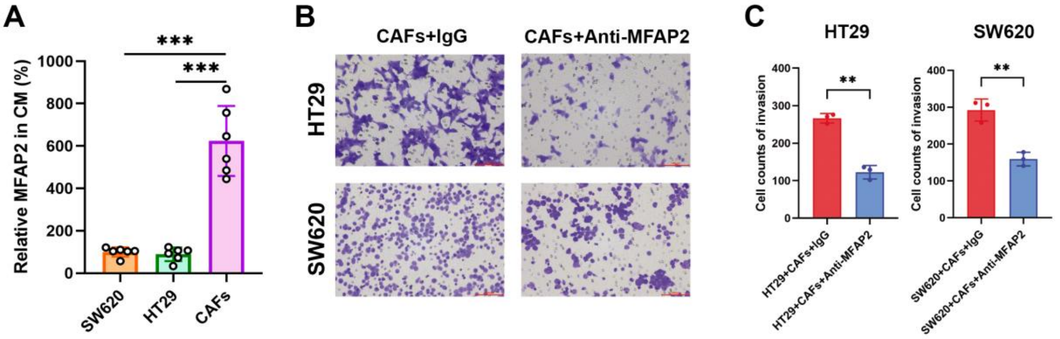

Fig.S6 CAFs-derived MFAP2 promotes CRC cell invasion. (A) ELISA analysis of MFAP2 levels in conditioned medium (CM) collected from SW620, HT29, and CAFs. (B) Representative images of transwell invasion assays of HT29 and SW620 cells co-cultured with CAFs in the presence of control IgG or MFAP2-neutralizing antibody. (C) Quantification of invasive cell numbers in HT29 and SW620 cell lines. Data are shown as mean ± SD, n=3 or 6, $^{**}P<0.01$, $^{***}P<0.001$.

7. MFAP2^{+}CAFs are associated with reduced CD8+ T cell infiltration in the tumor microenvironment

To investigate the relationship between MFAP2 expression in CAFs and CD8^{+} T cell infiltration in colorectal tumors, we analyzed single-cell RNA sequencing data from 15 clinical tumor samples. CAFs were stratified based on MFAP2 expression levels. Cells with MFAP2 expression above the median were defined as the MFAP2-high CAF group, while those below the median comprised the MFAP2-low CAF group.

As shown in Fig.S7A-F, UMAP-based dimensionality reduction plots and corresponding bar graphs revealed that tumor tissues enriched in MFAP2-high CAFs exhibited a significantly lower infiltration of CD8^{+} T cells compared to those with MFAP2-low CAFs. Furthermore, correlation analysis demonstrated a negative association between MFAP2 expression in CAFs and the abundance of CD8^{+} T cells within the tumor microenvironment (r=-0.407, Fig.S7G), suggesting that elevated MFAP2 expression may contribute to an immune-excluded phenotype by limiting cytotoxic T cell infiltration.

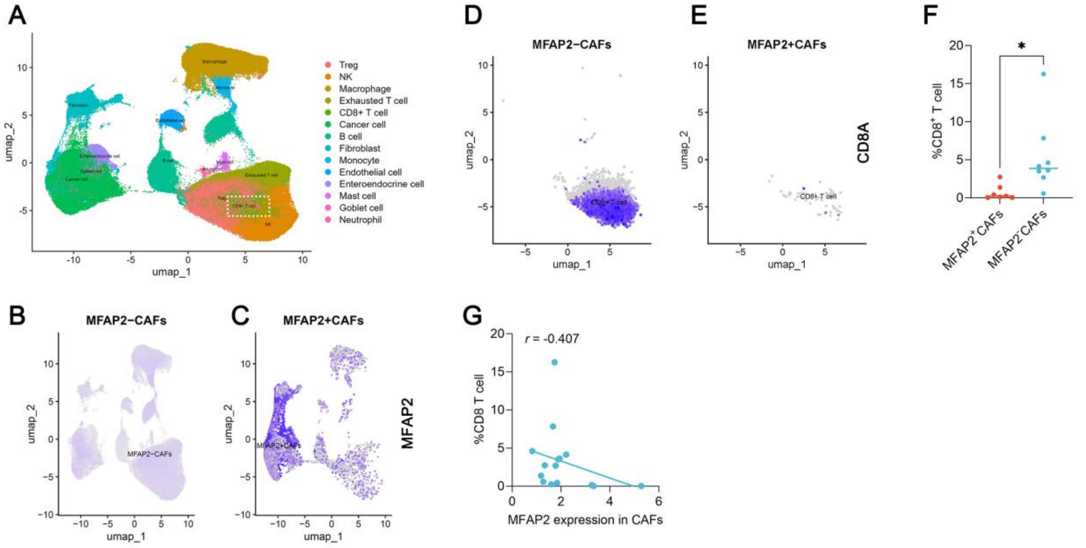

Fig.S7 MFAP2$^+$CAFs are associated with reduced CD8$^+$ T cell infiltration in the tumor microenvironment. (A) UMAP visualization of single-cell RNA sequencing data from tumor samples showing the major immune and stromal cell populations annotated by cell type. UMAP projection showing the distribution of MFAP2$^-$CAFs (B) and MFAP2$^+$CAFs (C). Abundance of CD8$^+$ T cells in MFAP2$^-$CAFs group (D) and MFAP2$^+$CAFs group (E). (F) Quantification of CD8$^+$ T cell infiltration in tumors. (G) Pearson correlation analysis between MFAP2 expression in CAFs and the proportion of CD8$^+$ T cells within tumors demonstrates a significant negative correlation (r=-0.407). Data are shown as mean ± SD, *P<0.05.

8. MFAP2 knockdown in CAFs suppresses liver metastasis and tumor growth in vivo

we have conducted additional in vivo experiments using nude mice to further validate the functional role of MFAP2$^+$CAFs in tumor progression after excluding the influence of immune cells. Both liver metastasis and subcutaneous tumor models were established using SW620 cells. As shown in Fig.S8A-B, in the liver metastasis model, co-injection of CAFs with SW620 cells markedly increased the number of visible liver nodules compared to SW620 cells alone (*P*<0.01). However, knockdown of MFAP2 in CAFs (CAFs-shMFAP2) significantly reduced metastatic burden (*P*<0.01), and this inhibitory effect was partially reversed by the addition of rMFAP2 (*P*<0.01). Consistently, in the subcutaneous tumor model (Fig.S8C-D), SW620 cells co-injected with CAFs-shNC exhibited enhanced tumor growth (*P*<0.01), whereas CAFs-shMFAP2 markedly suppressed tumor volume (*P*<0.01), which was significantly reversed by the administration of rMFAP2 rescued tumor growth to a level comparable to the control.

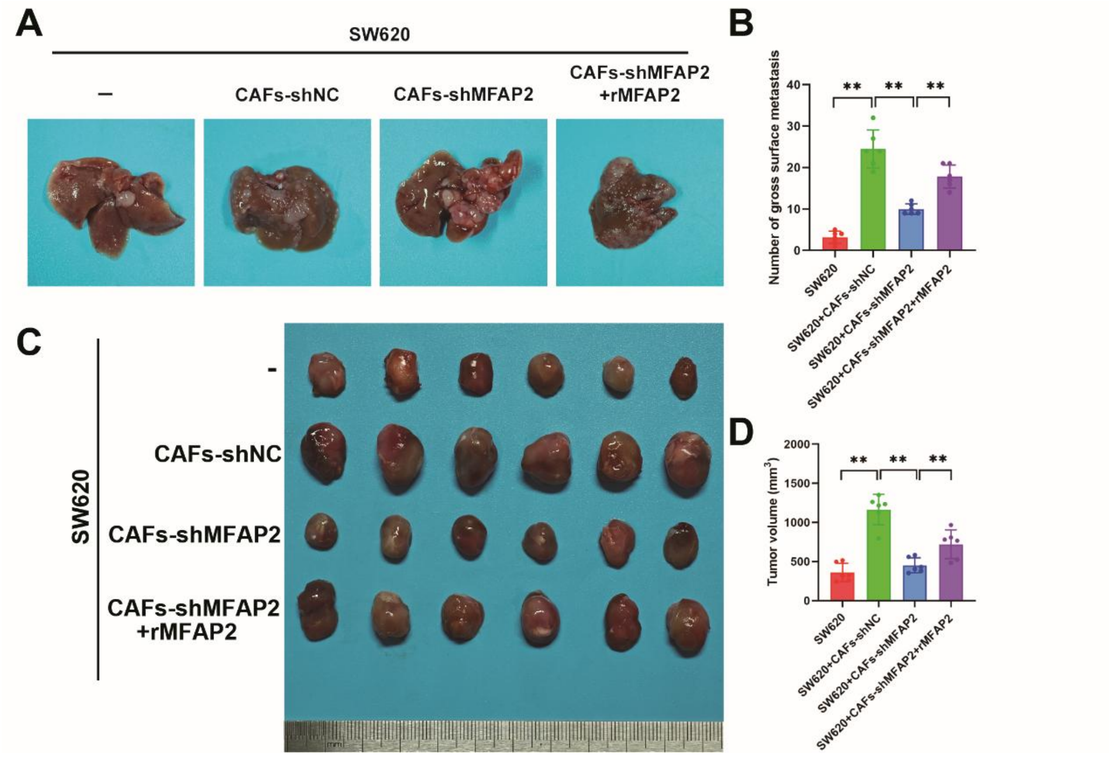

Fig.S8 MFAP2 knockdown in CAFs suppresses liver metastasis and tumor growth of SW620 cells in vivo. (A) Representative images of liver metastases in mice injected with SW620. (B) Quantification of the number of visible liver surface metastases. (C) Representative images of subcutaneous tumors harvested from mice injected with SW620 cells. (D) Quantification of subcutaneous tumor volume. Data are shown as mean ± SD, n=6, $^{**}P<0.01$.

9. Tumor volume dynamics reflect tumor cell abundance under CAF-Modified conditions

The differential proliferation rates of tumor cells and CAFs are a recognized phenomenon in co-culture and co-injection orthotopic tumor models. While this introduces complexity in interpreting tumor growth dynamics, our experimental design accounts for this by standardizing the initial cell ratios. To further address potential variability, we have now included a statistical analysis of the tumor cell/CAF ratio at the end time point in the revised manuscript. We believe these data adequately address the reviewer's concern while acknowledging the inherent challenges in fully controlling cell ratio dynamics in vivo, a limitation shared across similar studies in the field.

To investigate the tumor cell/CAF ratio, we performed immunofluorescence staining on the resulting orthotopic tumors. In the harvested orthotopic tumors, EPCAM and α-SMA were used to mark tumor cells and CAFs, respectively (Fig.S9A). Cell populations analysis revealed that in comparison with the CT26+CAFs-shRNA group, the tumor cell/CAF ratio of CT26+CAFs-shMFAP2 significantly decreased (Fig.S9B, $P<0.01$). While addition of rMFAP2 significantly increased the tumor cell/CAF ratio (Fig.S9B, $P<0.05$), reflecting differential proliferation rates between

tumor cells and CAFs under various CAF treatment conditions. Notably, the MFI of a-SMA remained unchanged, suggesting that tumor volume changes are primarily attributed to alterations in tumor cell abundance.

We acknowledge that tumor cells and CAFs proliferate at different rates in the co-injection system, which may influence the tumor microenvironment and growth dynamics. Importantly, the tumor volume measurements reported in Fig.S8 and Fig.S11 remain robust and reproducible, indicating that the observed tumor growth differences are driven by the CAF treatment conditions, despite the ratio variations.

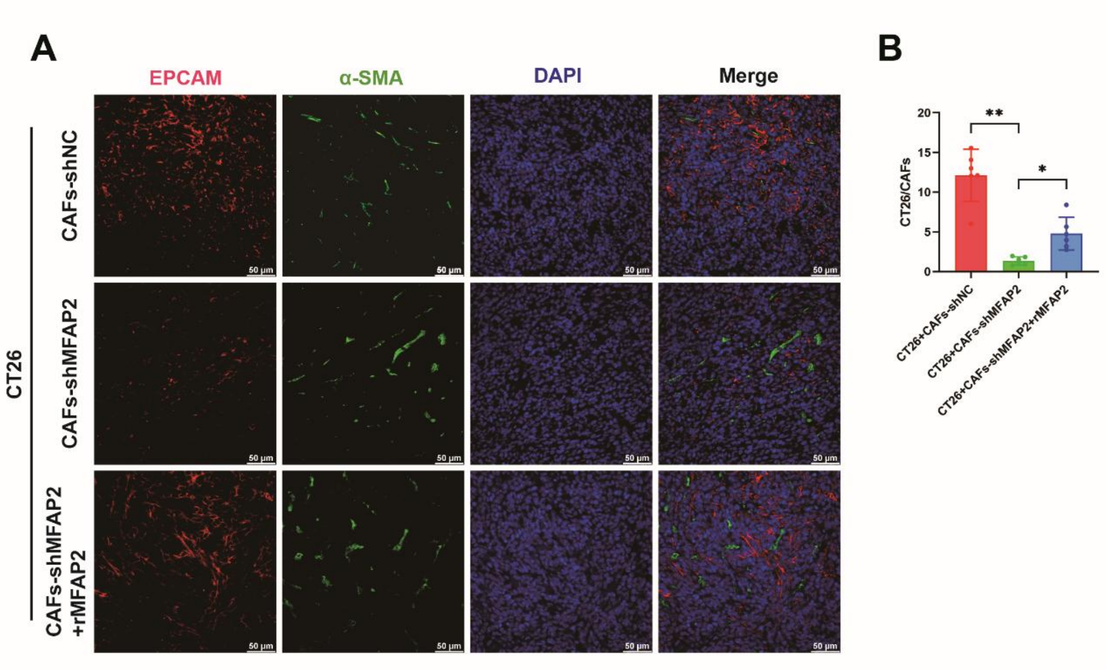

Fig.S9 Impact of CAFs-derived MFAP2 modulation on the expression of α-SMA and EPCAM in the resulting orthotopic CT26 tumors. (A) Representative immunofluorescence images showing the expression of EPCAM (red), α-SMA (green) in orthotopic CT26 tumors co-injection with CAFs under different conditions. Scale bar, 50 μm. (B) Quantification of the ratio of CT26 (EPCAM)/CAFs (α-SMA). Data are shown as mean ± SD, n=6, **P*<0.05, ***P*<0.01.

10. CAFs-derived MFAP2 decreased the CD8$^+$ T cell infiltration in tumor tissues.

As shown in Fig.S10A-B, immunohistochemistry showed reduced CD8$^+$ T cells throughout tumors when CAFs were co-injected with CT26 (P<0.01). While, after the knockdown of MFAP2, increased CD8$^+$ cells infiltration was observed (P<0.01), which was reversed by the introduction of rMFAP2 (P<0.05). Collectively, these data demonstrate that CAFs-secreted MFAP2 suppresses CD8$^+$ T-cell infiltration within the tumor microenvironment, suggesting that MFAP2 might promote tumor progression by attenuating anti-tumor immunity.

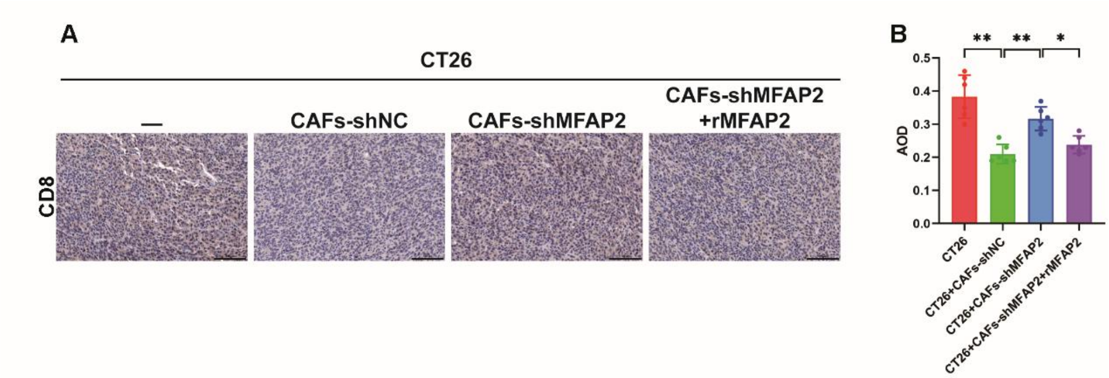

Fig.S10 CAFs-derived MFAP2 decreased the CD8^{+} T cell infiltration in tumor tissues. (A) Representative immunohistochemical staining images showing CD8^{+} T cell infiltration in tumor tissues. (B) Quantitative analysis of CD8 immunostaining. Data are shown as mean ± SD, n=6, **P*<0.05, ***P*<0.01.

11. MFAP2 promotes CRC growth and liver metastasis through a ETS2 dependent manner

To investigate the role of MFAP2 in promoting CRC progression in vivo, we established orthotopic implantation and liver metastasis models using CT26 cells co-injected with CAFs subjected to MFAP2 knockdown or rMFAP2 rescue. Representative bioluminescence images and quantification of total radiant efficiency revealed that co-injection with CAF-shNC significantly enhanced both orthotopic tumor growth and liver metastasis compared to CT26 cells alone (Fig.S11A-C, *P*<0.01). Notably, knockdown of MFAP2 in CAFs markedly suppressed tumor progression and metastasis (*P*<0.01), while supplementation with rMFAP2 did not restore tumor progression.

We further assessed whether ETS2 was involved in the MFAP2-mediated pro-tumorigenic function. Silencing ETS2 in CT26 cells abrogated the enhancement of liver metastasis and orthotopic tumor growth induced by CAFs or rMFAP2 (Fig.S11D-F, *P*<0.01). Quantitative analysis confirmed that both CAF- and rMFAP2-mediated promotion of tumor burden was dependent on ETS2 expression in tumor cells, as evidenced by significantly reduced bioluminescent signals in the shETS2 groups (Fig.S11D-F, *P*<0.01). These data demonstrate that CAFs-derived MFAP2 might promote CRC progression and metastasis via a CAF-tumor-ETS2 axis.

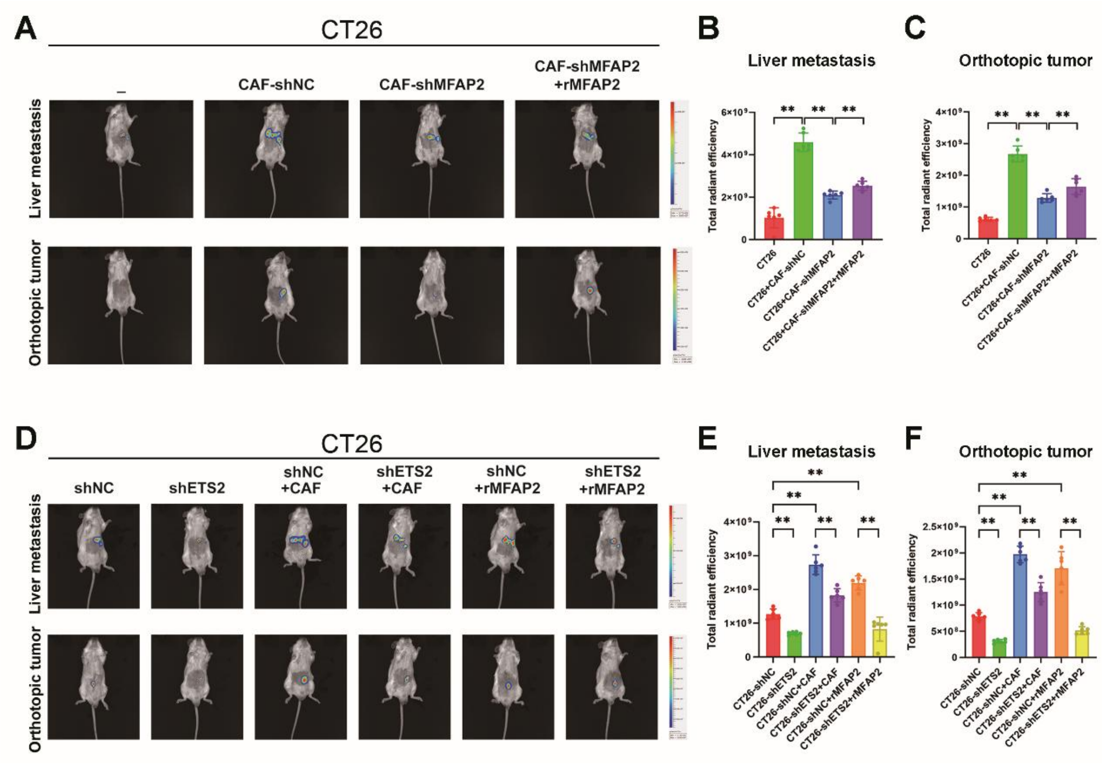

Fig.S11 MFAP2 promotes CRC growth and liver metastasis through a ETS2 dependent manner. Representative bioluminescence images (A and D) and quantification of tumor burden (B and E for liver metastasis model; C and F for orthotopic model). Data are presented as mean ± SD, n=6, ***P*<0.01.

12. rMFAP2 directly impairs CD8$^+$ T cell infiltration and promotes tumor growth in vivo

To address whether rMFAP2 alone exerts similar effects in CT26 tumor-bearing mice, we conducted in vivo experiments with the following groups: CT26, CT26+ALB (albumin control), and CT26+rMFAP2. Flow cytometry analysis revealed a significant decrease in tumor-infiltrating CD8$^+$ T cells in the CT26+rMFAP2 group compared to both the CT26 alone and CT26+ALB control groups (Fig.S12A-B, *P*<0.01). Consistently, mice receiving rMFAP2 developed markedly larger tumors, as shown by gross tumor morphology and increased tumor volume measurements (Fig.S12C-D, *P*<0.01). These findings suggest that MFAP2 also suppresses CD8$^+$ T cell infiltration into tumors and facilitates tumor progression in vivo.

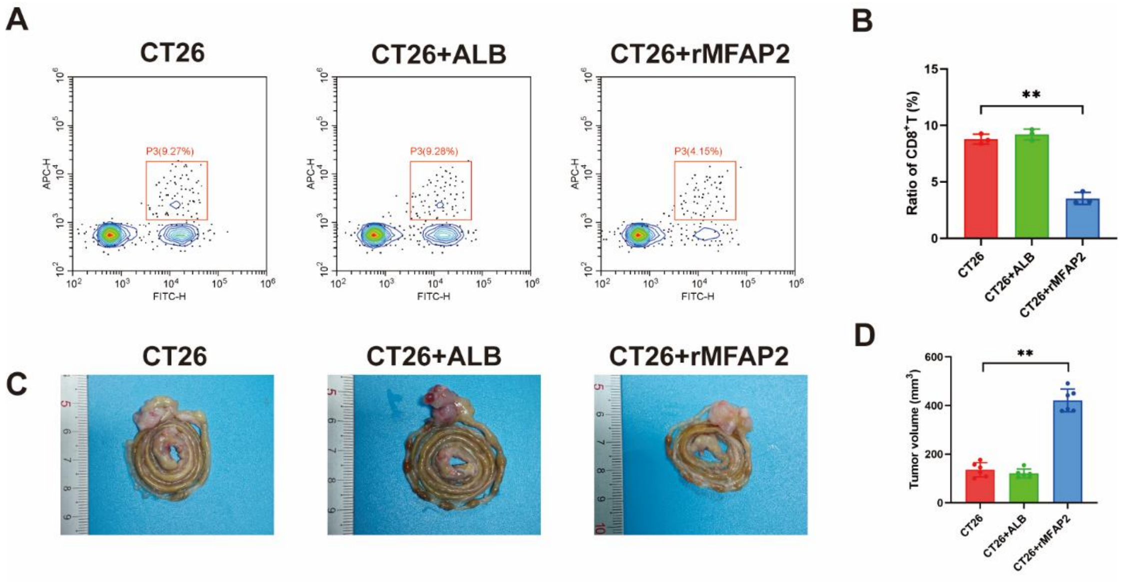

Fig.S12 rMFAP2 directly impairs CD8^{+} T cell infiltration and promotes tumor growth in vivo. (A) Representative flow cytometry plots showing the proportion of CD8^{+} T cells infiltrating tumors in different groups. (B) Quantification of CD8^{+} T cell infiltration in the three groups. (C) Representative images of orthotopic tumor from each group. (D) Tumor volumes were measured and compared among groups. Data are presented as mean ± SD, n=3 or 6, ***P*<0.01.

13. Detection of ITGB8 in the CRC clinical samples

As can be seen in the UMAP-based visualization, ITGB8 is predominantly expressed by cancer cells (Fig.S13A-B). Representative immunofluorescence staining of ITGB8 (red), pan-cytokeratin (PAN-CK, green), and DAPI (blue) in CRC tissues and adjacent normal tissues (NC). In CRC samples, ITGB8 and PAN-CK are markedly upregulated compared to NC, with strong co-localization observed in tumor epithelial regions (Fig.S13C). Quantification of the mean fluorescence intensity (MFI) of ITGB8 and PAN-CK showed both ITGB8 and PAN-CK exhibited significantly increased expression in CRC samples compared with (*P*<0.01) adjacent NC.

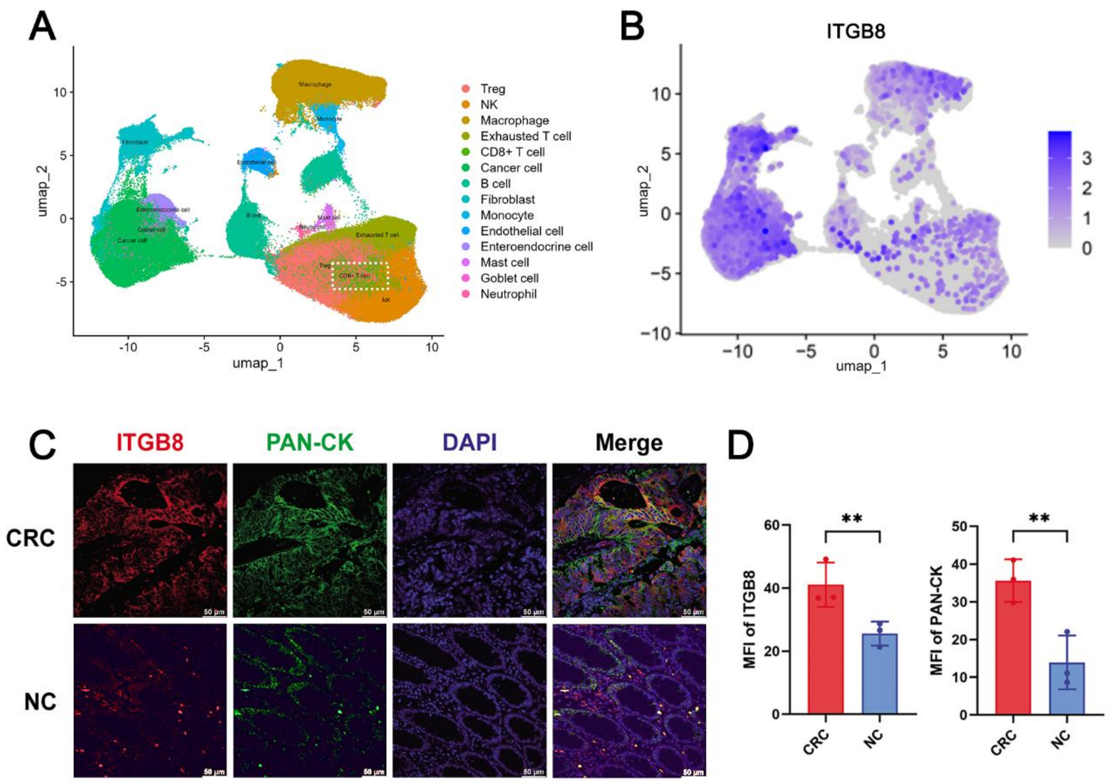

Fig.S13 Detection of ITGB8 in the CRC clinical samples. (A) UMAP embedding of integrated single-cell transcriptomes from CRC tissues, colored by major cell-type annotations. (B) ITGB8-expressing cells were highlighted. (C) Representative immunofluorescence images of ITGB8 (red), pan-cytokeratin (PAN-CK, green), and nuclei (DAPI, blue) in CRC and adjacent normal tissues. Scale bars, 50 µm. (D) Quantitative analysis of the mean fluorescence intensity (MFI) of ITGB8 and PAN-CK in CRC vs. NC tissues. Data are shown as mean ± SD, n=3, $P<0.01$.

14. Knockdown efficiency of target genes

To investigate the functional role of ITGB1-8, we first established stable ITGB1-8 knockdown cell lines in SW620 and HT29 cells using shRNA. Three distinct shRNA constructs were used to ensure the specificity of the knockdown. Western blot showed that relevant sequences effectively reduced the protein levels in both SW620 and HT29 cells (Fig.S14A-H).

Furthermore, we used immunofluorescence to confirm the knockdown efficiency of both ITGB8 and MFAP2, as well as their co-localization, in HT29 cells. The results showed a clear reduction in the fluorescence intensity of ITGB8 and MFAP2 in the respective knockdown groups, confirming the successful silencing of these genes at the protein level (Fig.S14I-J, $P<0.01$). These results provide a solid foundation for further investigations into the biological functions of ITGB8 and MFAP2.

To rule out any off-target effects of the shRNA that could directly impact cell proliferation, a CCK-8 cell viability assay was performed. As shown in Fig.S14K, knockdown of ITGB1-8 did not significantly alter the proliferation rates of either SW620 or HT29 cells, suggesting that knockdown of ITGB1-8 themselves does not directly regulate basal cell viability under standard culture conditions. This finding

validates our subsequent functional assays, ensuring that any observed effects are due to the specific role of ITGB8 rather than a general cytotoxic effect.

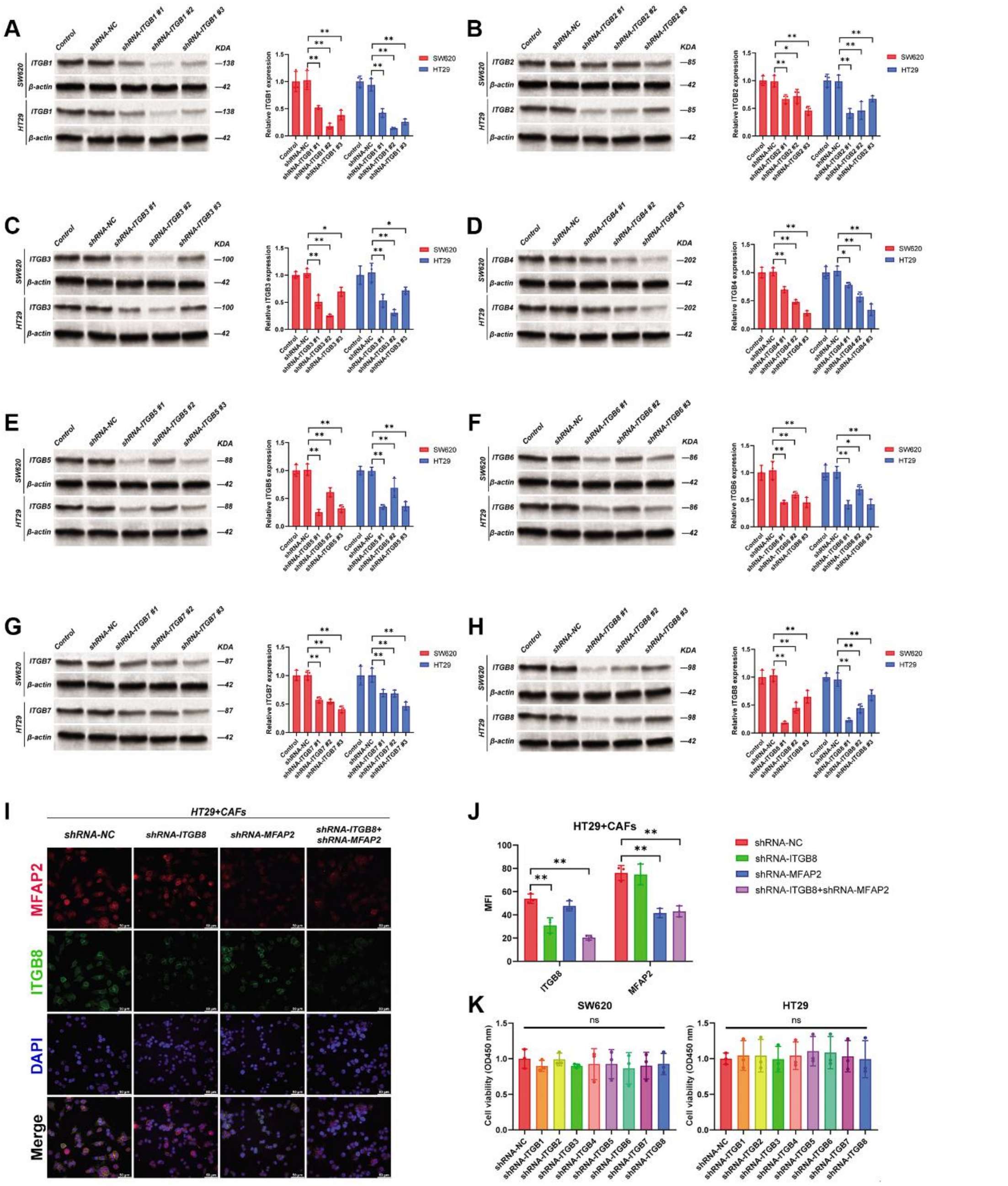

Fig.S14 Knockdown efficiency of target genes and their effects on cell viability. (A)-(H) Western blot analysis of ITGB1-8 protein levels in SW620 and HT29 cells after gene knockdown. Cells were transfected with different shRNA constructs targeting ITGB1-8. β-actin served as a loading control. The right panels show the quantification of relative protein expression levels. (I) Representative immunofluorescence staining of HT29 cells co-cultured with CAFs after knocking down ITGB8, MFAP2, or both. Scale bar, 50 μm. (J) Quantification of mean fluorescence intensity (MFI) of ITGB8 and MFAP2 in HT29 cells. (K) Cell viability assays using a CCK-8 kit to evaluate the direct effects of ITGB1-8 knockdown on SW620 and HT29 cells. Data are presented as mean ± SD, n=3, *P<0.05, **P<0.01.

15. MFAP2 promotes CRC progression through the ITGB8/FAK/ETS2 signaling pathway

As shown in Fig.S15A-D, to investigate the mechanism by which MFAP2 promotes CRC progression, we first assessed the downstream signaling pathways activated in HT29 cells upon co-culture with CAFs. Western blot analysis revealed that knockdown of MFAP2 in CAFs markedly reduced the phosphorylation levels of FAK and ERK1/2 (P<0.01), as well as the expression of ETS2 in HT29 cells (P<0.01), whereas treatment with rMFAP2 enhanced their expression (P<0.01).

Given the known interaction between integrin signaling and MFAP2, we next explored the role of ITGB8, a key integrin subunit. Knockdown of ITGB8 in HT29 cells resulted in significant decreases in p-FAK, p-ERK1/2, and ETS2 expression (P<0.01). Notably, supplementation with rMFAP2 failed to restore ETS2 levels in ITGB8-deficient cells (Fig.S15E-H), suggesting that MFAP2 requires ITGB8 to activate downstream ETS2 signaling.

Furthermore, as can be seen in Fig.S15I-L, overexpression of ETS2 in HT29 cells robustly increased its protein expression (P<0.01), whereas shRNA-mediated knockdown significantly suppressed ETS2 levels (P<0.01), confirming the effectiveness of ETS2 modulation, so as to enable further experimental investigations.

These results collectively indicate that CAFs-derived MFAP2 promotes ETS2 activation in CRC cells via an ITGB8-dependent FAK/ERK signaling axis, and that disruption of either MFAP2 in CAFs or ITGB8 in cancer cells impairs ETS2-mediated pro-tumorigenic signaling.

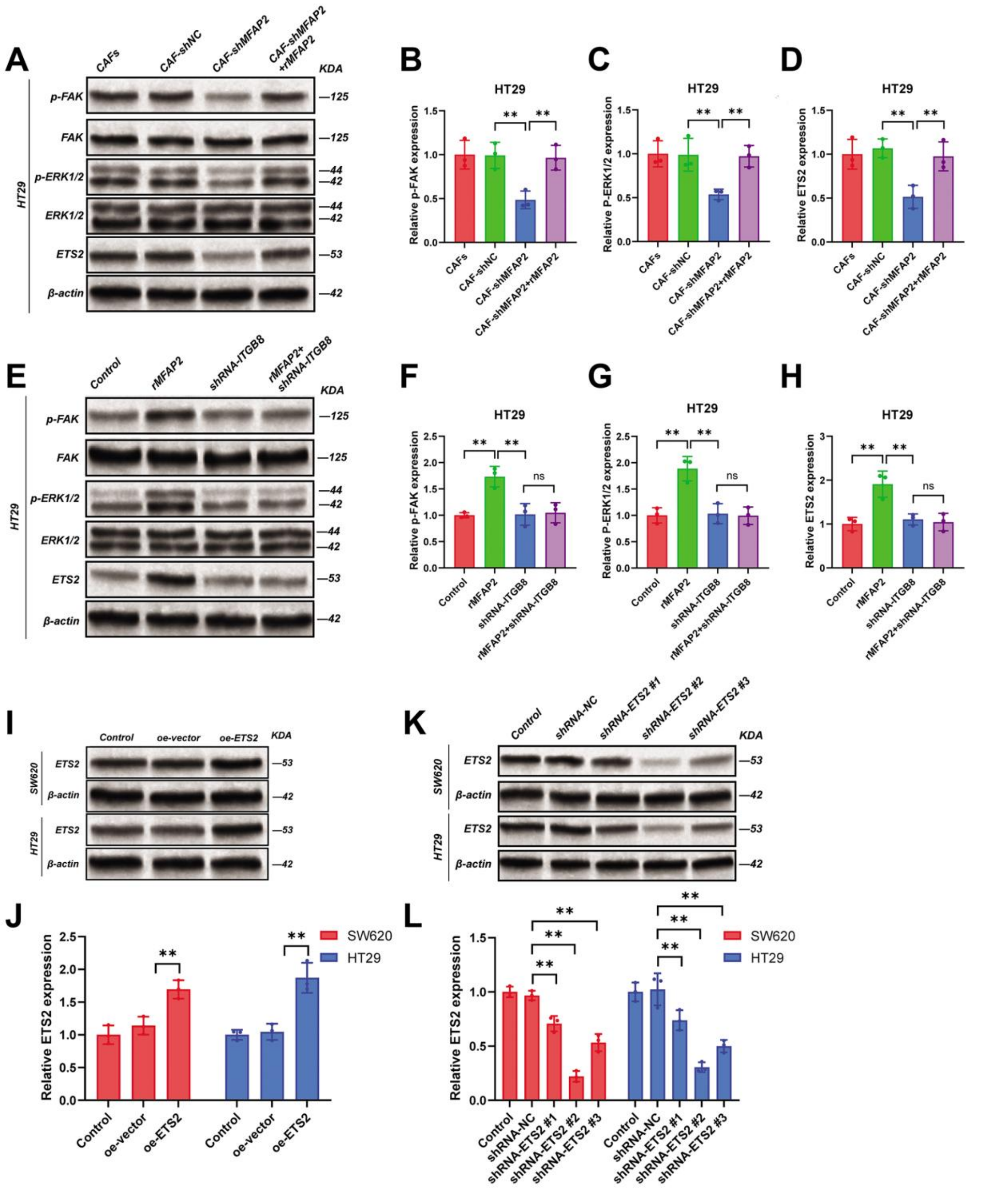

Fig.S15 MFAP2 promotes CRC progression through the ITGB8/FAK/ETS2 signaling pathway. (A) Representative Western blot images showing the expression levels of p-FAK, p-ERK1/2, and ETS2 in HT29 cells following co-culture with CAFs under conditions of MFAP2 knockdown or rMFAP2 supplementation. (B)-(D) Quantification of p-FAK, p-ERK1/2, and ETS2 protein levels shown in panel (A). (E) Western blot analysis of p-FAK, p-ERK1/2, and ETS2 in HT29 cells after ITGB8 knockdown with or without rMFAP2 treatment. (F)-(H) Quantification of p-FAK, p-ERK1/2, and ETS2 protein expression in panel (E). (I)-(J) Western blot analysis and quantification of ETS2 expression following ETS2 overexpression (OE-ETS2) in HT29 cells. (K)-(L) Western blot analysis and quantification of ETS2 expression following ETS2 knockdown using shRNA targeting ETS2 in HT29 cells. Data are presented as mean ± SD, n=3, ***P*<0.01.

16. ITGB8 silencing reduces metastasis and boosts $CD8^+$ T cell infiltration

Liver metastasis model was conducted using BALB/c mice via spleen injection to assess the impact of ITGB8 knockdown in tumor cells under different conditions. As shown in Fig.S16A-B, co-injection with CAFs or supplemented with rMFAP2 significantly increased the number of liver metastatic nodules in comparison with CT26-shNC group (P<0.01). While, compared with CT26-shNC+CAFs or CT26-shNC+rMFAP2 group, knockdown of ITGB8 in CT26 cells significantly reduced the number of gross liver metastases (P<0.01), suggesting that CAFs-derived MFAP2 might increase metastatic burden in a ITGB8 dependent manner.

To further assess the immune evasion, we analyzed the infiltration of $CD8^+$ T cells within liver metastases by flow cytometry using BALB/c. As shown in Fig.S16C-D, co-injection with CAFs or supplemented with rMFAP2 significantly reduced the infiltration of $CD8^+$ T cells in comparison with CT26-shNC group (P<0.01), which were significantly reversed by ITGB8 knockdown, indicating that CAFs-secreted MFAP2 might promote tumor progression, and that ITGB8 knockdown could restore immune infiltration, thereby inhibiting metastatic outgrowth.

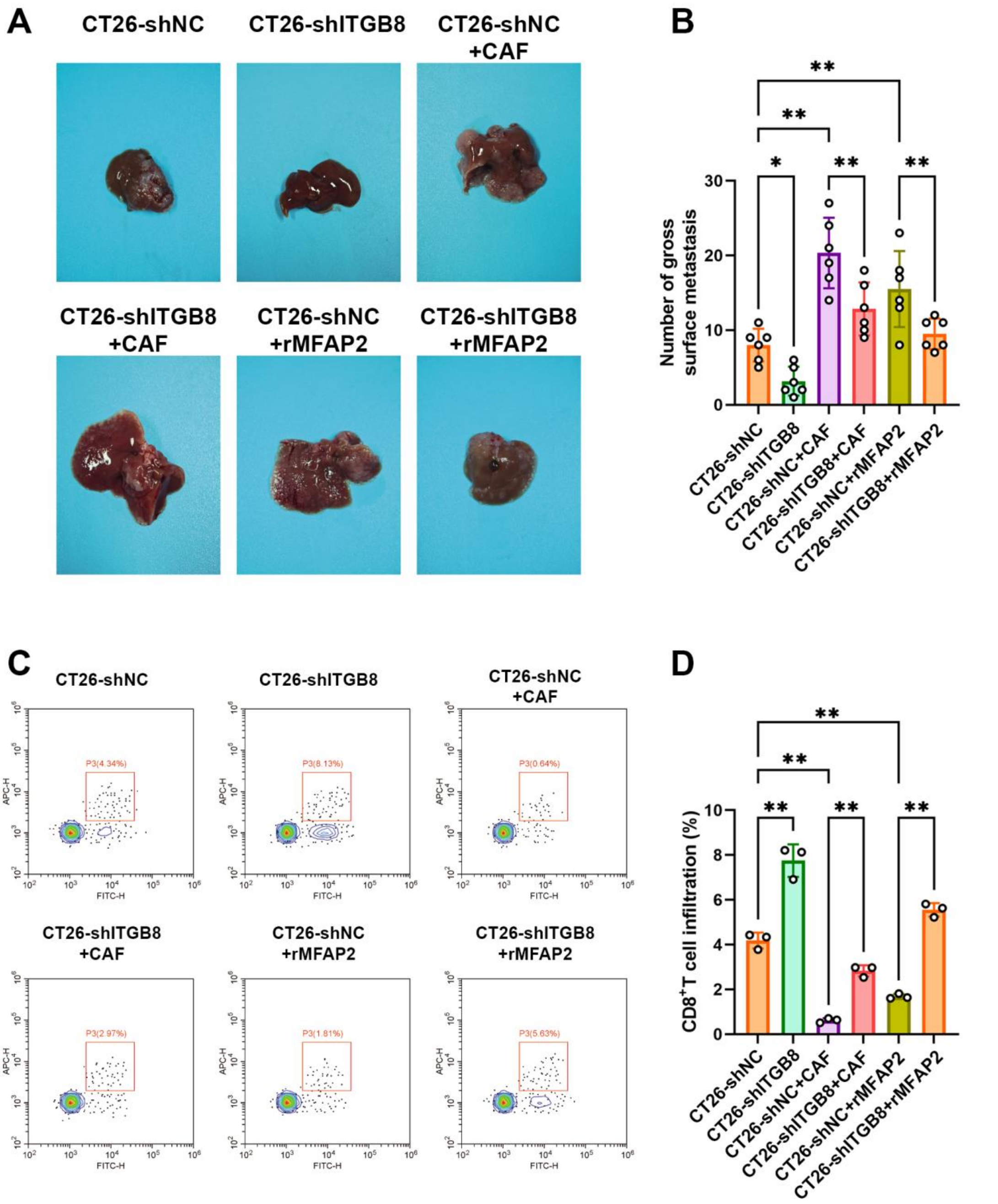

Fig.S16 ITGB8 knockdown in CT26 cells reduces liver metastasis and enhances CD8^{+} T cell infiltration. (A) Representative images of livers with metastatic nodules from mice injected with CT26 with or without CAF co-injection or rMFAP2 treatment. (B) Quantification of visible surface liver metastases in each group. (C) Representative flow cytometry plots showing the percentage of tumor-infiltrating CD3^{+}CD8^{+} T cells in liver metastases from each group. (D) Quantification of CD8^{+} T cell infiltration (%) based on flow cytometry. Data are presented as mean ± SD, n=3 or 6, **P*<0.05, ***P*<0.01.

17. Effects of ETS2 knockdown on cell viability of CRC cell lines

Given the focus of our study is emphasized on ETS2 expression in tumor cells, we conducted additional experiments to investigate whether ETS2 knockdown

directly affects tumor cell viability. As shown in Fig.S17A-B, no significant changes on cell viability were observed in the ETS2 knockdown group in comparison with shCtrl group (shNC). These results indicate that ETS2 is not essential for CRC cell viability under the tested conditions.

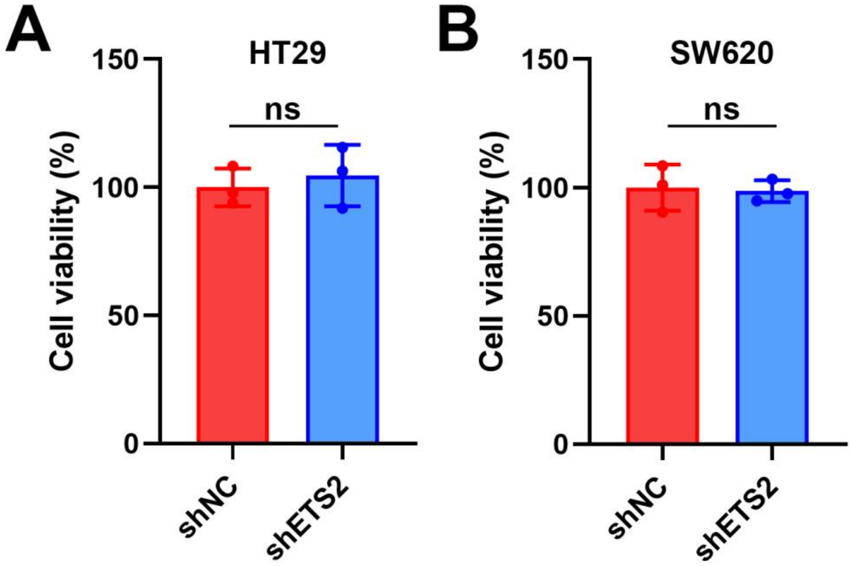

Fig.S17 Effects of ETS2 knockdown on cell viability of HT29 and SW620 Cell lines. (A) Cell viability of HT29 cells (A) and SW620 cells (B) were determined via CCK-8 following transfection with shNC or shETS2. Data are presented as mean ± SD, n=3.

18. ETS2 impairs CD8⁺ T cell-mediated anti-tumor activity by modulating cholesterol metabolism

To investigate whether ETS2 influences the cytotoxic function of $CD8^+$ T cells through cholesterol metabolic pathways, we first evaluated the migration and viability of HT29 cells co-cultured with cholesterol-pretreated $CD8^+$ T cells under different ETS2 expression conditions. As shown in Fig.S18A-B, silencing ETS2 in CRC cells significantly suppressed its invasion (P<0.01). While, ETS2 overexpression significantly increased the invasion compared with OE-vector (P<0.01). Notably, co-transfection of CYP27A1 shRNA partially reversed the invasion-promoting effect of ETS2 overexpression (P<0.01). Similarly, CCK8 assays revealed that under the pressure of activated $CD8^+$T cells, ETS2 overexpression significantly promoted the viability of HT29 cells (P<0.01), while ETS2 knockdown showed the opposite effect (P<0.01). Co-silencing of CYP27A1 abolished ETS2-overexpression induced proliferation (Fig.S18C). Given the regulatory role of cholesterol hydroxylation in immune function, we further explored the effect of 27-HC on $CD8^+$ T cell viability and apoptosis. Flow cytometry analysis revealed a dose-dependent increase in apoptosis of $CD8^+$ T cells upon exposure to increasing concentrations of 27-HC (0-3 μg/mL), with apoptosis rates rising from 4.2% to 49.87% (Fig.S18D-E). Concordantly, cell viability of CRC cell lines was significantly increased in a dose-dependent manner following 27-HC treatment (Fig.S18F), indicating that cholesterol metabolic reprogramming might directly impairs $CD8^+$ T cell survival and promote cancer cell

viability. Collectively, these findings suggest that CRC derived ETS2 might suppress CD8^{+} T cell-mediated anti-tumor immunity by promoting cholesterol metabolism and accumulation of 27-HC, leading to CD8^{+} T cell dysfunction and enhanced tumor progression.

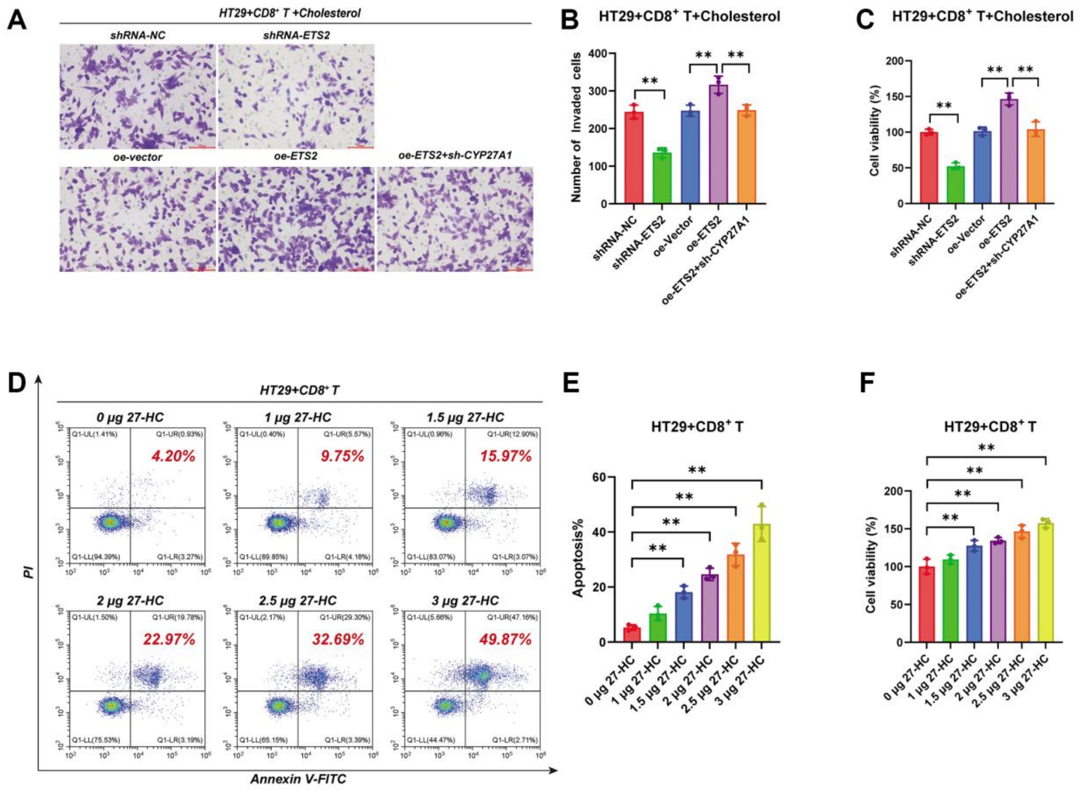

Fig.S18 ETS2 inhibits CD8^{+} T cells through the cholesterol metabolism pathway. (A) Representative images of Transwell invasion assays showing the invasion ability of HT29 cells co-cultured with cholesterol and CD8^{+} T cells under under various ETS2/CYP27A1 manipulation conditions, Scale bar=100 μm. (B) Quantification of invaded HT29 cells corresponding to (A). (C) Cell viability of HT29 cells co-cultured with activated CD8^{+} T cells under various ETS2/CYP27A1 manipulation conditions, as assessed by CCK-8 assay. (D) Flow cytometric analysis of apoptosis in CD8^{+} T cells treated with increasing concentrations of 27-HC (0-3 μg/mL). Percentages of apoptotic cells (Annexin V^{+}/PI^{-} and Annexin V^{+}/PI^{+}) are indicated in each panel. (E) Quantification of apoptosis rates of CD8^{+} T cells. (F) Viability of HT29 cells co-cultured with 27-HC and CD8^{+} T cells. Data are presented as mean ± SD, n=3, ***P*<0.01.

19. MFAP2-expressing CAFs promote 27-HC production in both in vitro and in vivo models

To elucidate the role of MFAP2 in regulating cholesterol metabolite production within the tumor microenvironment, we first measured 27-hydroxycholesterol (27-HC) levels in co-culture supernatants of CT26 colon carcinoma cells and cancer-associated fibroblasts (CAFs) with stable knockdown of MFAP2. ELISA analysis revealed that CT26 cells co-cultured with control CAFs (shNC) exhibited significantly elevated

27-HC levels compared to CT26 cells alone (Fig.S19A). In contrast, MFAP2 silencing in CAFs (shMFAP2) markedly reduced 27-HC concentrations. Importantly, reconstitution with rMFAP2 protein (shMFAP2 + rMFAP2) partially restored 27-HC production.

We next examined whether this regulatory effect persists in vivo. Orthotopic tumor models were established by co-injecting CT26 cells with corresponding CAF subtypes into syngeneic mice. Consistent with the in vitro findings, tumors formed in the presence of MFAP2-deficient CAFs exhibited significantly lower intratumoral 27-HC levels compared to those co-injected with control CAFs, while rMFAP2 supplementation restored 27-HC accumulation in tumor tissues (Fig.S19B).

These results indicate that MFAP2-expressing CAFs are critical regulator of tumor-derived 27-HC, suggesting a potential mechanism by which the stromal compartment modulates cholesterol metabolism and immunomodulatory signaling in the tumor microenvironment.

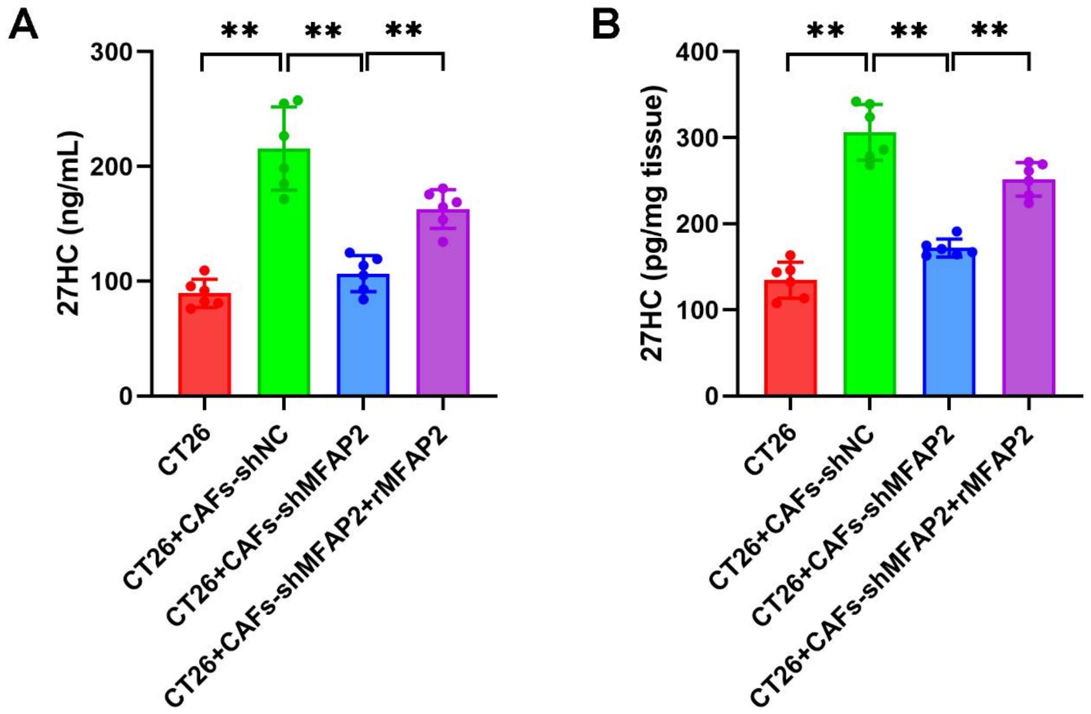

Fig.S19 MFAP2 knockdown in CAFs reduces 27-HC production in both co-culture supernatants and in vivo tumor tissues. (A) Quantification of 27-HC levels in the supernatants of CT26 cells co-cultured with CAFs under different treatment. (B) 27-HC content in tumor tissues derived from CT26 cells co-injected with corresponding CAFs in a murine orthotopic tumor model. Data are presented as mean ± SD, n=6, $^{**}P$<0.01.

20. Smad2 and Smad3 phosphorylation in response to rMFAP2 treatment

To investigate the effects of rMFAP2 on the TGF-β/Smad signaling pathway, we performed Western blot analysis on protein extracts from HT29 and SW620 cells (Albumin, ALB was introduced as control). As shown in Fig.S20, compared with HT29/SW620 alone group, addition of ALB showed no significant effect on the

phosphorylation of Smad2 and Smad3. While, addition of rMFAP2, significantly increased the level of p-Smad2 and p-Smad3 compared with HT29 alone group (P<0.01) and ALB group (P<0.01), suggesting that rMFAP2 might increased the activation of TGF-β/Smad signaling pathway in CRC cells.

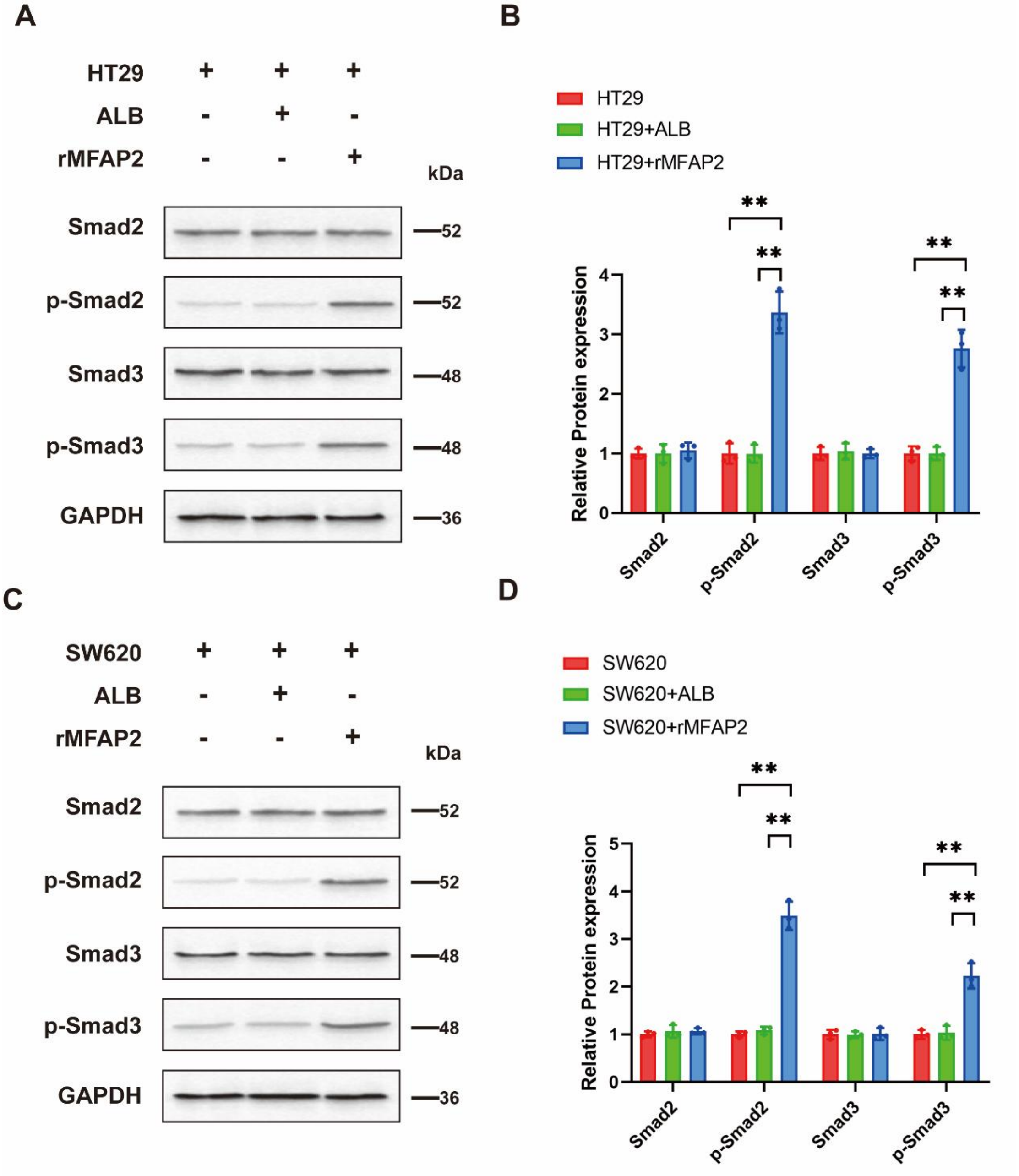

Fig.S20 Effect of rMFAP2 on the activation of Smad2/3 pathway in CRC cell lines. The representative Western blot bands of p-Smad2 and p-Smad3 in HT29 (A) and SW620 (C). The quantitative analysis of p-Smad2 and p-Smad3 in HT29 (B) and SW620 (D). Data are presented as mean ± SD, n=3, **P<0.01.

21. CAFs-derived MFAP2 could directly reduce CD8$^+$ T cell cytotoxicity and induce apoptosis of CD8$^+$ T cells

As shown in Fig.S21, to further investigate the influence of CAFs-derived MFAP2 on CD8$^+$ T cell functionality, we co-cultured CD8$^+$ T cells with CAFs

transfected with control shRNA, MFAP2-targeting shRNA, or CAFs-shMFAP2 supplemented with rMFAP2. Flow cytometry analysis revealed that MFAP2 knockdown in CAFs significantly increased the proportion of Granzyme B^{+}IFN-γ^{+} CD8^{+} T cells (active CD8^{+} T cell), indicating enhanced cytotoxic function (P<0.01). In contrast, supplementation with rMFAP2 partially reversed this activation phenotype (P<0.01).

Additionally, Annexin V/PI staining demonstrated that CD8^{+} T cells co-cultured with MFAP2-deficient CAFs exhibited a marked reduction in apoptosis compared to those with CAFs-shCtrl (P<0.01), while exogenous MFAP2 restored apoptotic levels (P<0.01). These findings suggest that CAFs-derived MFAP2 suppresses the cytotoxic activity and promotes the apoptosis of CD8^{+} T cells, thereby contributing to an immunosuppressive tumor microenvironment.

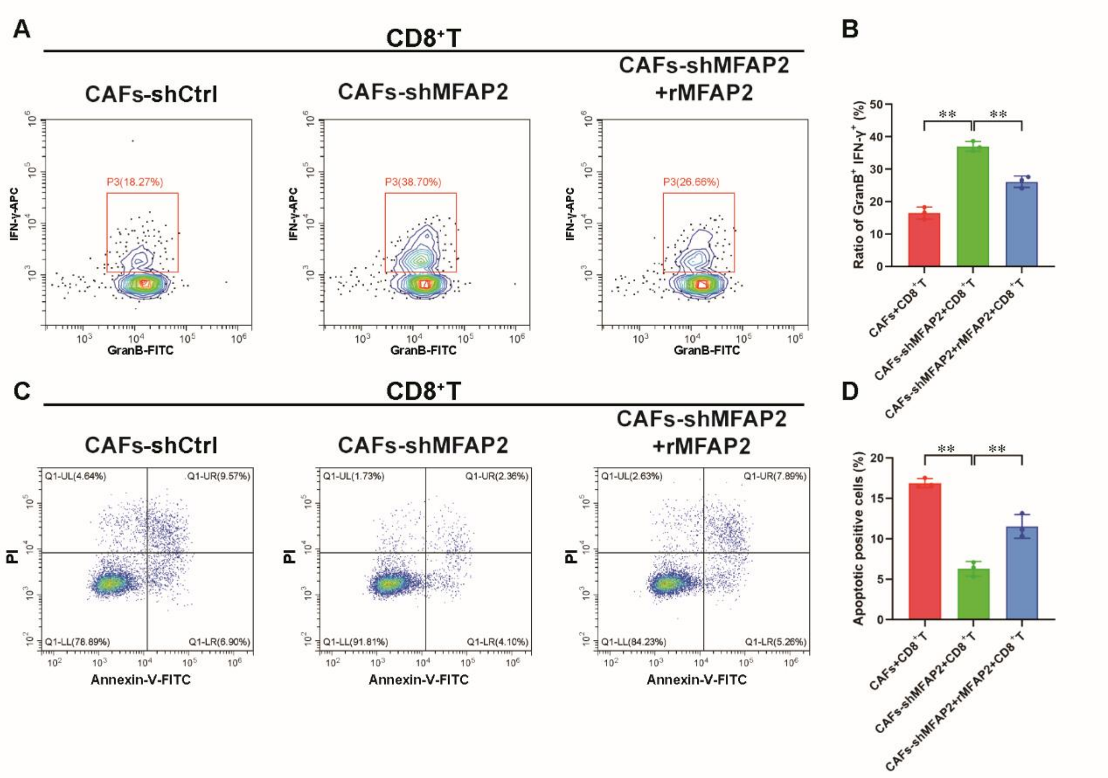

Fig.S21 CAFs-derived MFAP2 reduced CD8^{+} T cell cytotoxicity and induced apoptosis of CD8^{+} T cells. (A) Representative flow cytometry plots showing the expression of Granzyme B and IFN-γ in CD8^{+} T cells co-cultured with CAFs. (B) Quantification of Granzyme B^{+}IFN-γ^{+} CD8^{+} T cells. (C) Representative flow cytometry plots showing Annexin V and PI staining in CD8^{+} T cells under the same co-culture conditions. (D) Quantification of apoptotic CD8^{+} T cells from (C). Data are presented as mean ± SD, n=3, **P<0.01.

Supplementary tables

Table.S1 The Target Sequences of shRNA

Gene	Sequence (5'-3')
Human MFAP2#1	CCGG-GTACGTCATTAACAAGGAGAT-CTCGAG-ATCTCCTTGTTAATGACGTAC-TTTTTG
Human MFAP2#2	CCGG-CCGTGTGTACGTCATTAACAA-CTCGAG-TTGTTAATGACGTACACACGG-TTTTTG
Human MFAP2#3	CCGG-GACAACCCAGACTACTATGAT-CTCGAG-ATCATAGTAGTCTGGGTTGTC-TTTTTG
Mouse MFAP2#1	CCGG-CAGCAGCAAGTTCAACAGGAA-CTCGAG-TTCCTGTTGAACTTGCTGCTG-TTTTTG
Mouse MFAP2#2	CCGG-GCCTTGCAAACAGTGTCTCAA-CTCGAG-TTGAGACACTGTTTGCAAGGC-TTTTTG
Mouse MFAP2#3	CCGG-GACTACCAAGAAGTGAGTCCT-CTCGAG-AGGACTCACTTCTTGGTAGTC-TTTTTG
Human ETS2#1	CCGG-CCTGACTTTGTGGGTGACATT-CTCGAG-AATGTCACCCACAAAGTCAGG-TTTTTG
Human ETS2#2	CCGG-GCTGTGATGAGTCAAGCCTTA-CTCGAG-TAAGGCTTGACTCATCACAGC-TTTTTG
Human ETS2#3	CCGG-CCAACCATGTCTTTCAAGGAT-CTCGAG-ATCCTTGAAAGACATGGTTGG-TTTTTG
Mouse ETS2#1	CCGG-CCTCAGTGGATCAACAGCAAT-CTCGAG-ATTGCTGTTGATCCACTGAGG-TTTTTG
Mouse ETS2#2	CCGG-CCGTCAATGTCAATTACTGTT-CTCGAG-AACAGTAATTGACATTGACGG-TTTTTG
Mouse ETS2#3	CCGG-GCTACGTGTACCGTTTCGTAT-CTCGAG-ATACGAAACGGTACACGTAGC-TTTTTG
Human ITGB1#1	CCGG-GCCTTGCATTACTGCTGATAT-CTCGAG-ATATCAGCAGTAATGCAAGGC-TTTTTG
Human ITGB1#2	CCGG-CCAAATCATGTGGAGAATGTA-CTCGAG-TACATTCTCCACATGATTTGG-TTTTTG
Human ITGB1#3	CCGG-GCCCTCCAGATGACATAGAAA-CTCGAG-TTTCTATGTCATCTGGAGGGC-TTTTTG
Human ITGB2#1	CCGG-GAAACCCAGGAAGACCACAAT-CTCGAG-ATTGTGGTCTTCCTGGGTTTC-TTTTTG
Human ITGB2#2	CCGG-CCATCTCATTAAGAATGCTTA-CTCGAG-TAAGCATTCTTAATGAGATGG-TTTTTG
Human ITGB2#3	CCGG-GCACCCTGATAAGCTGCGAAA-CTCGAG-TTTCGCAGCTTATCAGGGTGC-TTTTTG
Human ITGB3#1	CCGG-CCACGTCTACCTTCACCAATA-CTCGAG-TATTGGTGAAGGTAGACGTGG-TTTTTG
Human ITGB3#2	CCGG-CCTTAGCCTTTGTCCCAGAAT-CTCGAG-ATTCTGGGACAAAGGCTAAGG-TTTTTG

Human ITGB3#3	CCGG-GATGCAGTGAATTGTACCTAT-CTCGAG-ATAGGTACAATTCACTGCATC-TTTTTG
Human ITGB4#1	CCGG-GAGGGTGTCATCACCATTGAA-CTCGAG-TTCAATGGTGATGACACCCTC-TTTTTG
Human ITGB4#2	CCGG-GAGAAGCTTCACACCTATTTC-CTCGAG-GAAATAGGTGTGAAGCTTCTC-TTTTTG
Human ITGB4#3	CCGG-GTGGATGAGTTCCGGAATAAA-CTCGAG-TTTATTCCGGAACTCATCCAC-TTTTTG
Human ITGB5#1	CCGG-CCCGCTATGAAATGGCTTCAA-CTCGAG-TTGAAGCCATTTCATAGCGGG-TTTTTG
Human ITGB5#2	CCGG-GCATCCAACCAGATGGACTAT-CTCGAG-ATAGTCCATCTGGTTGGATGC-TTTTTG
Human ITGB5#3	CCGG-CTGAGGGCAAACCTTGTCAAA-CTCGAG-TTTGACAAGGTTTGCCCTCAG-TTTTTG
Human ITGB6#1	CCGG-CCATTGACAAATGATGCTGAA-CTCGAG-TTCAGCATCATTTGTCAATGG-TTTTTG
Human ITGB6#2	CCGG-CCGAGAAGAATGTGTGGACAA-CTCGAG-TTGTCCACACATTCTTCTCGG-TTTTTG
Human ITGB6#3	CCGG-GAAACATTTATGGGCCTTATT-CTCGAG-AATAAGGCCCATAAATGTTTC-TTTTTG
Human ITGB7#1	CCGG-GCACAGAGTTTGACTACCCTT-CTCGAG-AAGGGTAGTCAAACTCTGTGC-TTTTTG
Human ITGB7#2	CCGG-ACCACCATCAATCCTCGCTTT-CTCGAG-AAAGCGAGGATTGATGGTGGT-TTTTTG
Human ITGB7#3	CCGG-GCTGAGTAAACTGATTCCTAA-CTCGAG-TTAGGAATCAGTTTACTCAGC-TTTTTG
Human ITGB8#1	CCGG-GCTCAGTTGATTCAATAGAAT-CTCGAG-ATTCTATTGAATCAACTGAGC-TTTTTG
Human ITGB8#2	CCGG-CGAGCAATGATGAAGTTCTTT-CTCGAG-AAAGAACTTCATCATTGCTCG-TTTTTG
Human ITGB8#3	CCGG-CCCAGCACTGTGTCAATTCAA-CTCGAG-TTGAATTGACACAGTGCTGGG-TTTTTG
Mouse ITGB8#1	CCGG-GCTGCAAATCTCAACAATTTA-CTCGAG-TAAATTGTTGAGATTTGCAGC-TTTTTG
Mouse ITGB8#2	CCGG-GCCCAAGCTATCTGCGAATAT-CTCGAG-ATATTCGCAGATAGCTTGGGC-TTTTTG
Mouse ITGB8#3	CCGG-GCCAAAGTGAACACAATAGAT-CTCGAG-ATCTATTGTGTTCACTTTGGC-TTTTTG
Human CYP27A1#1	CCGG-GCTTTCAATGAGGTGATTGAT-CTCGAG-ATCAATCACCTCATTGAAAGC-TTTTTG
Human CYP27A1#2	CCGG-GCACCAGTTACAGGTGCTTTA-CTCGAG-TAAAGCACCTGTAACTGGTGC-TTTTTG
Human CYP27A1#3	CCGG-CGATACCTGGATGGTTGGAAT-CTCGAG-ATTCCAACCATCCAGGTATCG-TTTTTG

Table.S2 Antibody Information

Antibodies	Cat NO.	Company
MFAP2	sc-166077	Santa Cruz
N-cadherin	14215/13316	Cell Signaling Technology
E-cadherin	Cat#14-3249-82	Invitrogen
Smad2	12570-1-AP	Proteintech
p-Smad2	AP1342	ABclonal
Smad3	ab208182	Abcam
p-Smad3	AP0727	ABclonal
EPCAM	A25344	ABclonal
PAN-CK	26411-1-AP	Proteintech
α-SMA	A2235	ABclonal
CD3	17617-1-AP	Proteintech
CD28	65099-1-Ig/98195-1-RR	Proteintech
CD3	Cat # 11-0032-82	Invitrogen
CD8a	Cat # 14-0808-82	Invitrogen
CD8a	Cat # 17-0081-82	Invitrogen
Granzyme B	515403	BioLegend
IFN-γ	554413	BD Biosciences
GAPDH	10494-1-AP	Proteintech
Vimentin	5741	Cell Signaling Technology
p-FAK	3283	Cell Signaling Technology
FAK	71433	Cell Signaling Technology
p-ERK1/2	4370	Cell Signaling Technology
ERK1/2	9102	Cell Signaling Technology
ITGB1	12594-1-AP	Proteintech
ITGB2	A25810	ABclonal

ITGB3	18309-1-AP	Proteintech
ITGB4	21738-1-AP	Proteintech
ITGB5	28543-1-AP	Proteintech
ITGB6	28378-1-AP	Proteintech
ITGB7	11328-1-AP	Proteintech
ITGB8	PA5-100843	Thermo Fisher Scientific
Snail	AF66032	Affinity
ETS2	12280-1-AP	Proteintech
CYP27A1	ab126785	Abcam
LXRβ	ab28479	Abcam
β-actin	ab8226	Abcam

Table.S3 Antibodies and Dilution

Antibodies	Dilution (application)
CD3	1 μg/mL (Cell treatment), 1:500 (Flow)
CD28	2 μg/mL (Cell treatment)
Gran B	1:500 (Flow)
IFN-γ	1:300 (Flow)
MFAP2	1:500 (WB), 1:200 (IF)
E-cadherin	1:1000 (WB), 1:100 (IF)
N-cadherin	1:1000 (WB), 1:100 (IF)
Vimentin	1:1000 (WB)
Snail	1:500 (WB)
p-FAK	1:1000 (WB)
FAK	1:1000 (WB)
p-ERK1/2	1:2000 (WB)
ERK1/2	1:1000 (WB)
ETS2	1:500 (WB)
CYP27A1	1:1000 (WB)
LXRβ	2 μg/mL (WB)
β-actin	1:10000 (WB)
Smad2	1:1000 (WB)
p-Smad2	1:1000 (WB)
Smad3	1:3000 (WB)

Antibodies	Dilution (application)
p-Smad3	1:3000 (WB)
GAPDH	1:5000 (WB)
EPCAM	1:200 (IF)
PAN-CK	1:200 (IF)
α-SMA	1:300 (IHC), 1:400 (Flow)
CD8	1:300 (IHC), 1:200 (Flow)
ITGB1	1:4000 (WB)
ITGB2	1:1000 (WB)
ITGB3	1:2000 (WB)
ITGB4	1:500 (WB)
ITGB5	1:500 (WB)
ITGB6	1:1000 (WB)
ITGB7	1:1000 (WB)
ITGB8	1:1000 (WB), 1:200 (IF)
