# Supplementary material for: Cancer-associated fibroblasts (CAFs) derived from MFAP2 promote CRC proliferation and metastasis while suppressing CD8+ T cell-mediated antitumor immunity
Source: Cell Death Dis. 2026 Jan 30;17(1):159. doi: 10.1038/s41419-026-08413-w (PMC12877200; doi:10.1038/s41419-026-08413-w)
Supplement: Supplementary file 2 — Original Raw Data Western blots [file 41419_2026_8413_MOESM2_ESM.pptx]

## Slide 1
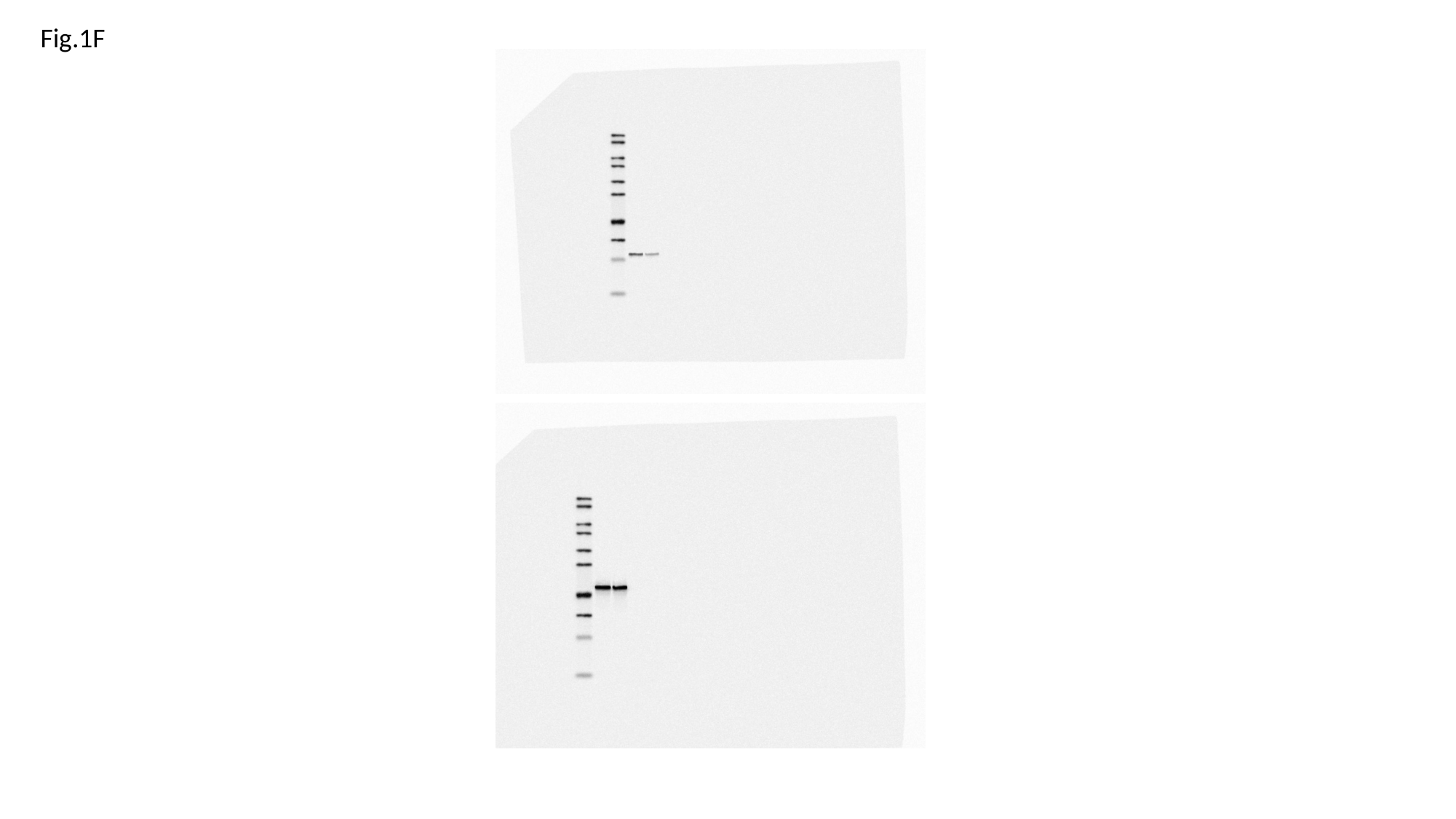

Fig.1F

## Slide 2
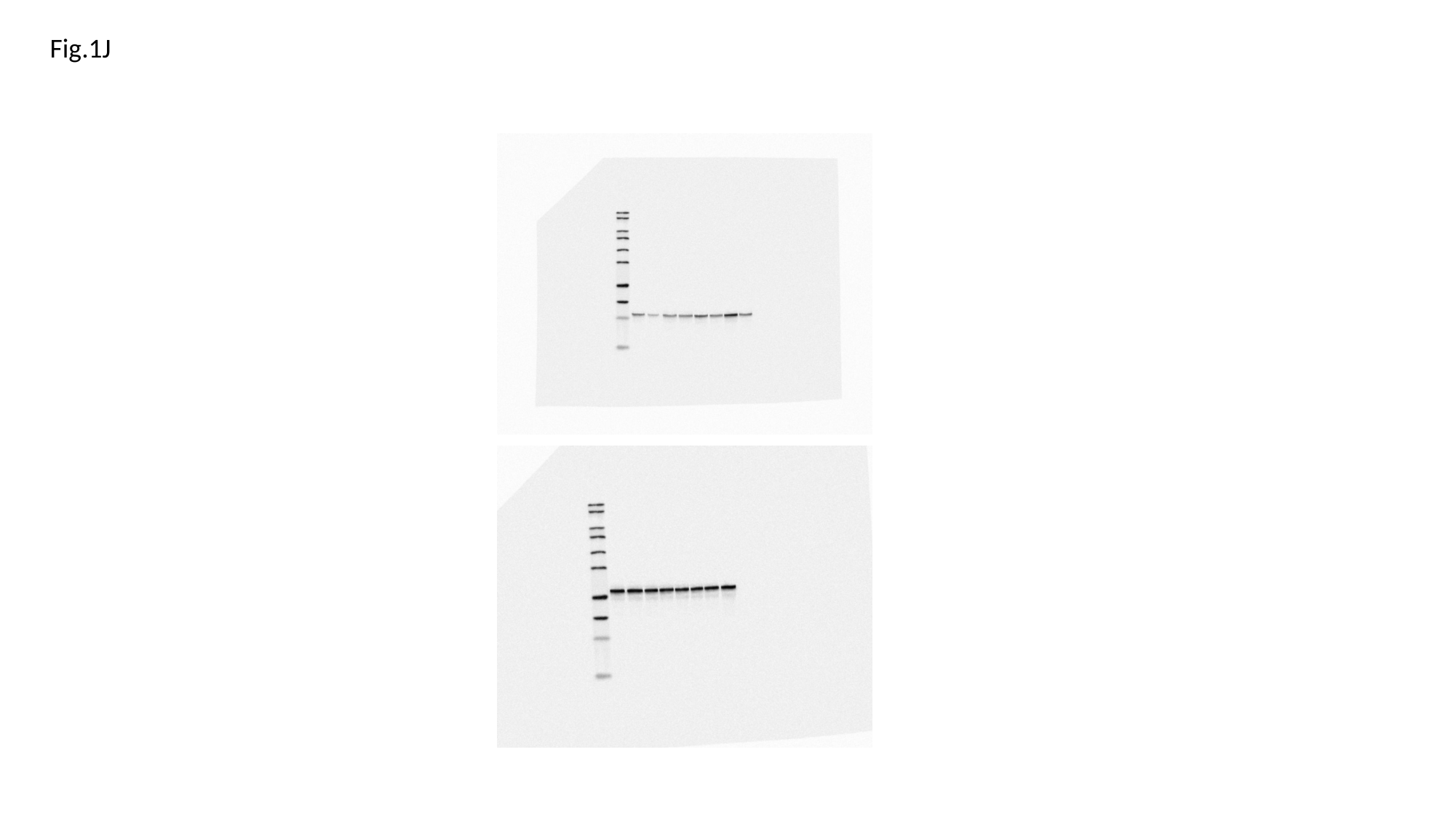

Fig.1J

## Slide 3
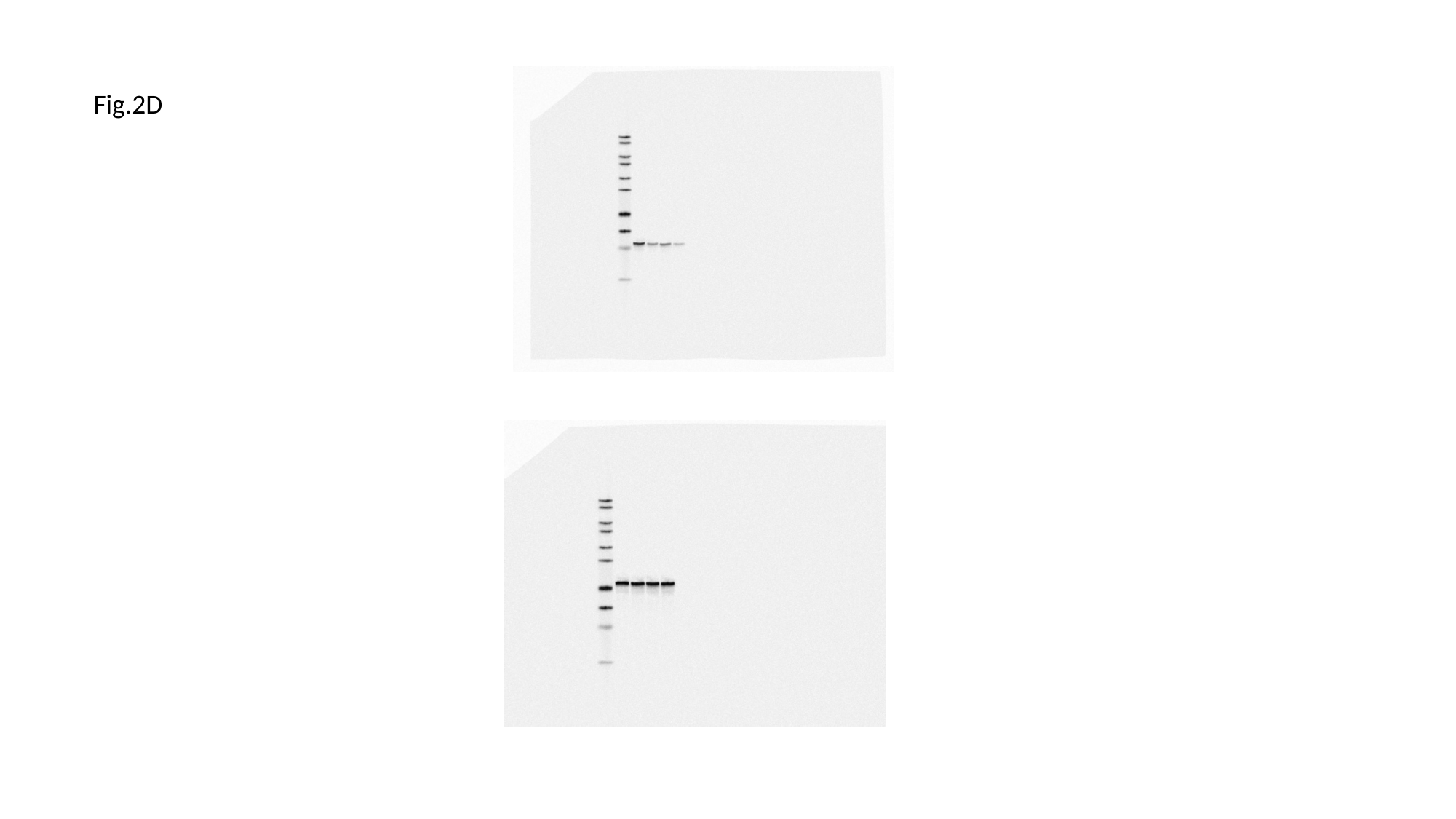

Fig.2D

## Slide 4
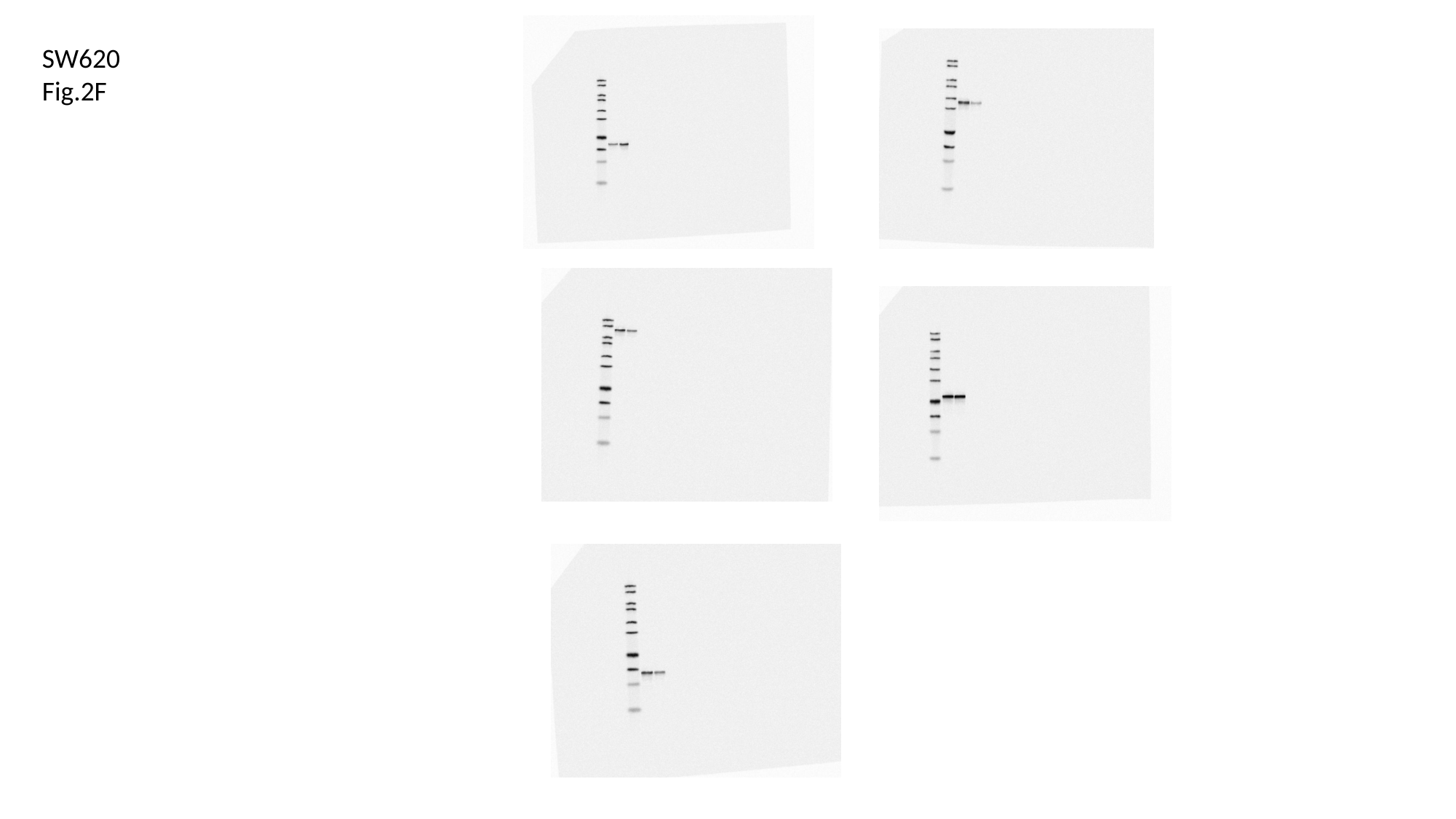

SW620
Fig.2F

## Slide 5
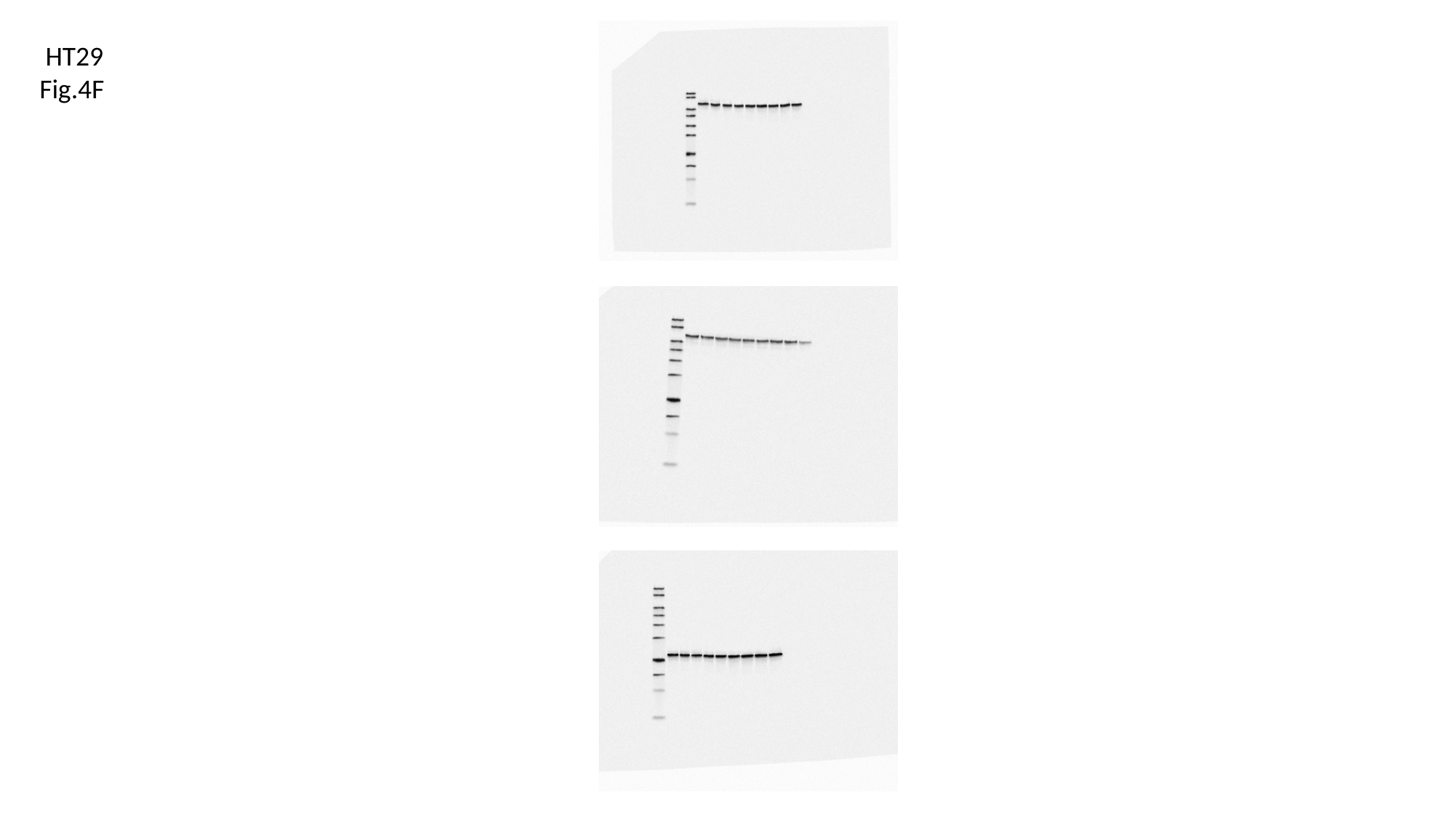

HT29
Fig.4F

## Slide 6
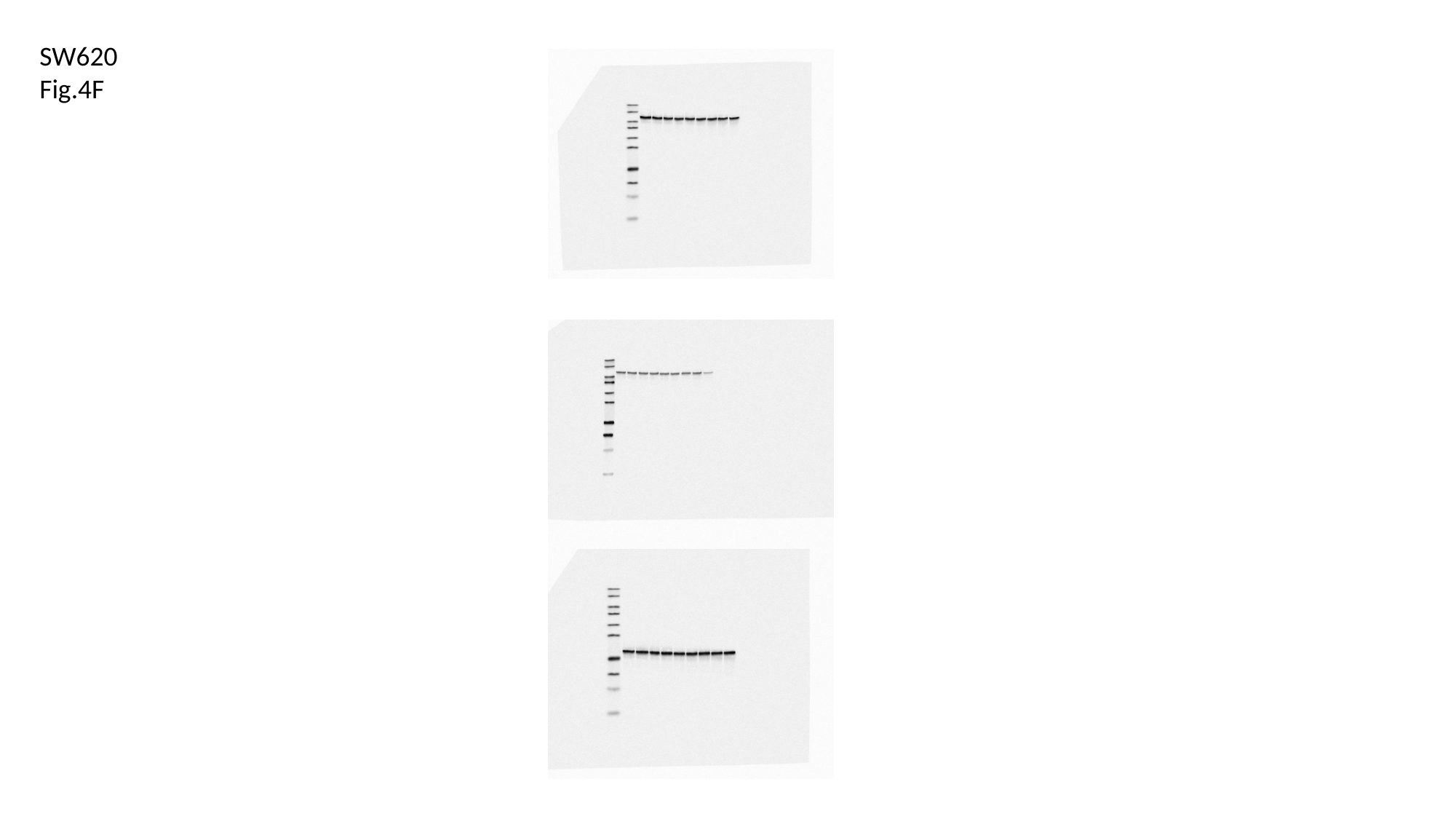

SW620
Fig.4F

## Slide 7
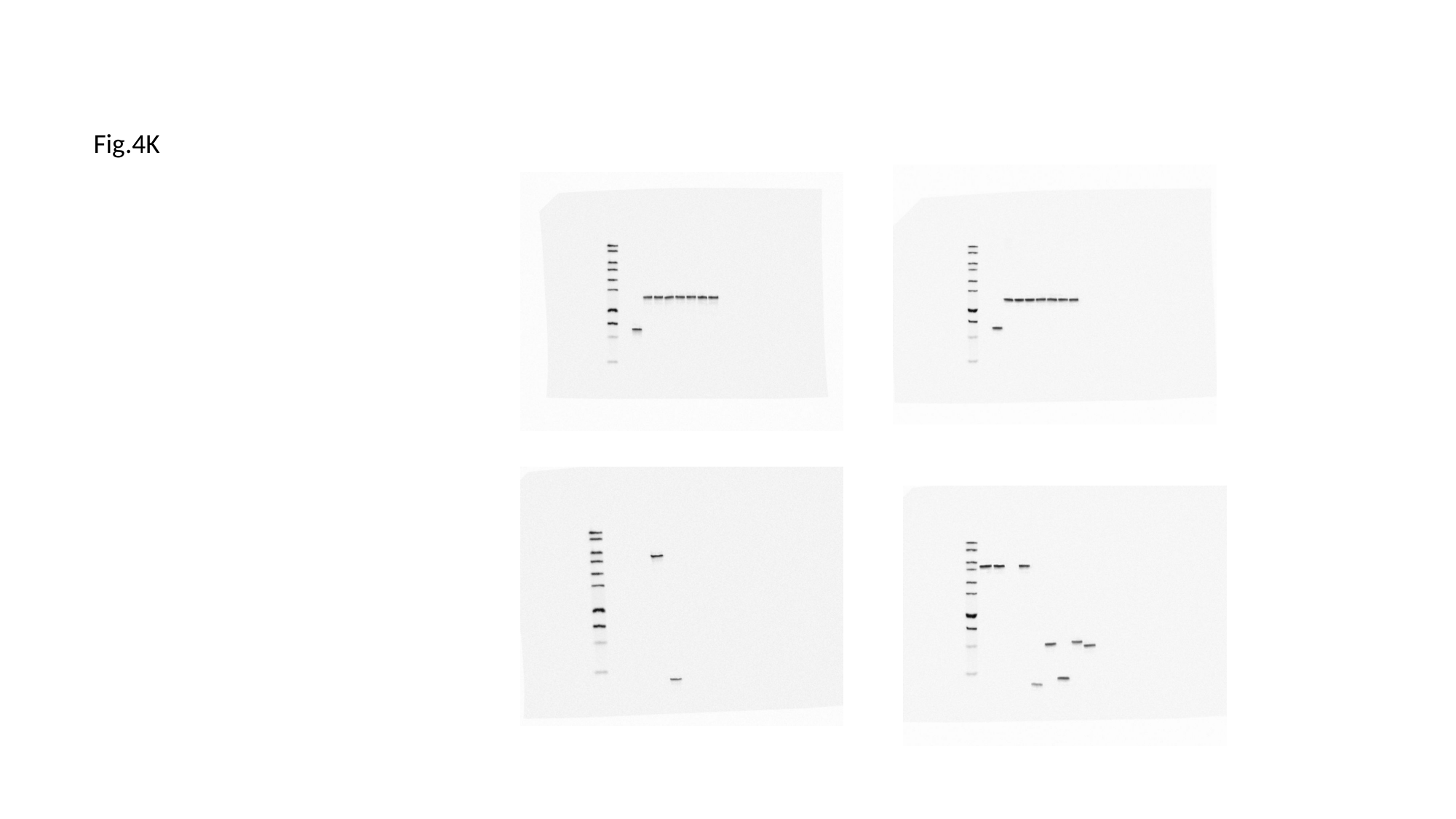

Fig.4K

## Slide 8
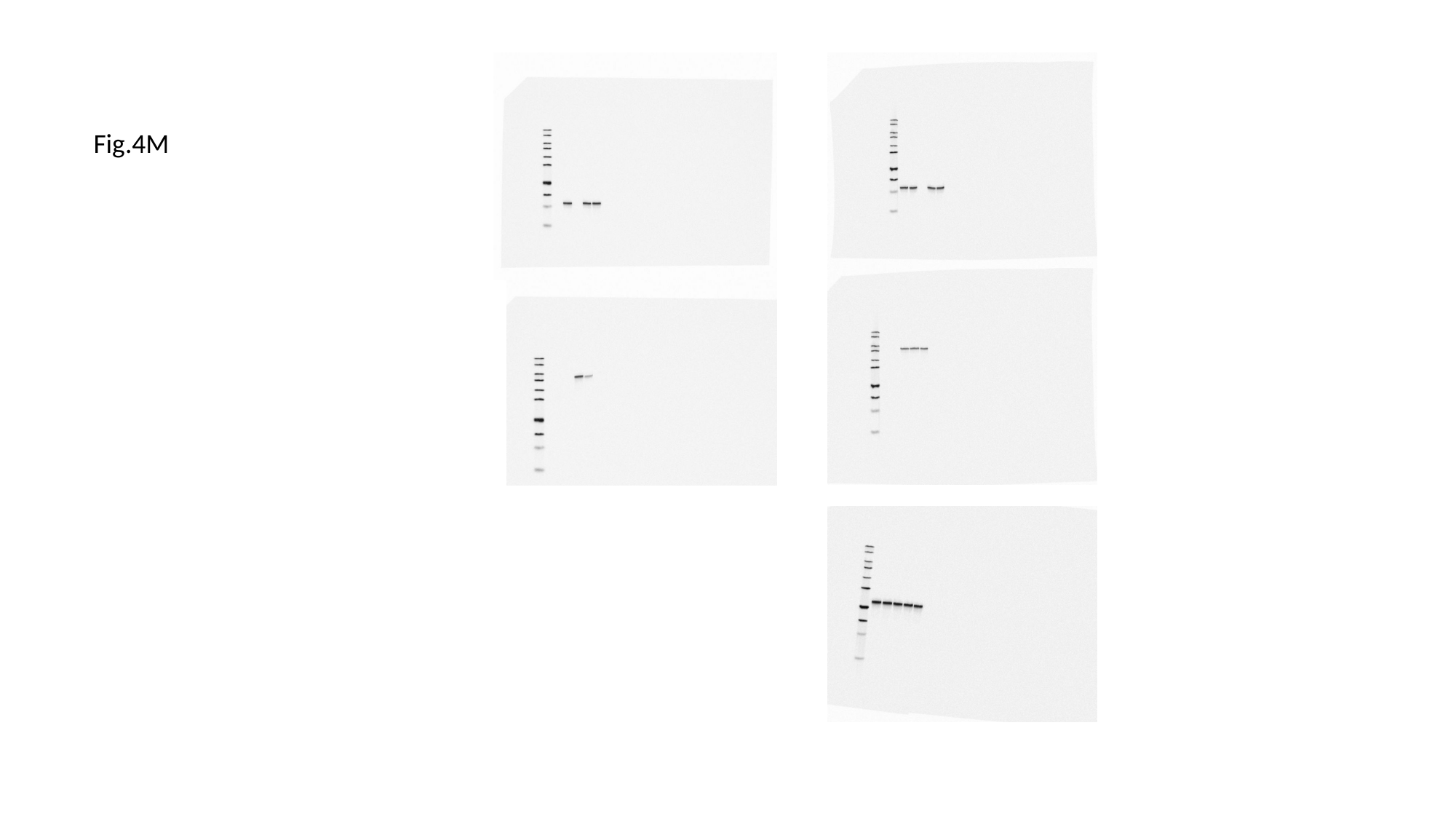

Fig.4M

## Slide 9
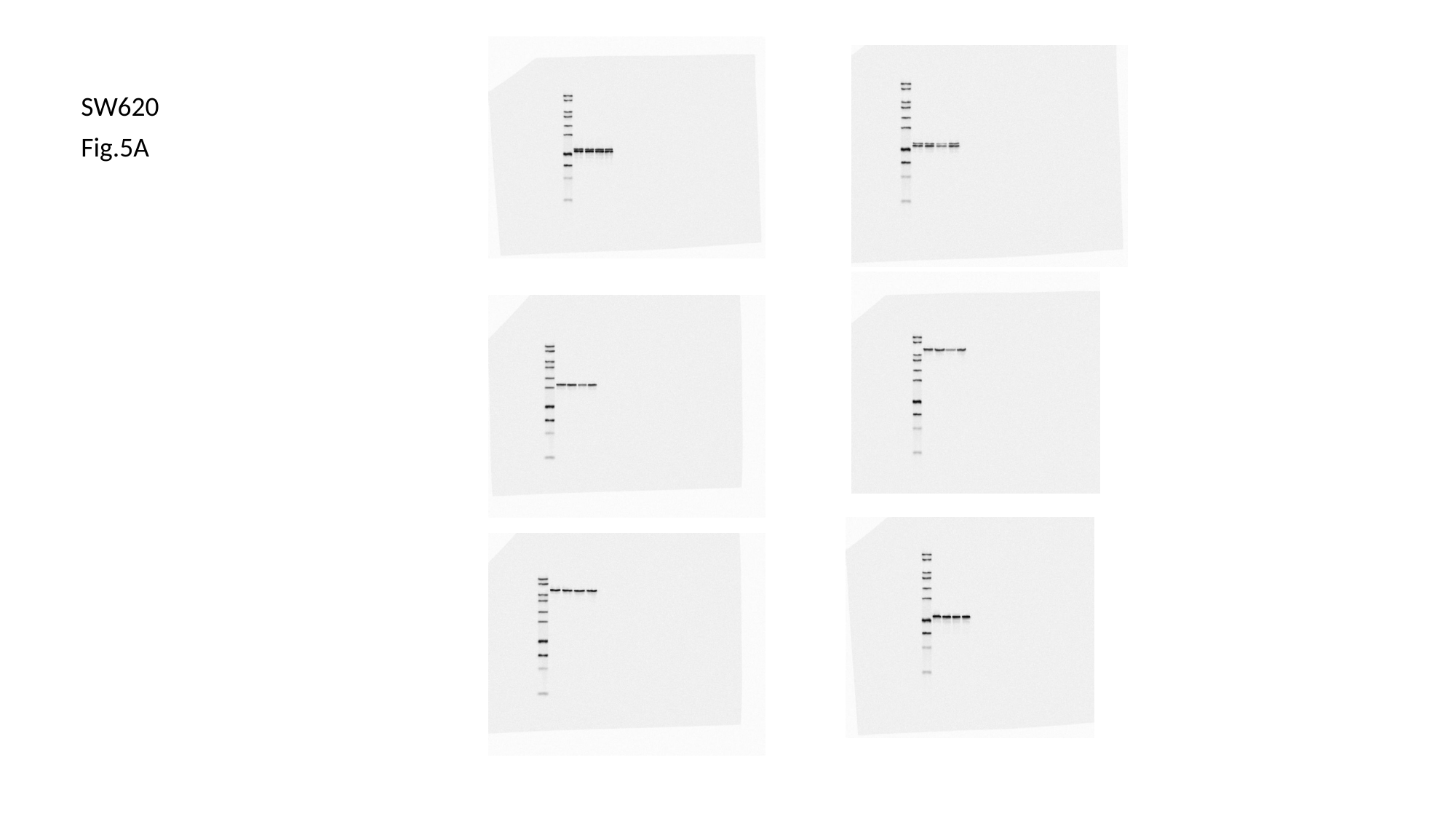

SW620
Fig.5A

## Slide 10
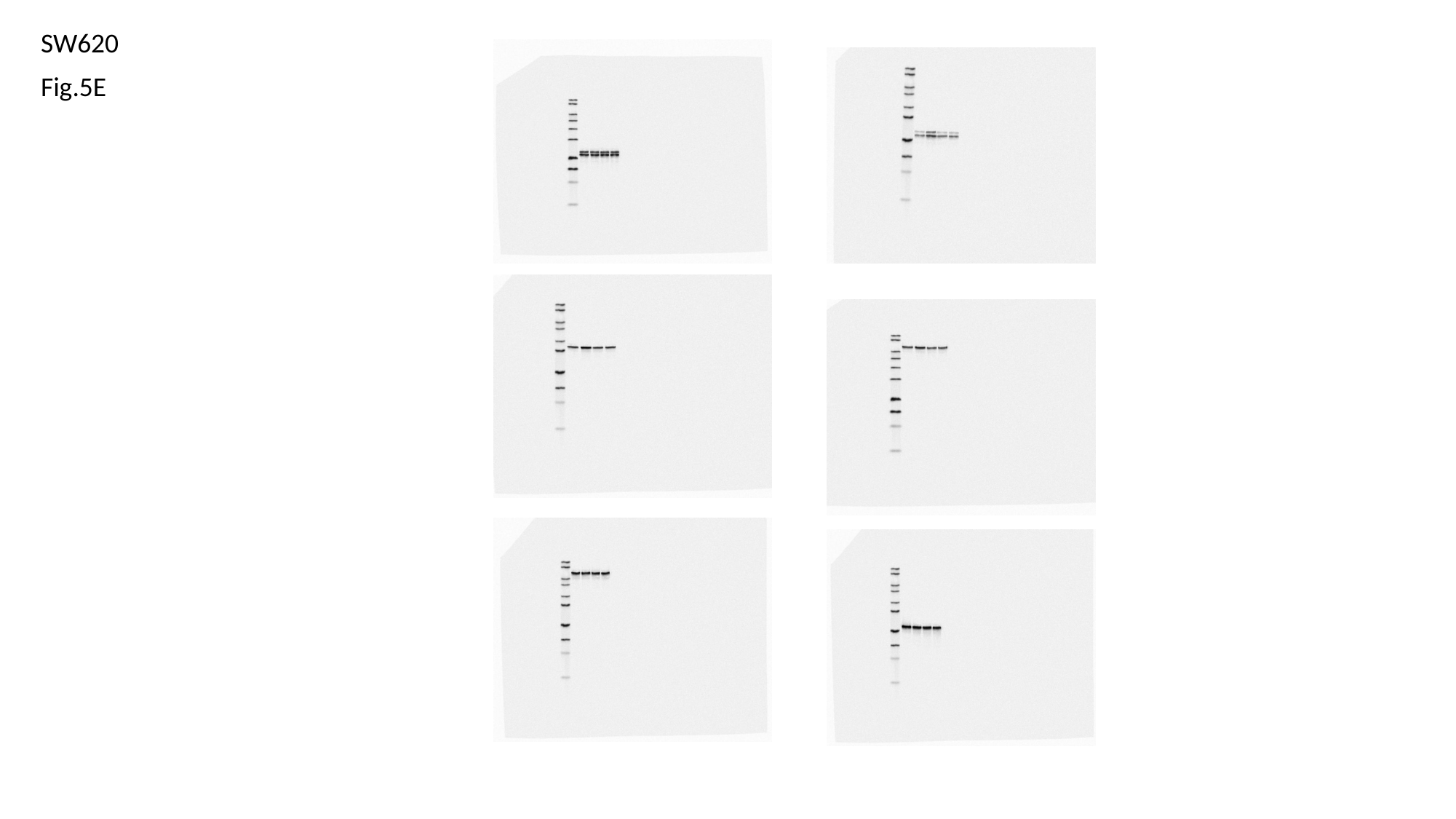

SW620
Fig.5E

## Slide 11
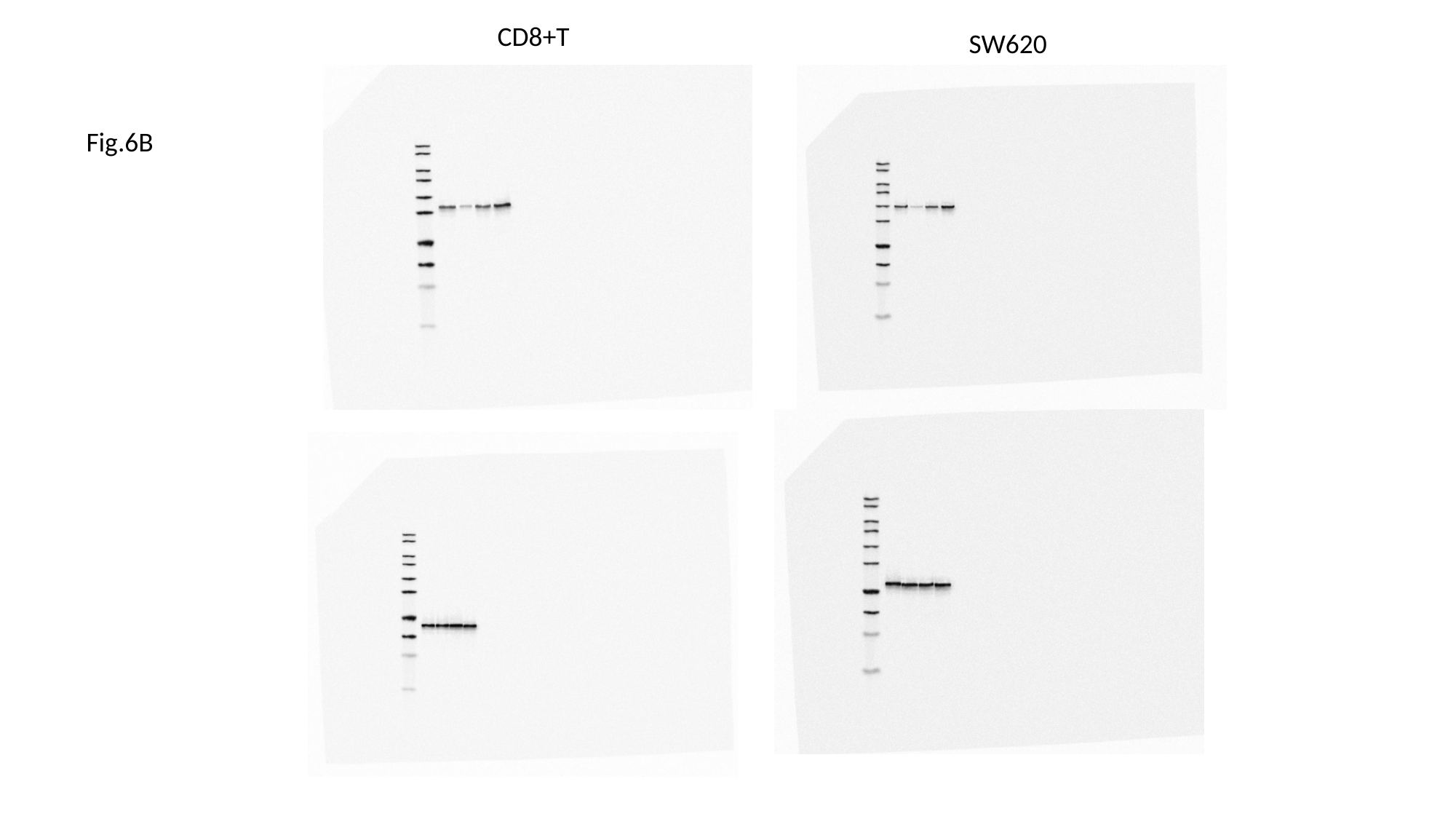

CD8+T
SW620
Fig.6B

## Slide 12
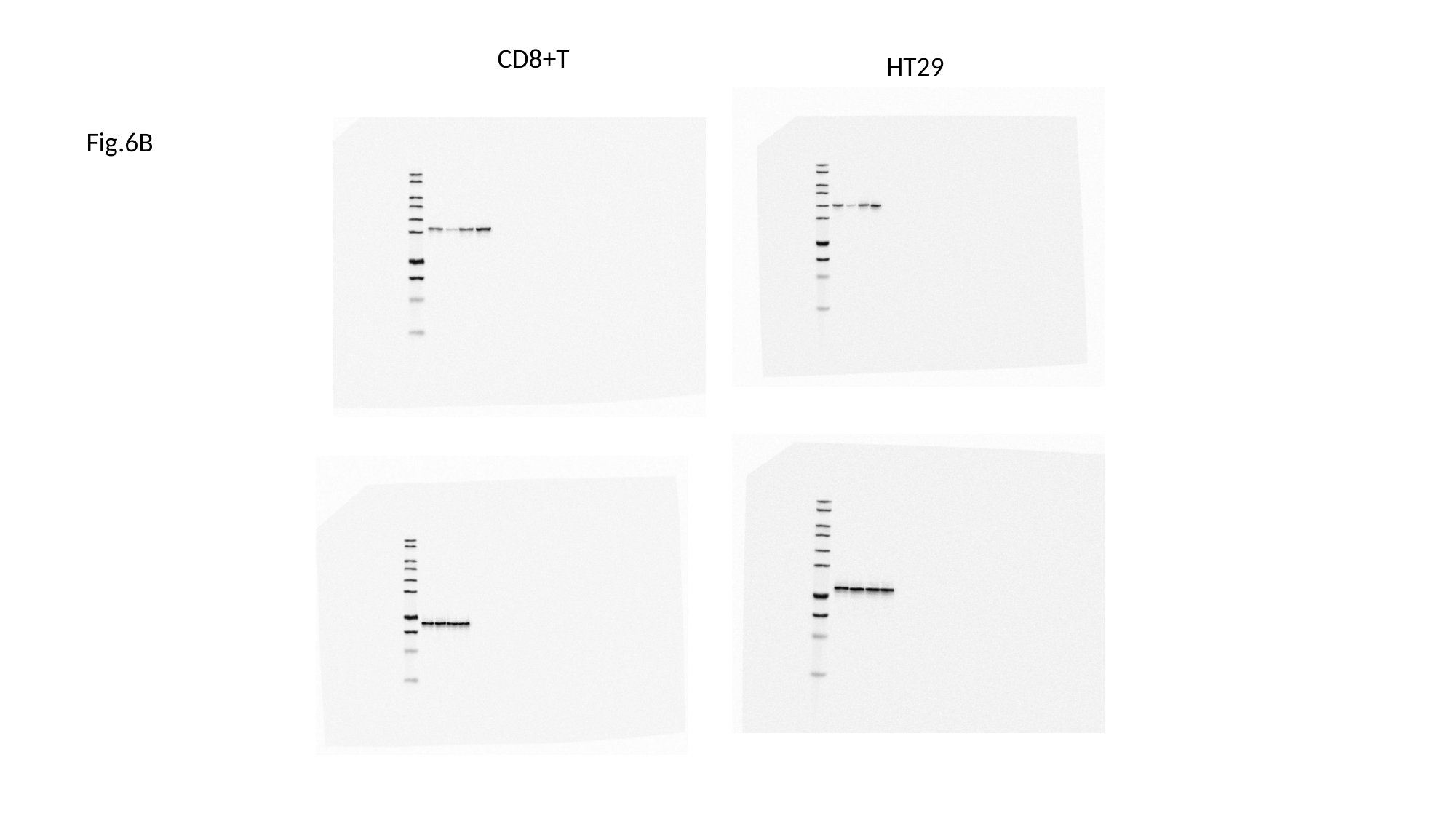

CD8+T
HT29
Fig.6B

## Slide 13
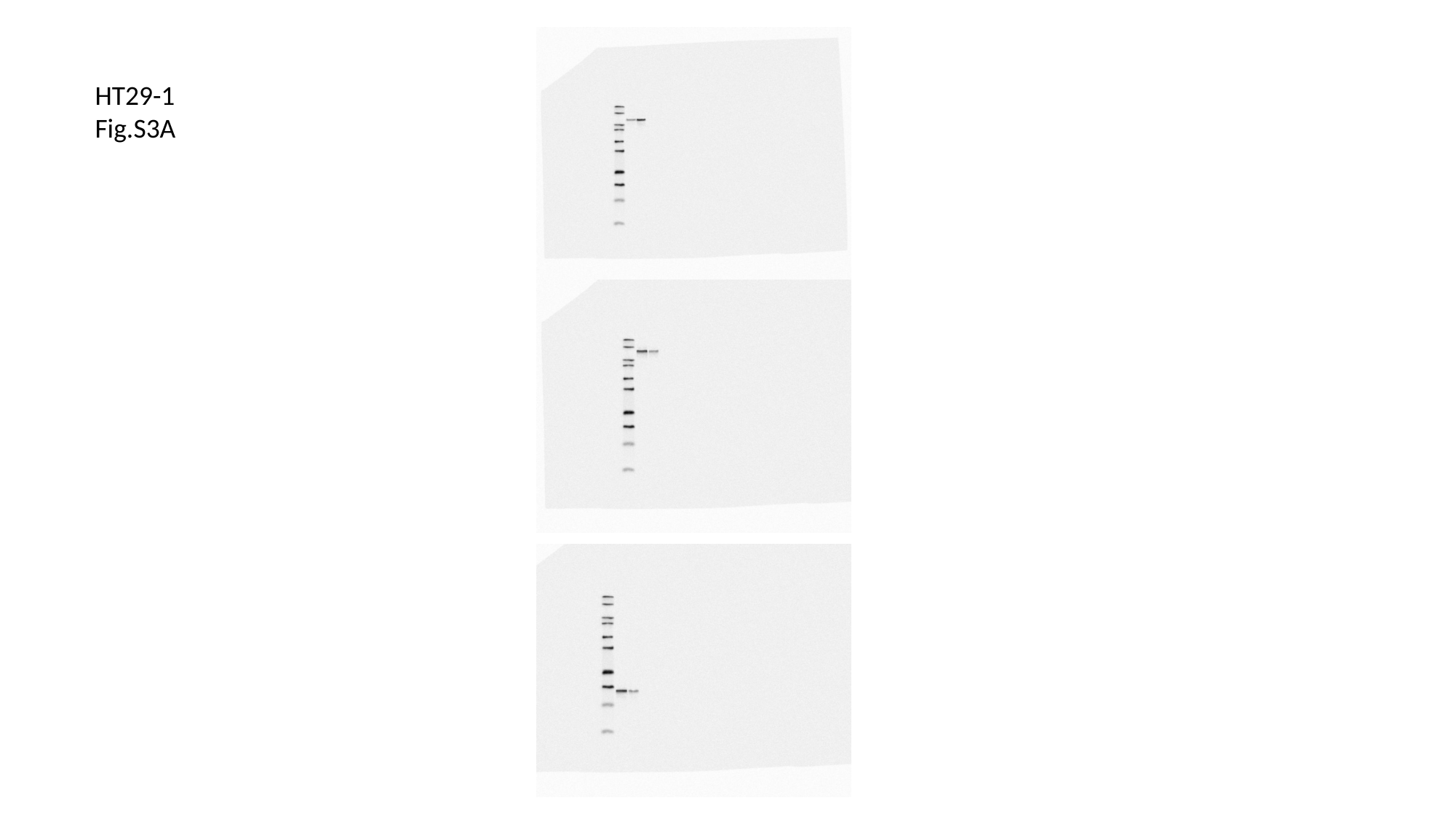

HT29-1
Fig.S3A

## Slide 14
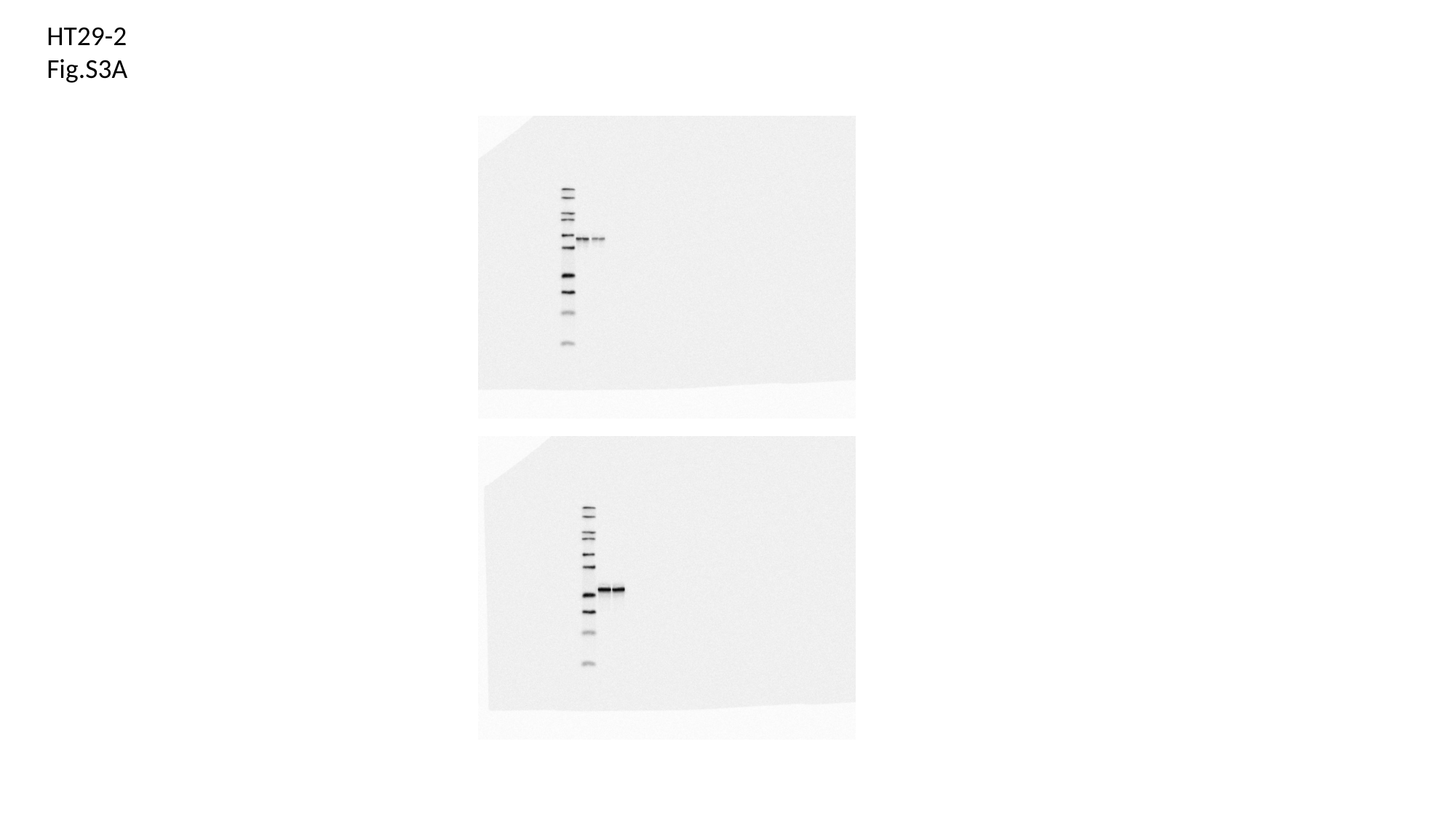

HT29-2
Fig.S3A

## Slide 15
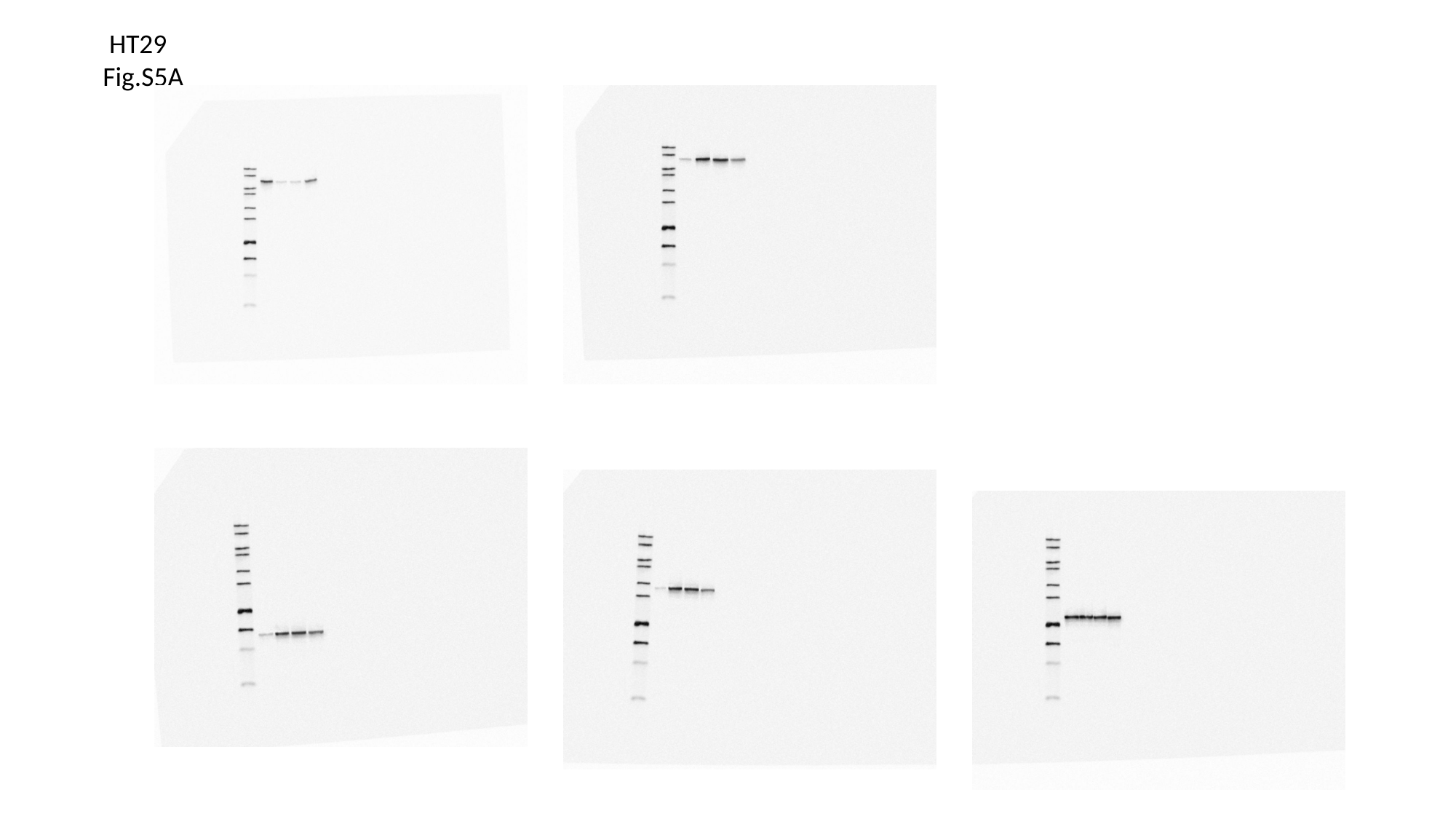

HT29
Fig.S5A

## Slide 16
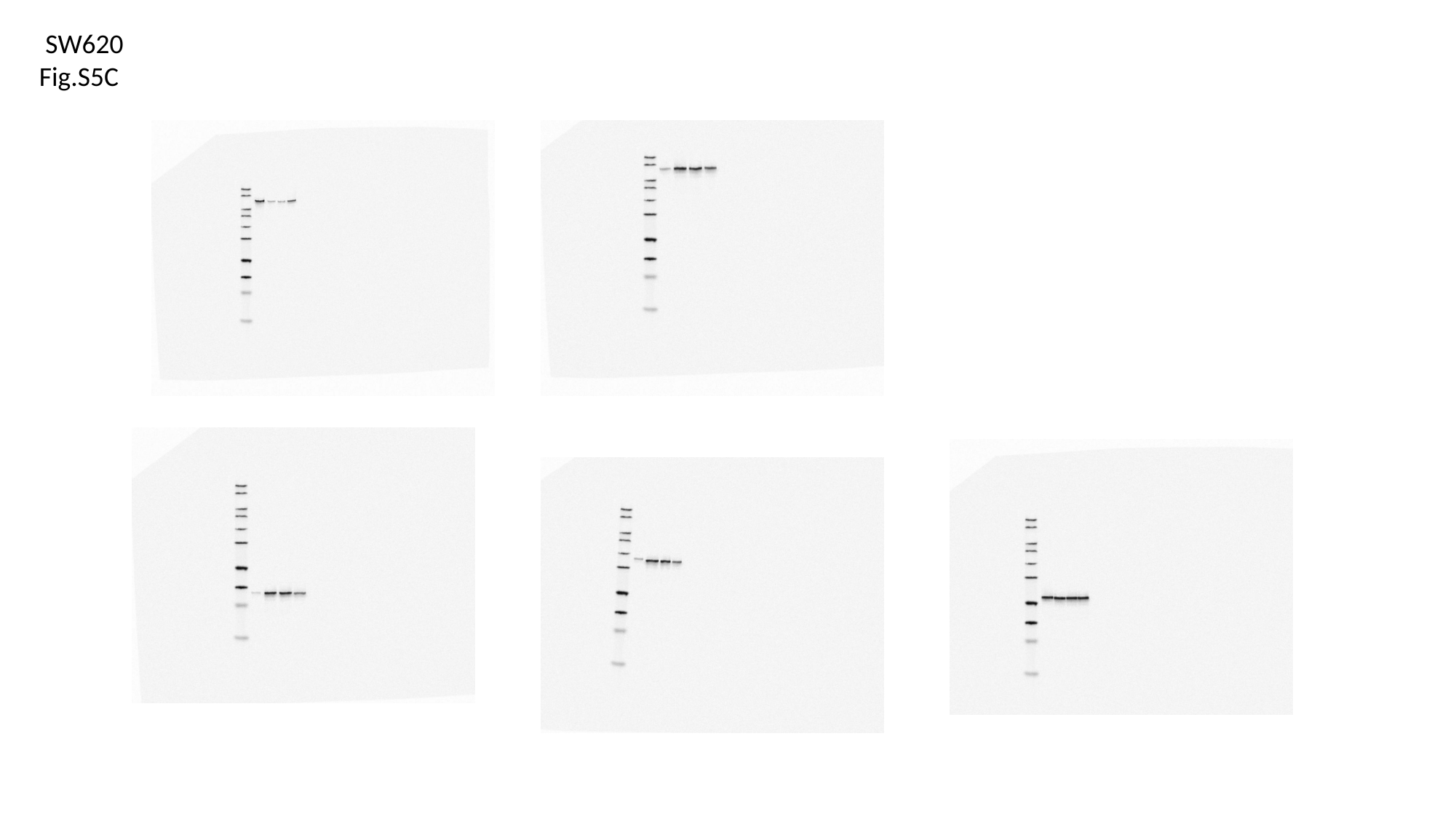

SW620
Fig.S5C

## Slide 17
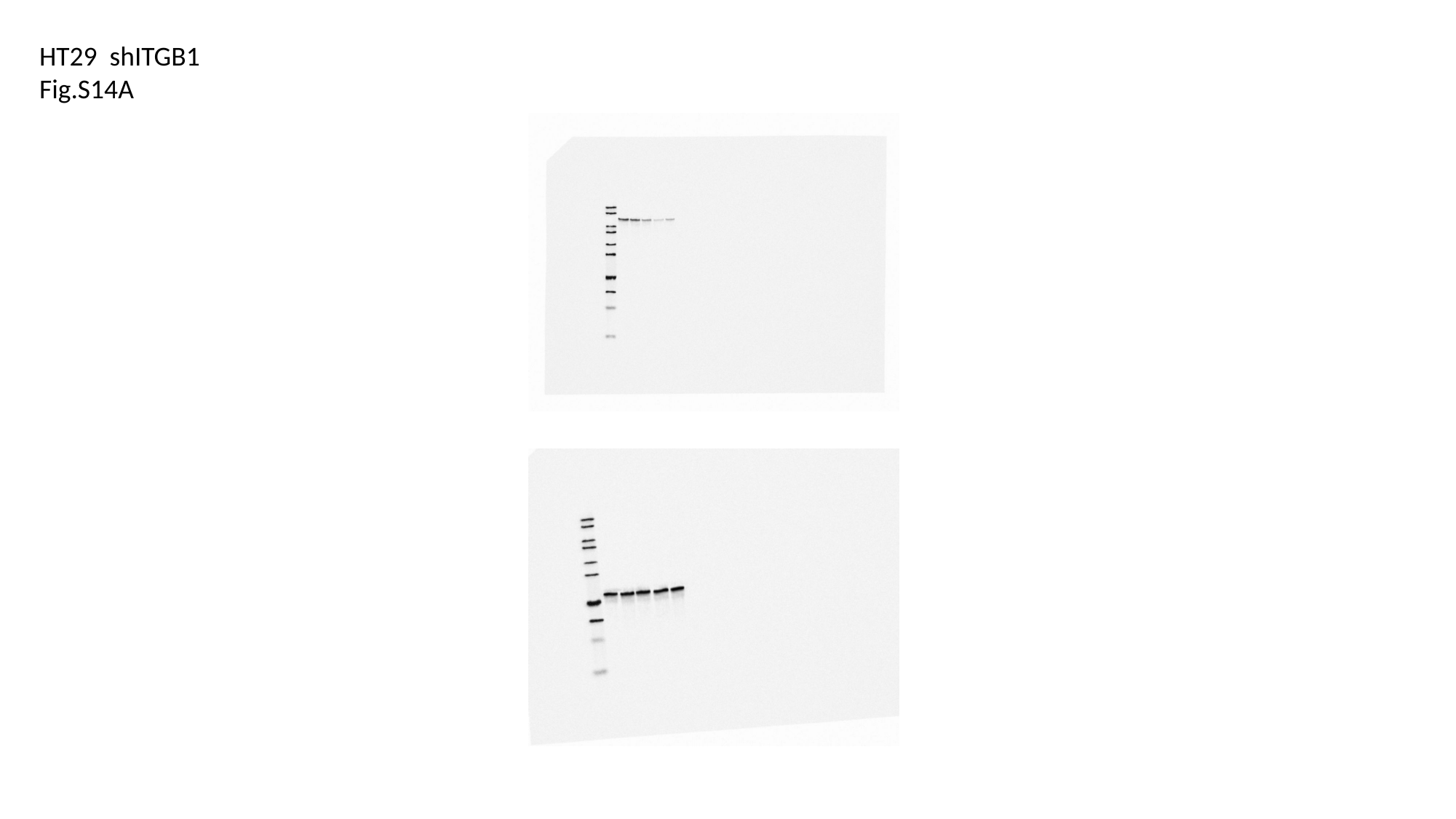

HT29 shITGB1
Fig.S14A

## Slide 18
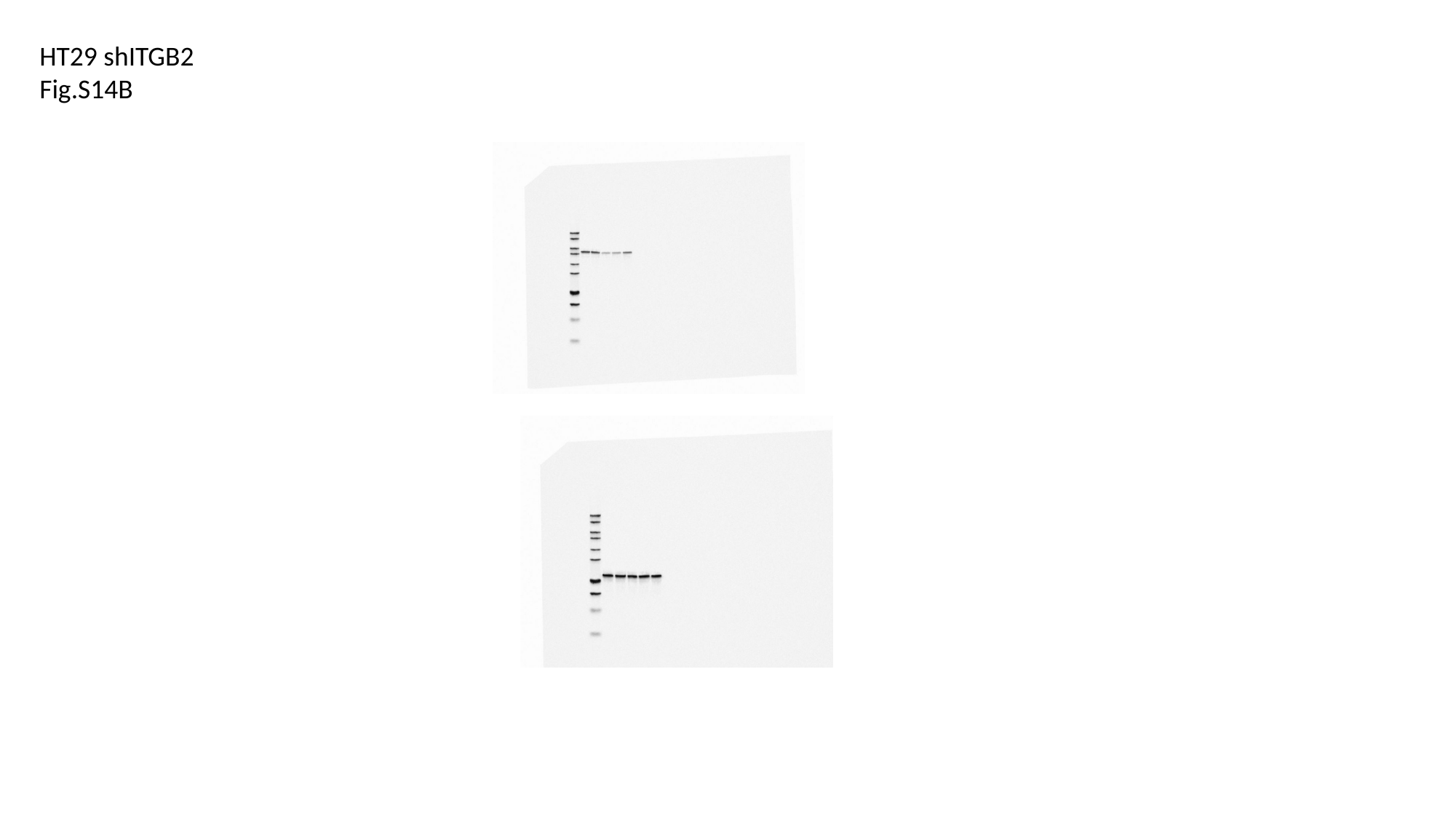

HT29 shITGB2
Fig.S14B

## Slide 19
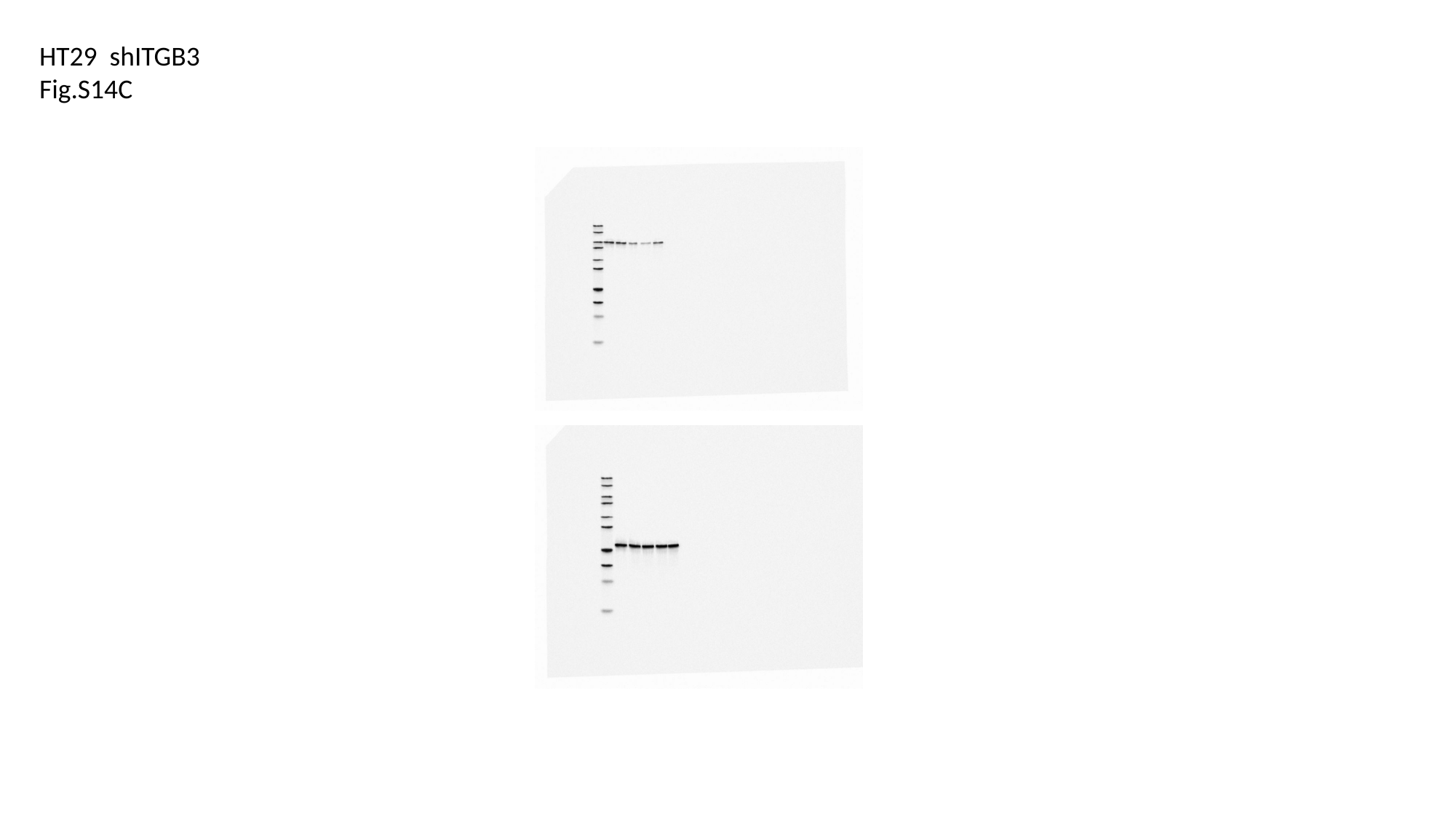

HT29 shITGB3
Fig.S14C

## Slide 20
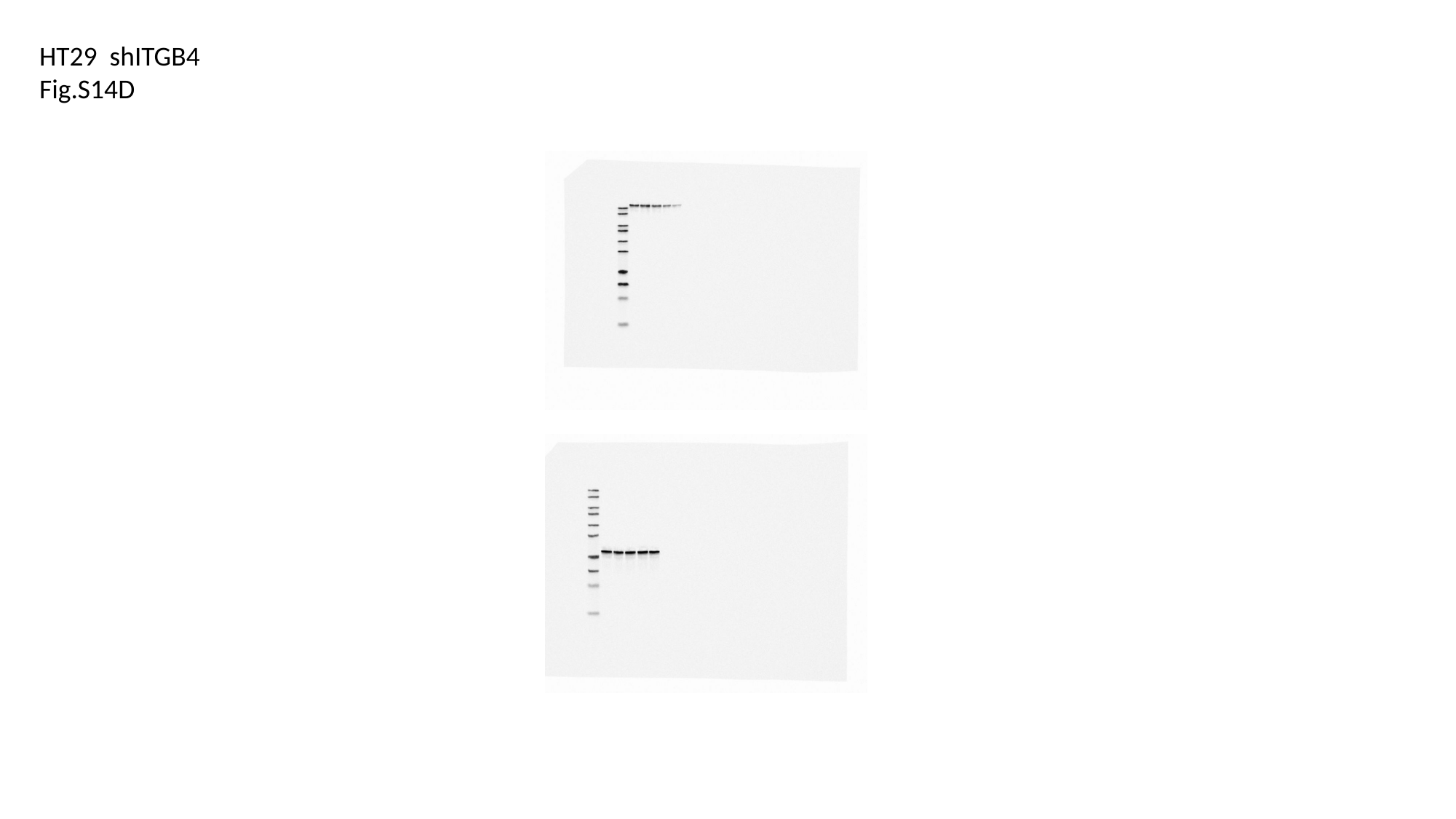

HT29 shITGB4
Fig.S14D

## Slide 21
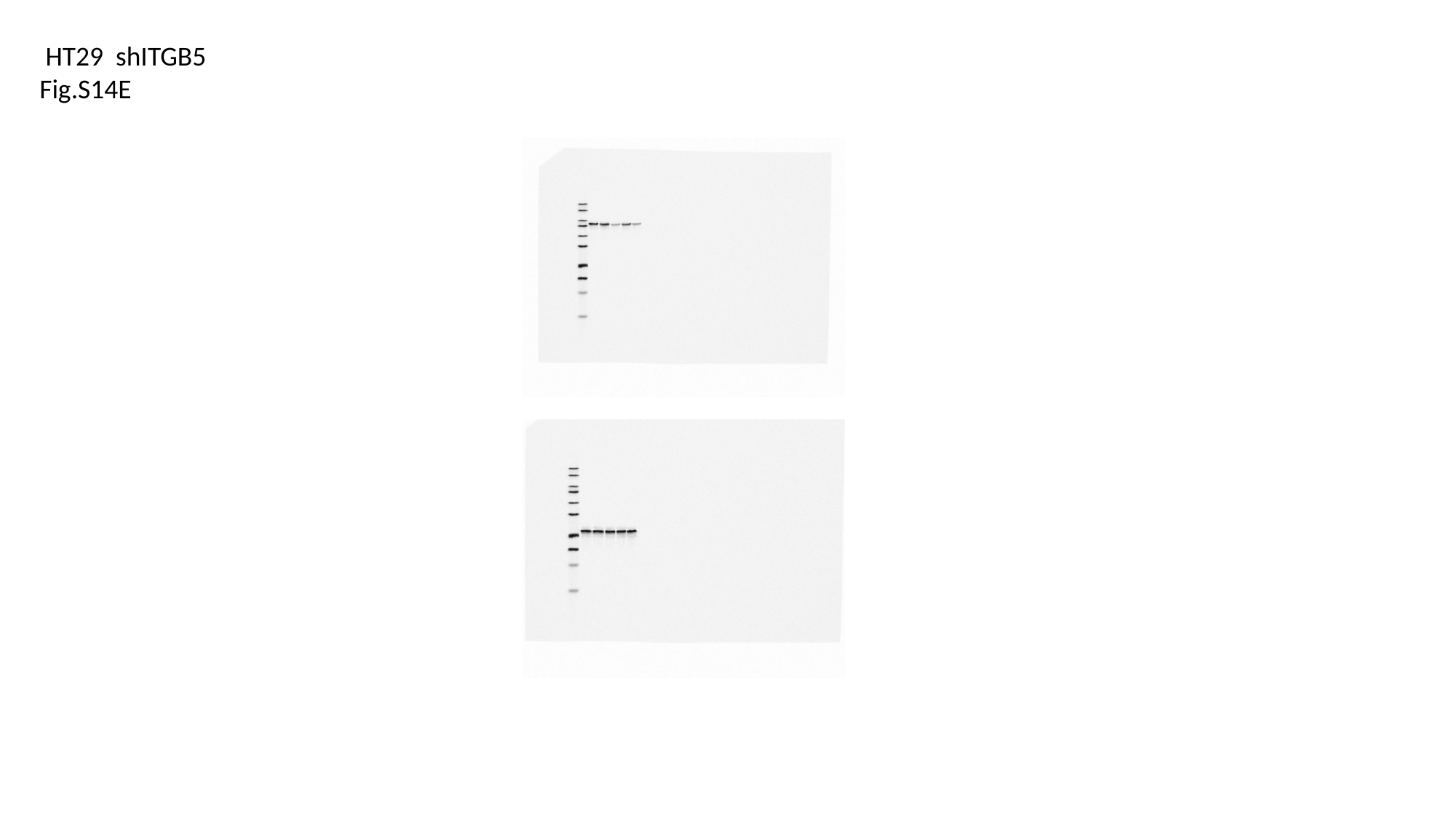

HT29 shITGB5
Fig.S14E

## Slide 22
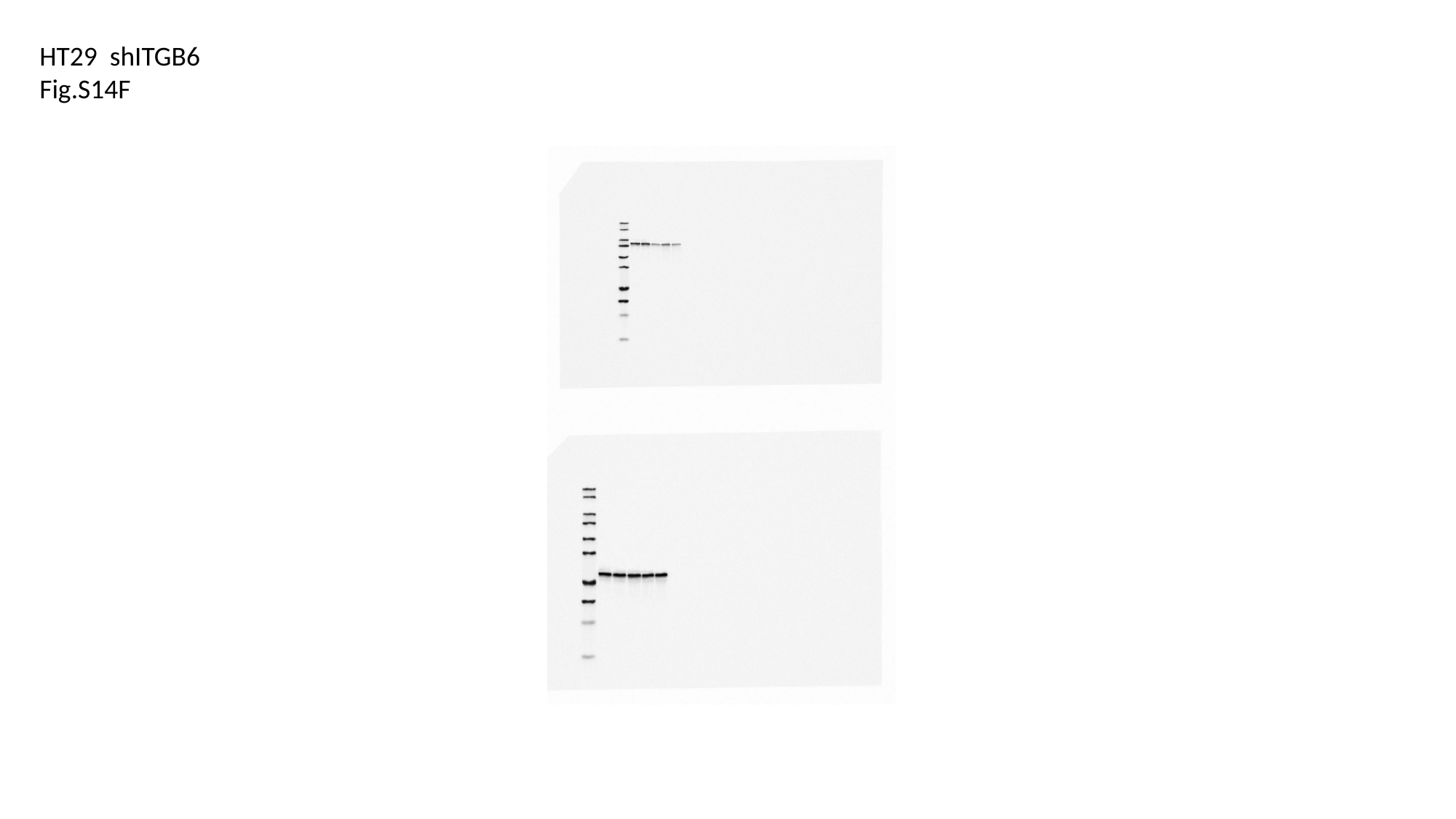

HT29 shITGB6
Fig.S14F

## Slide 23
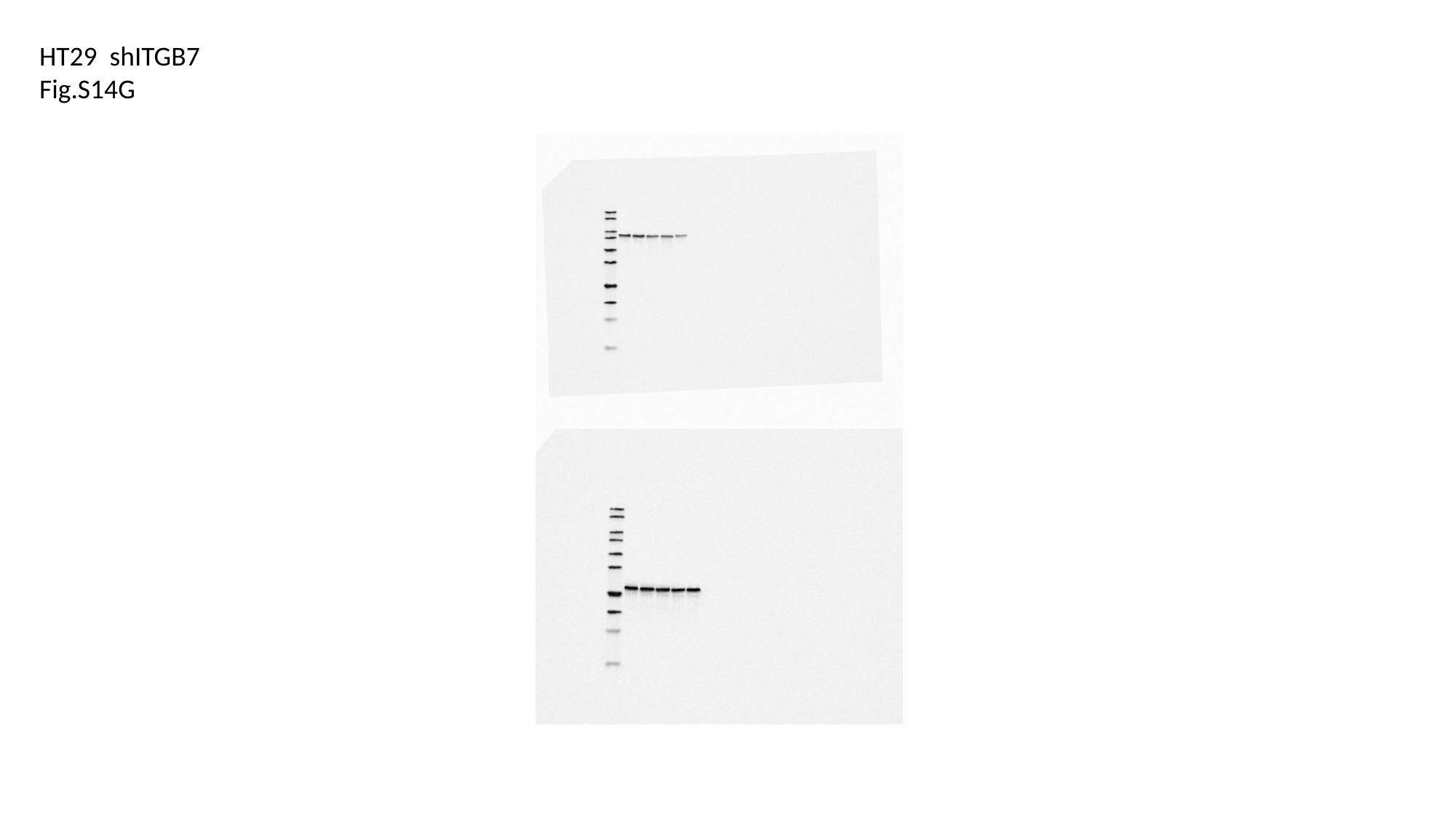

HT29 shITGB7
Fig.S14G

## Slide 24
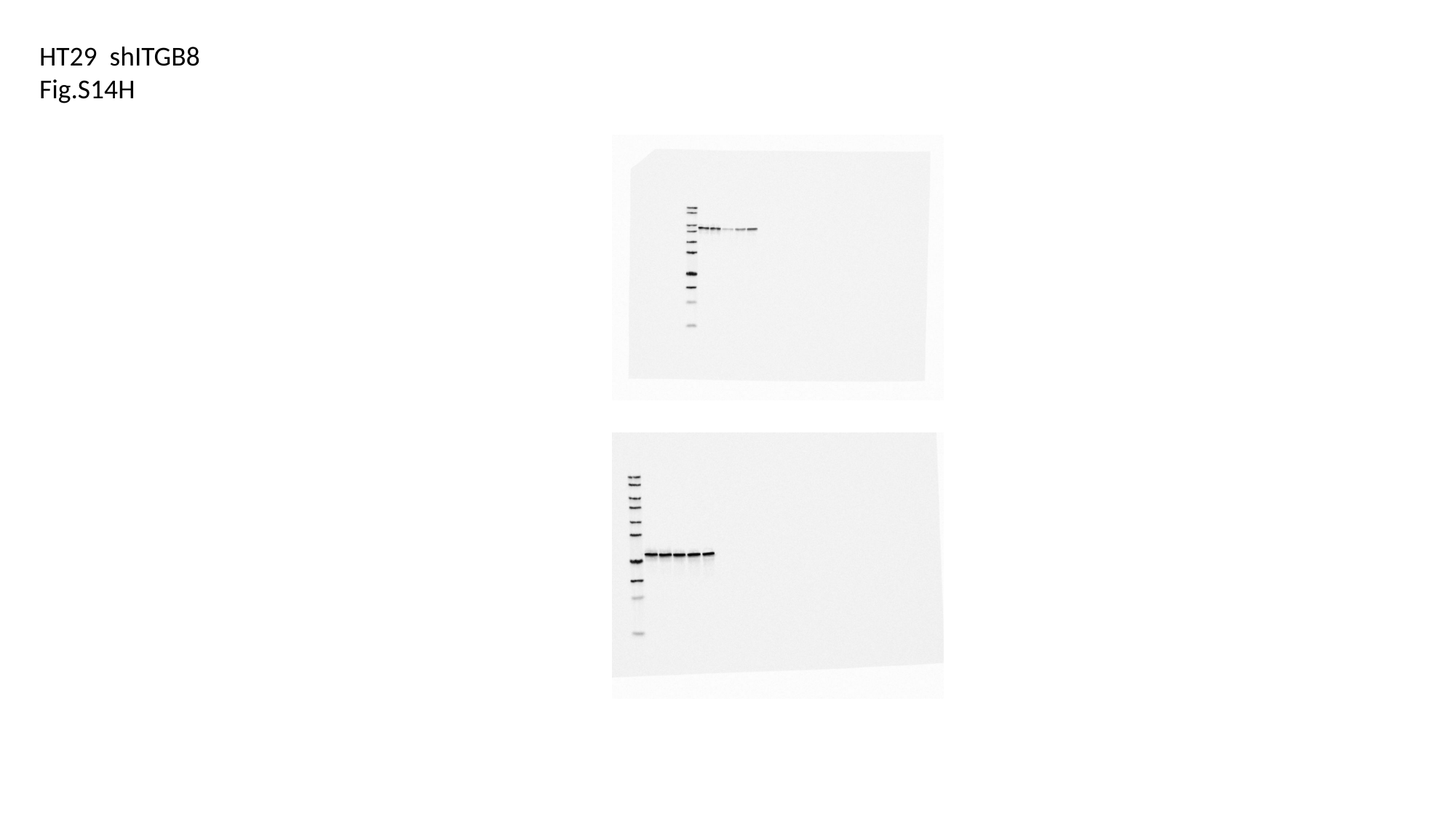

HT29 shITGB8
Fig.S14H

## Slide 25
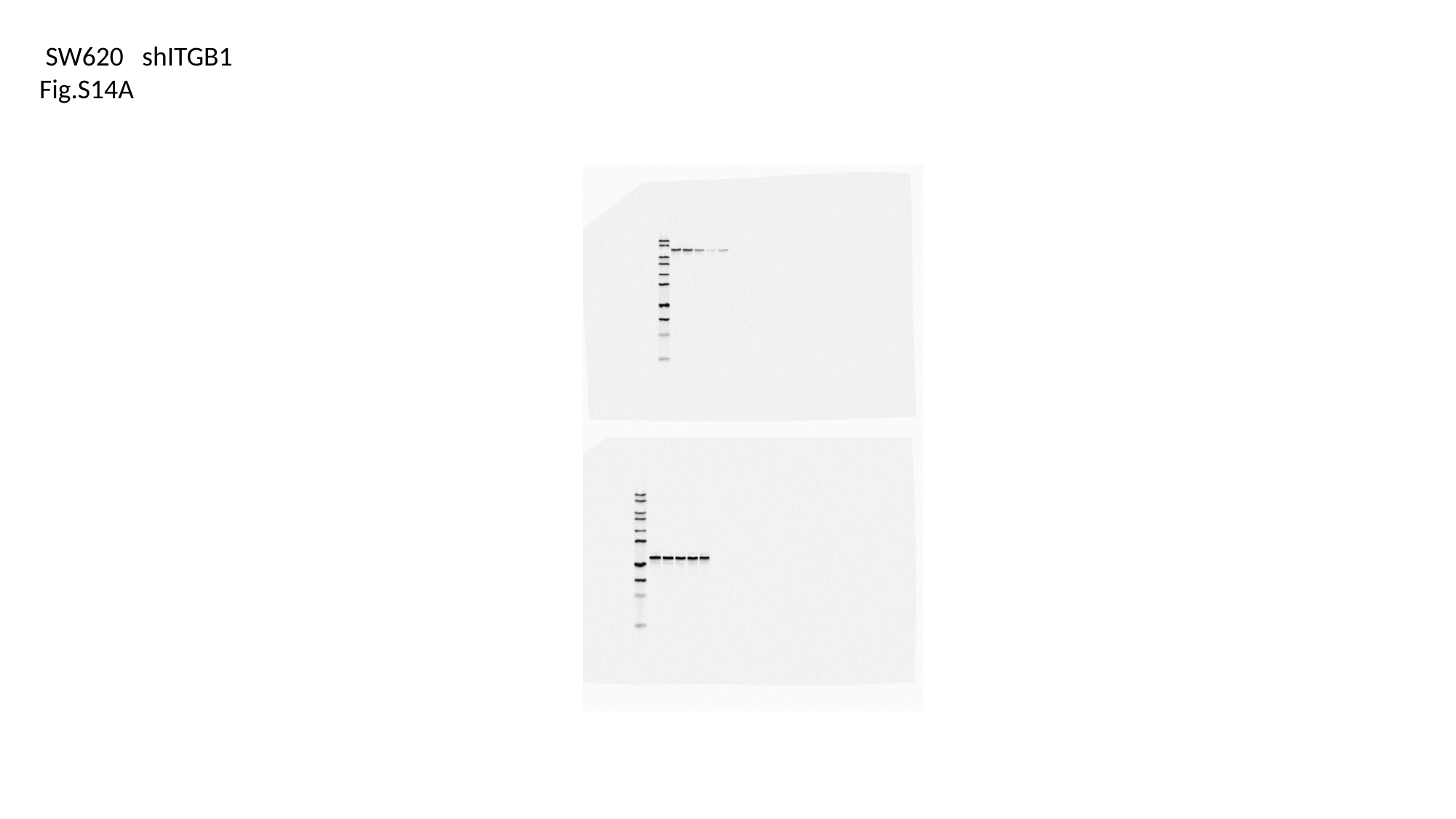

SW620 shITGB1
Fig.S14A

## Slide 26
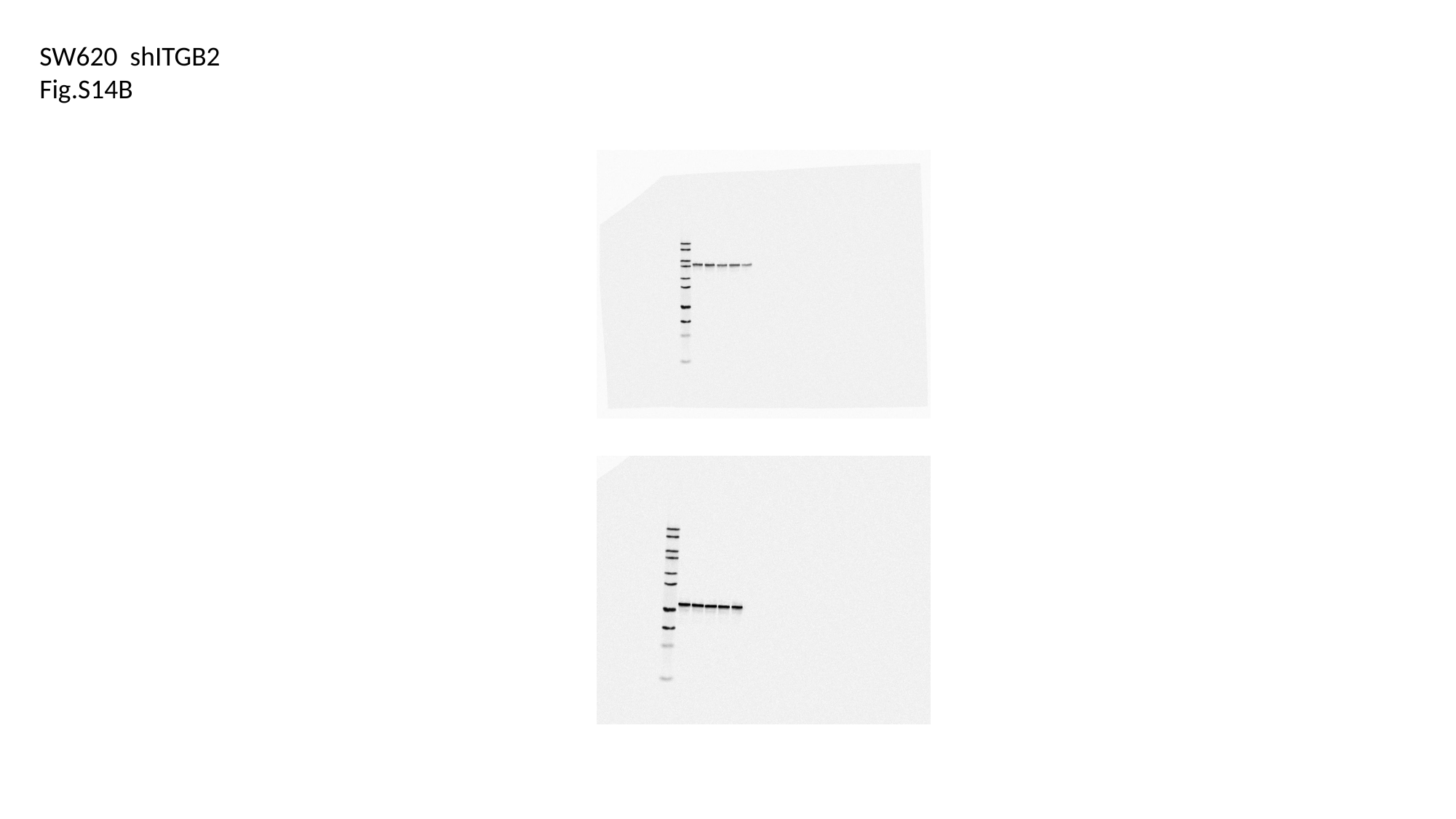

SW620 shITGB2
Fig.S14B

## Slide 27
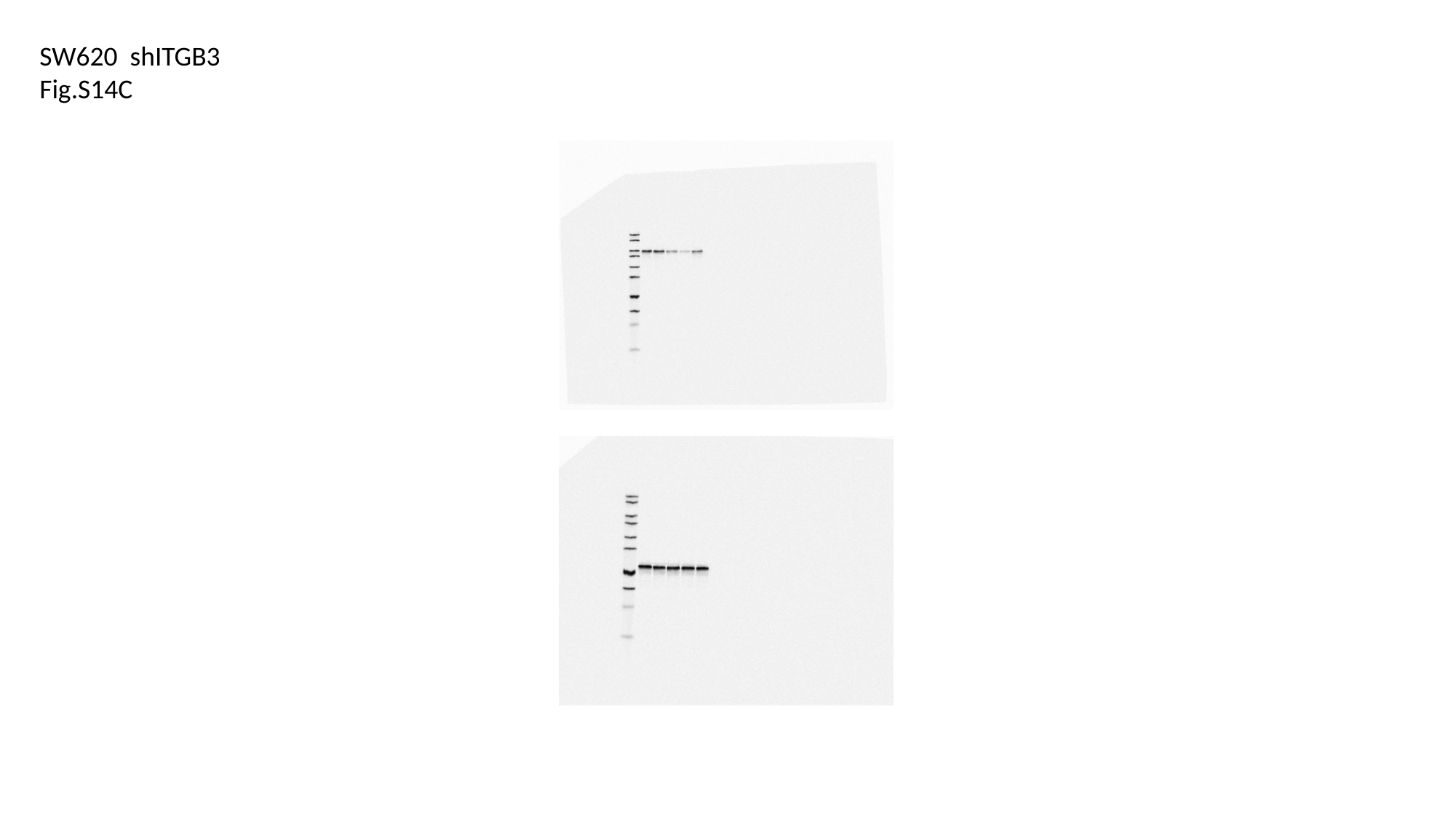

SW620 shITGB3
Fig.S14C

## Slide 28
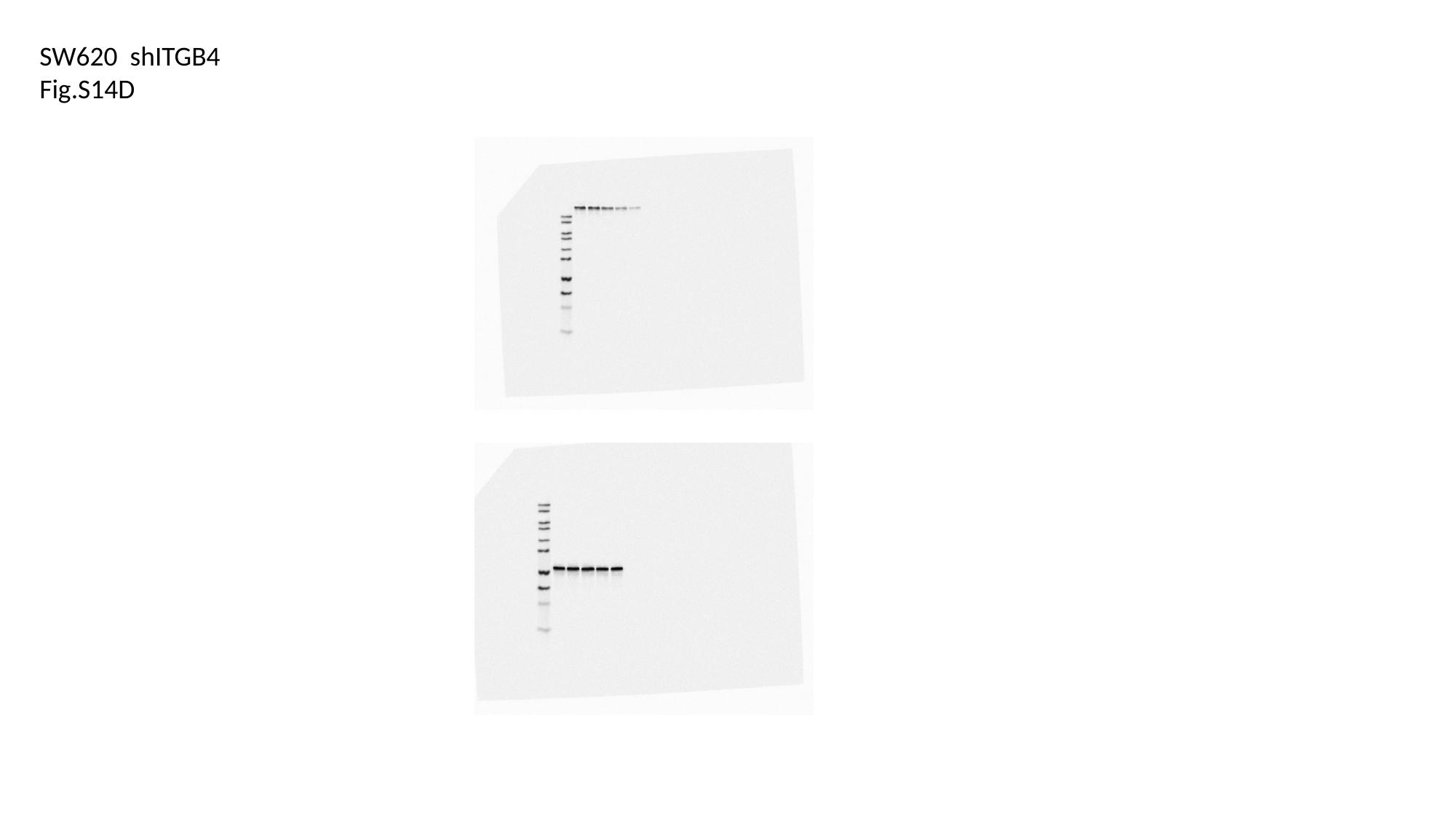

SW620 shITGB4
Fig.S14D

## Slide 29
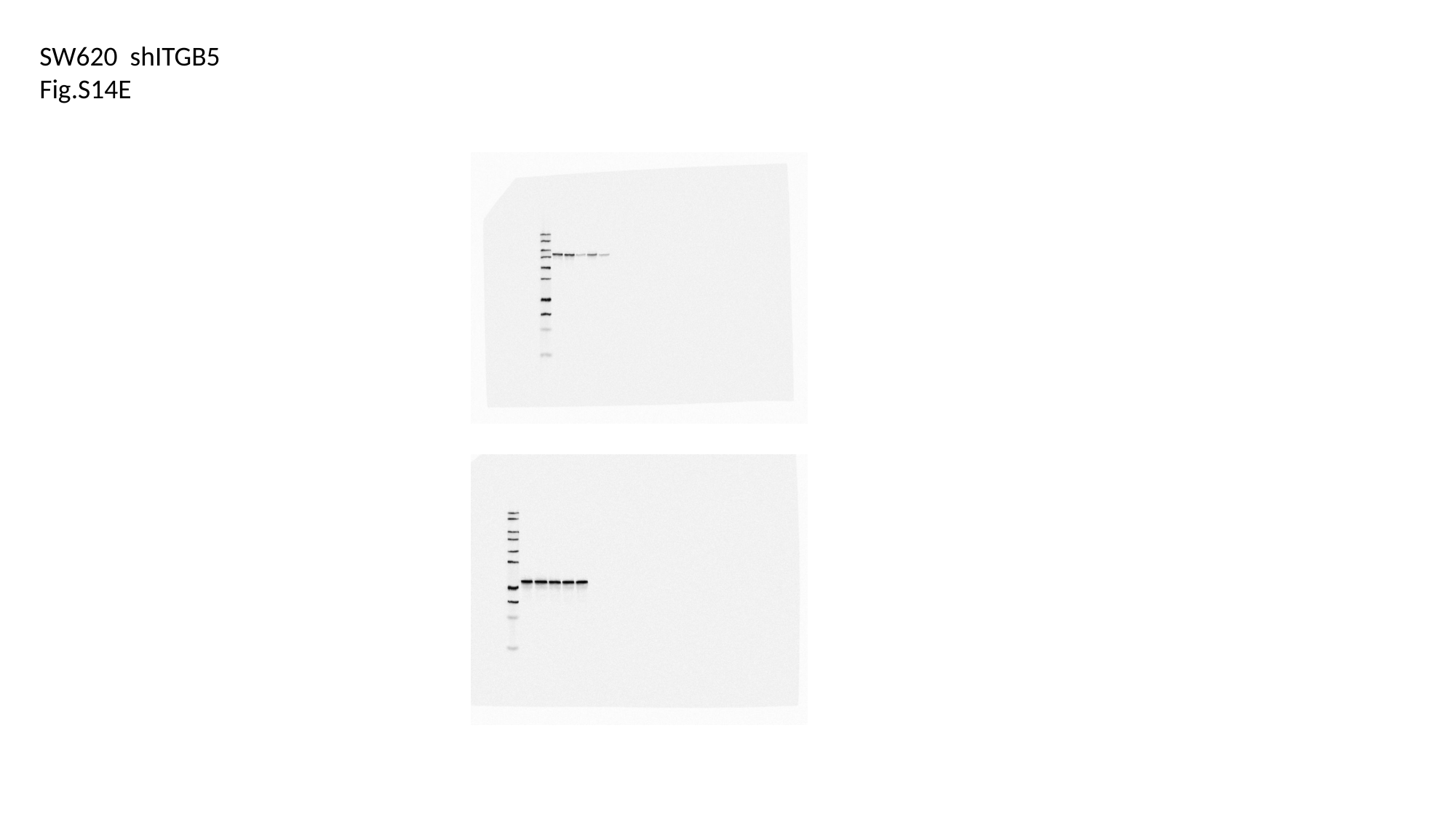

SW620 shITGB5
Fig.S14E

## Slide 30
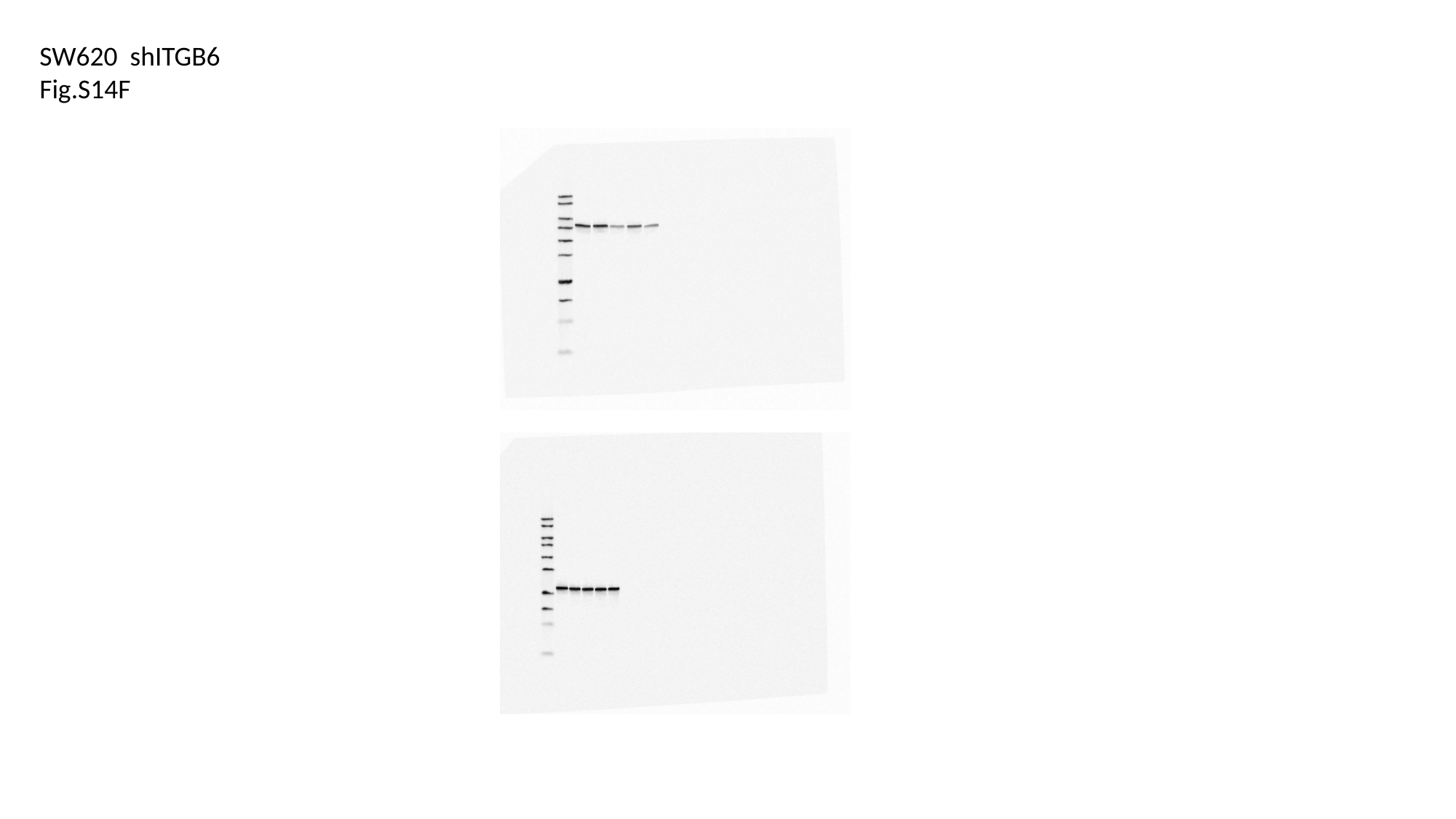

SW620 shITGB6
Fig.S14F

## Slide 31
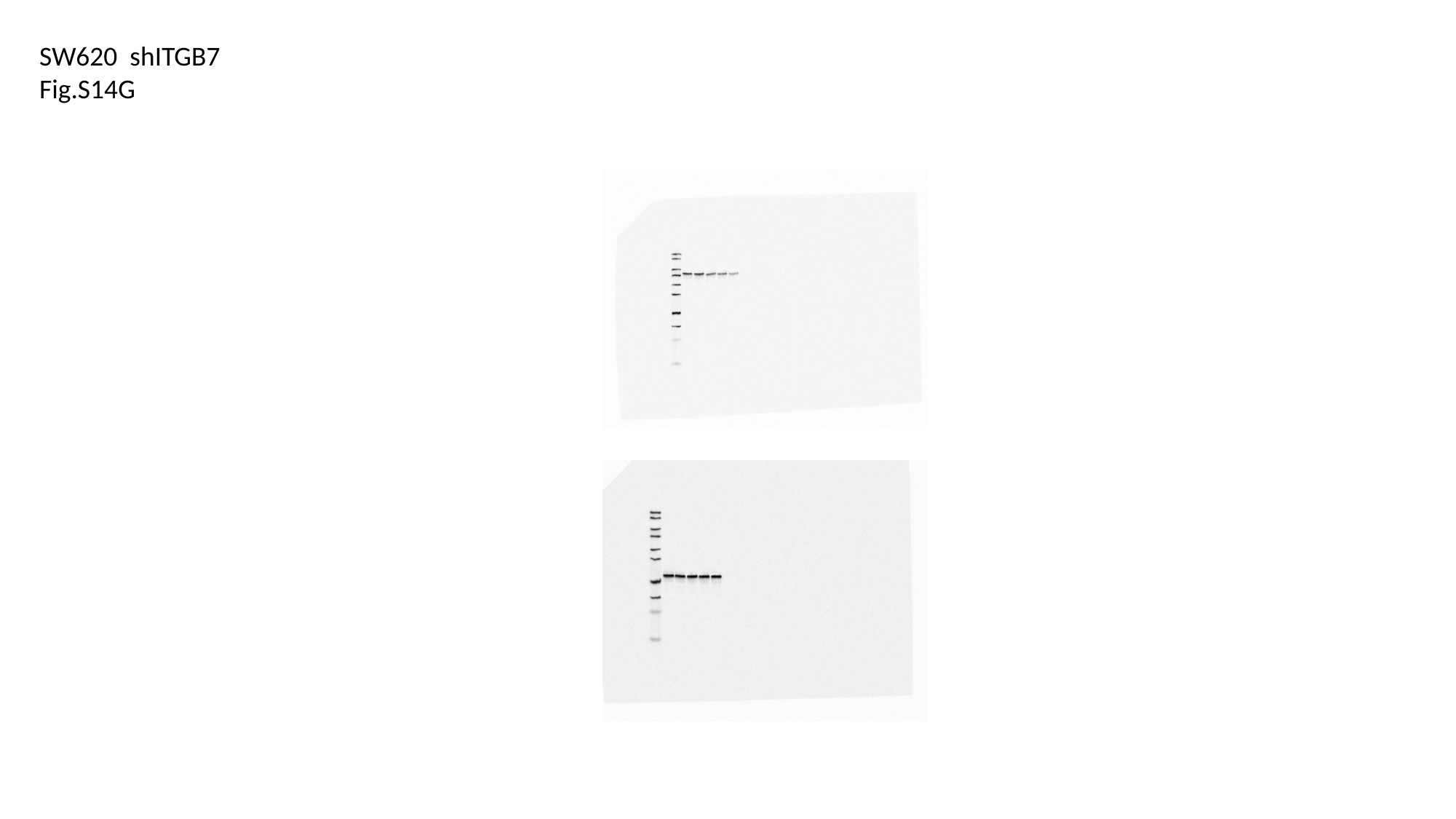

SW620 shITGB7
Fig.S14G

## Slide 32
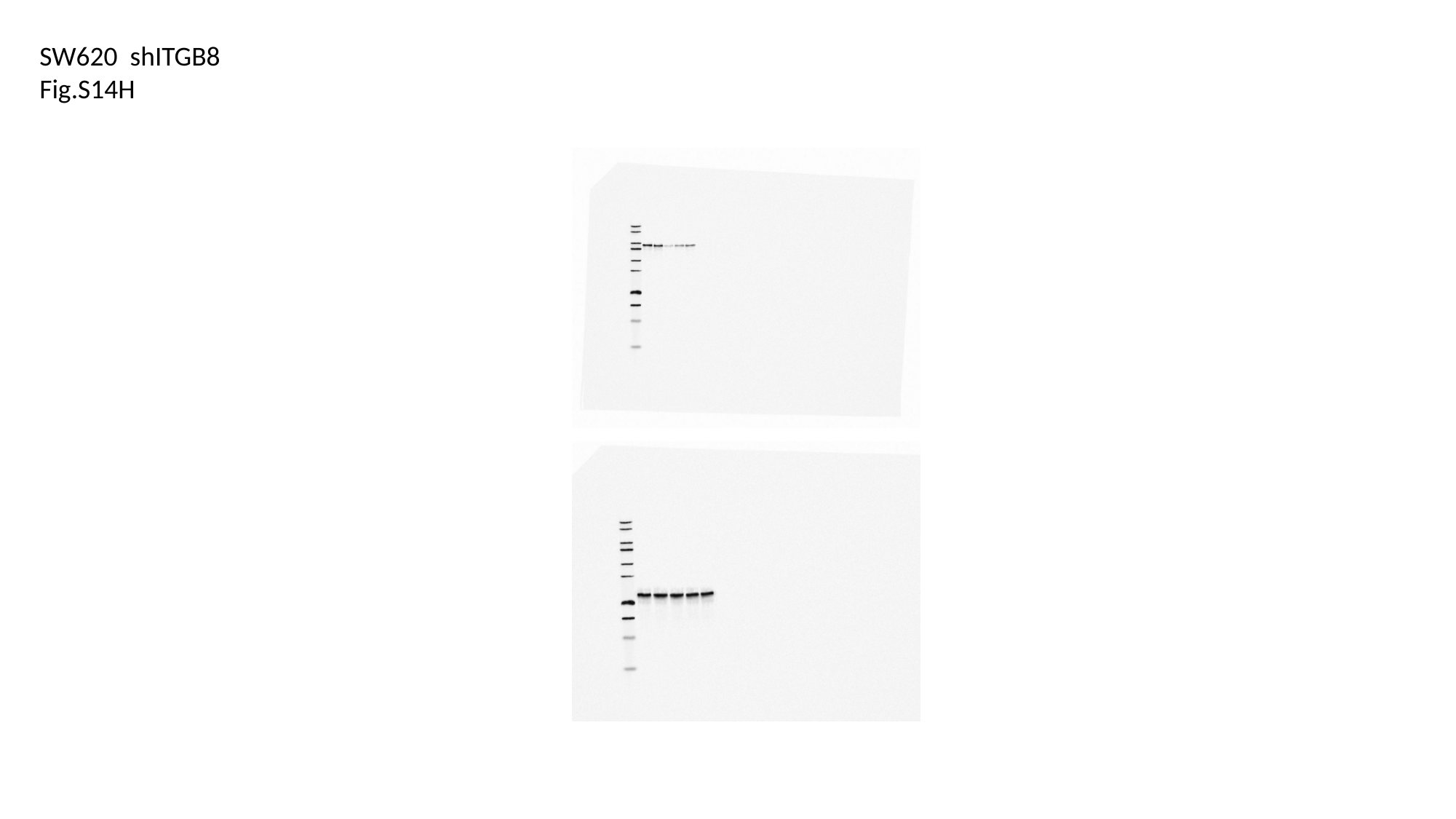

SW620 shITGB8
Fig.S14H

## Slide 33
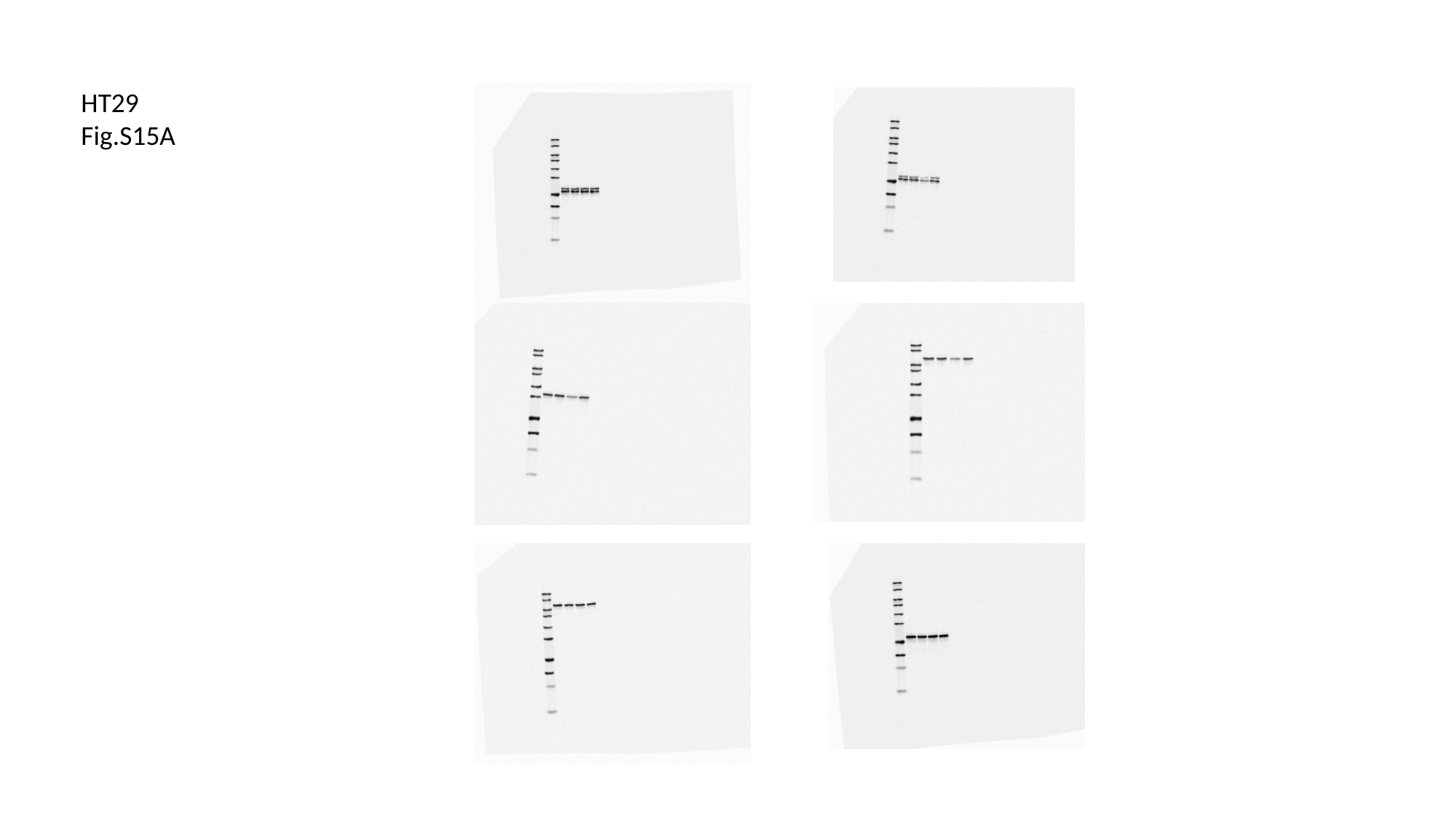

HT29
Fig.S15A

## Slide 34
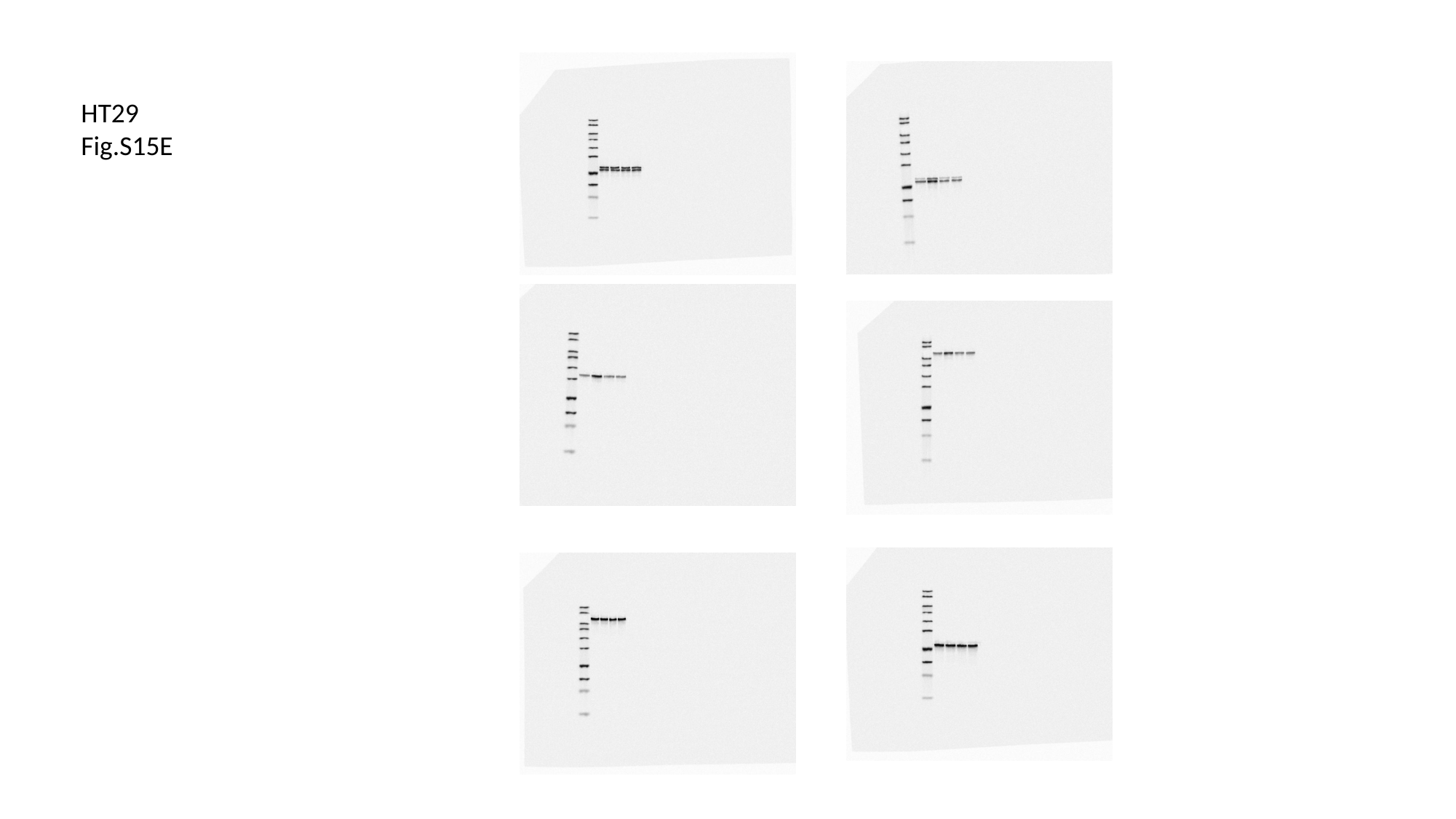

HT29
Fig.S15E

## Slide 35
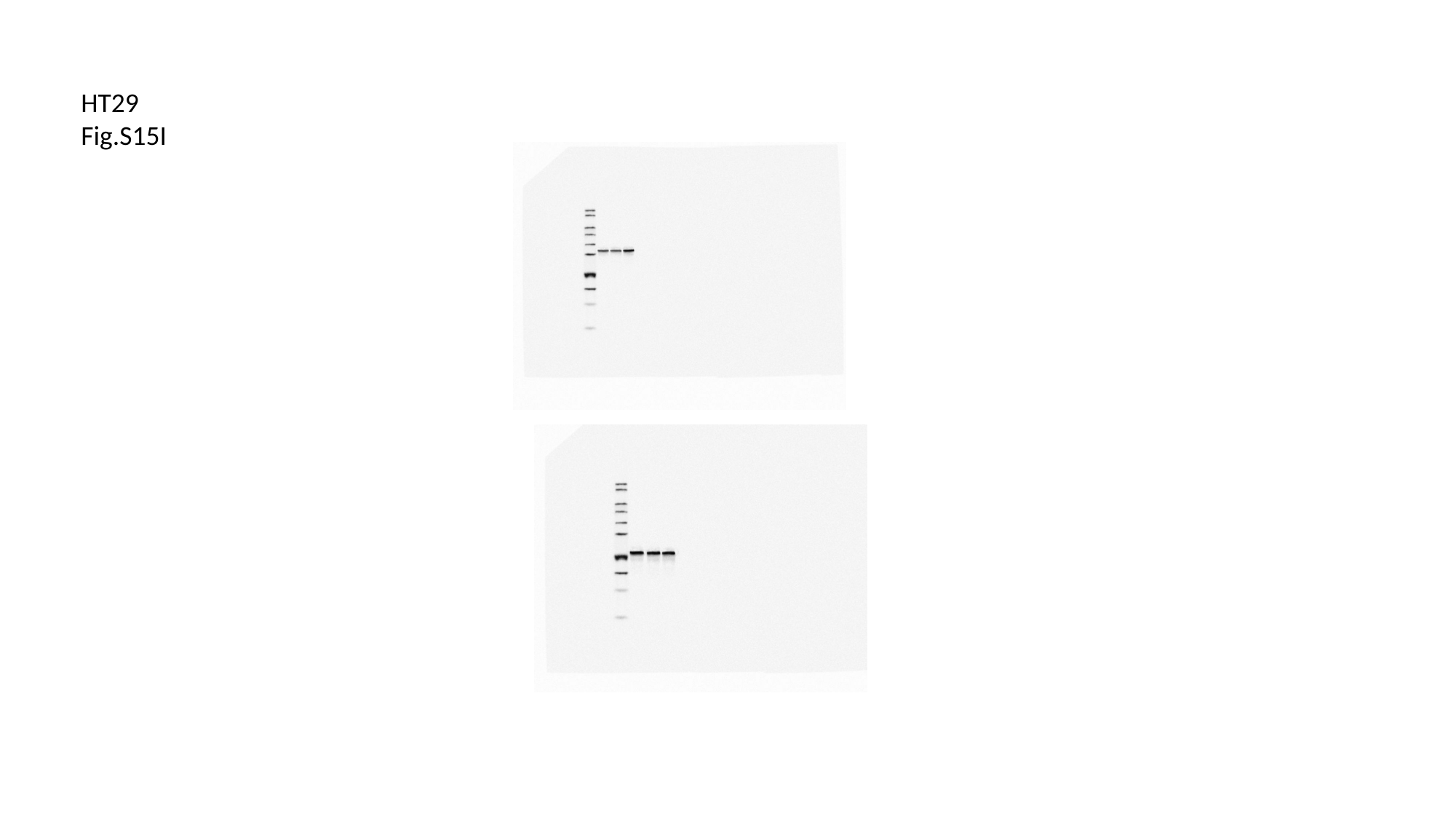

HT29
Fig.S15I

## Slide 36
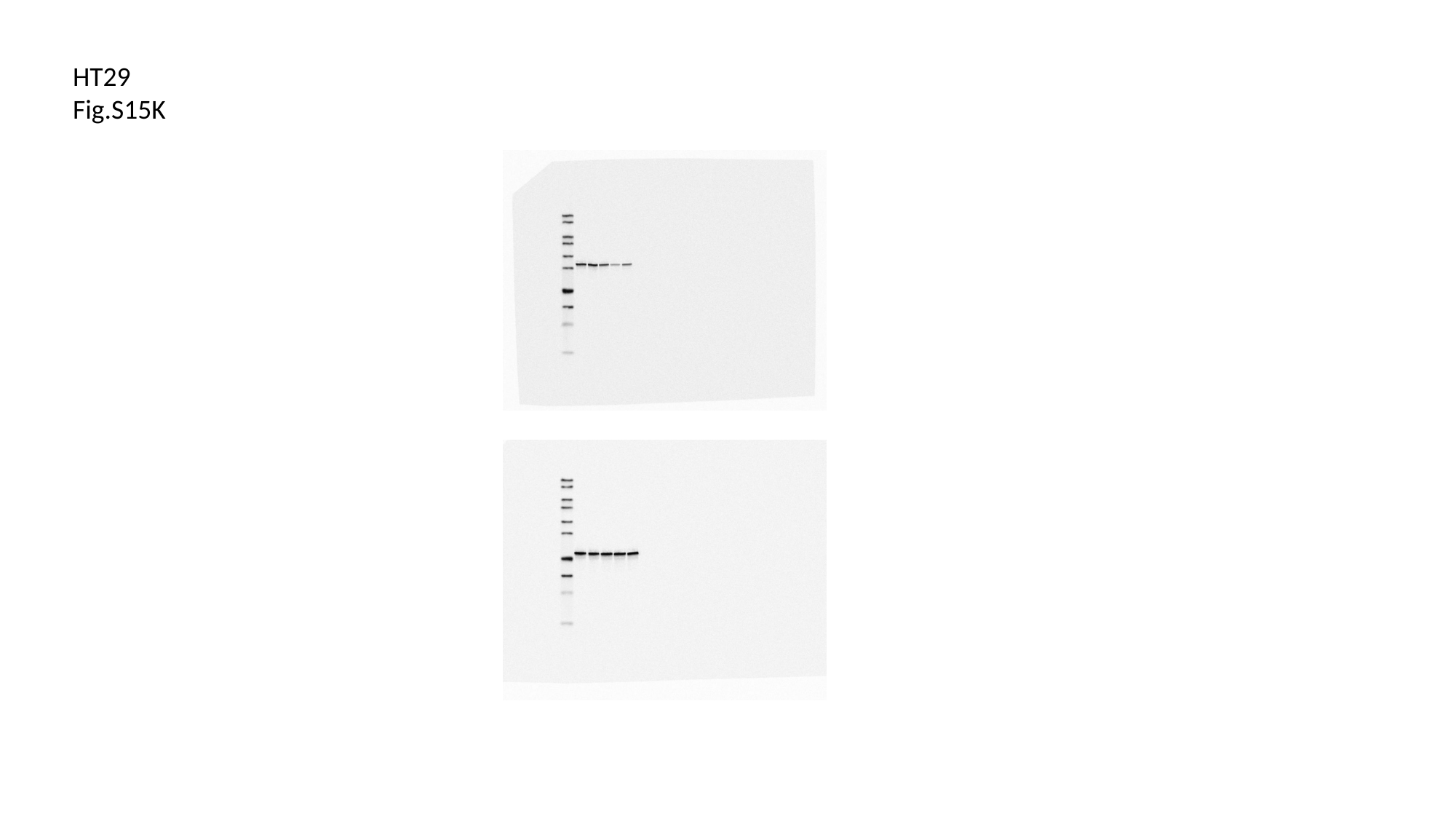

HT29
Fig.S15K

## Slide 37
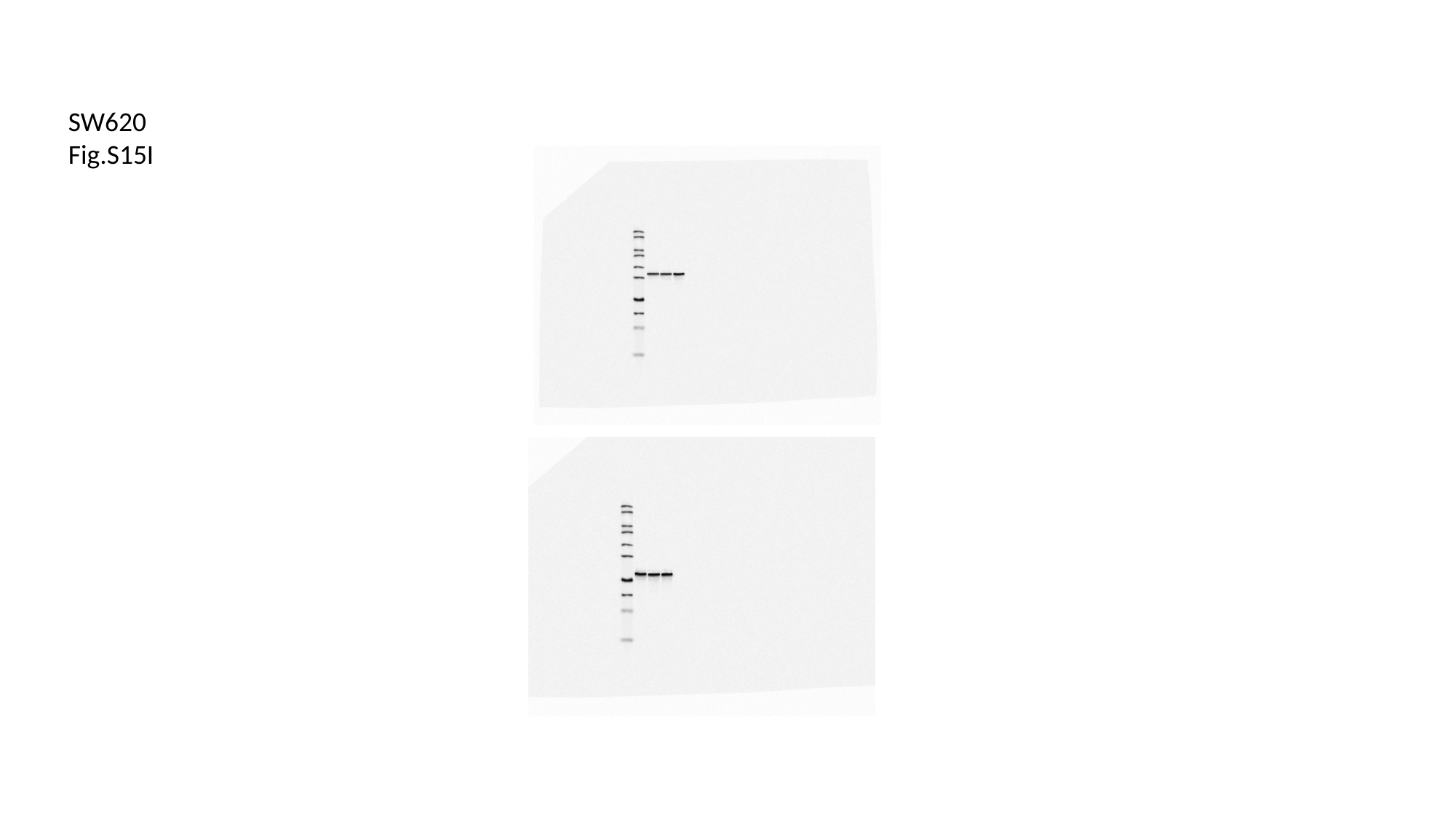

SW620
Fig.S15I

## Slide 38
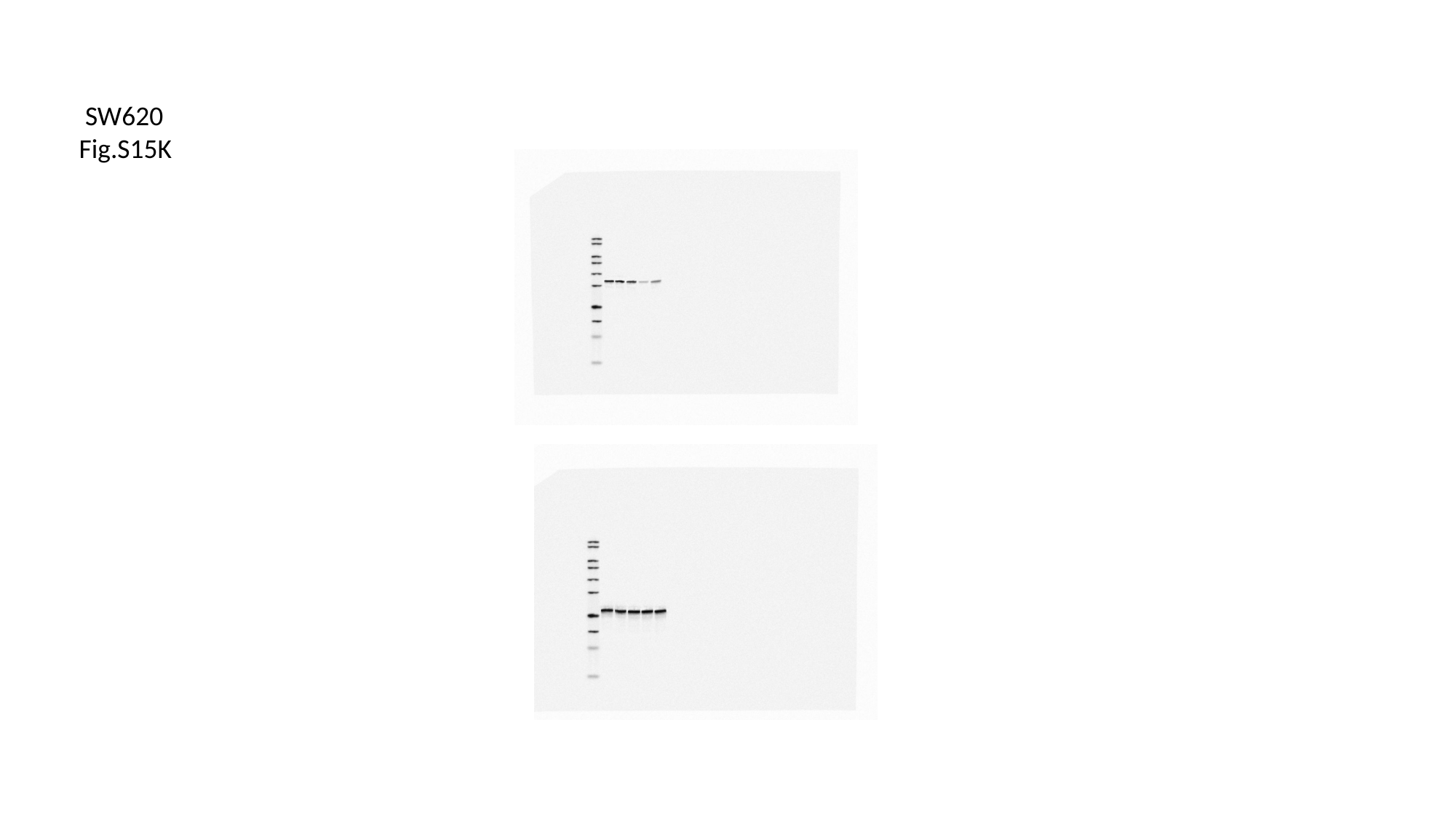

SW620
Fig.S15K

## Slide 39
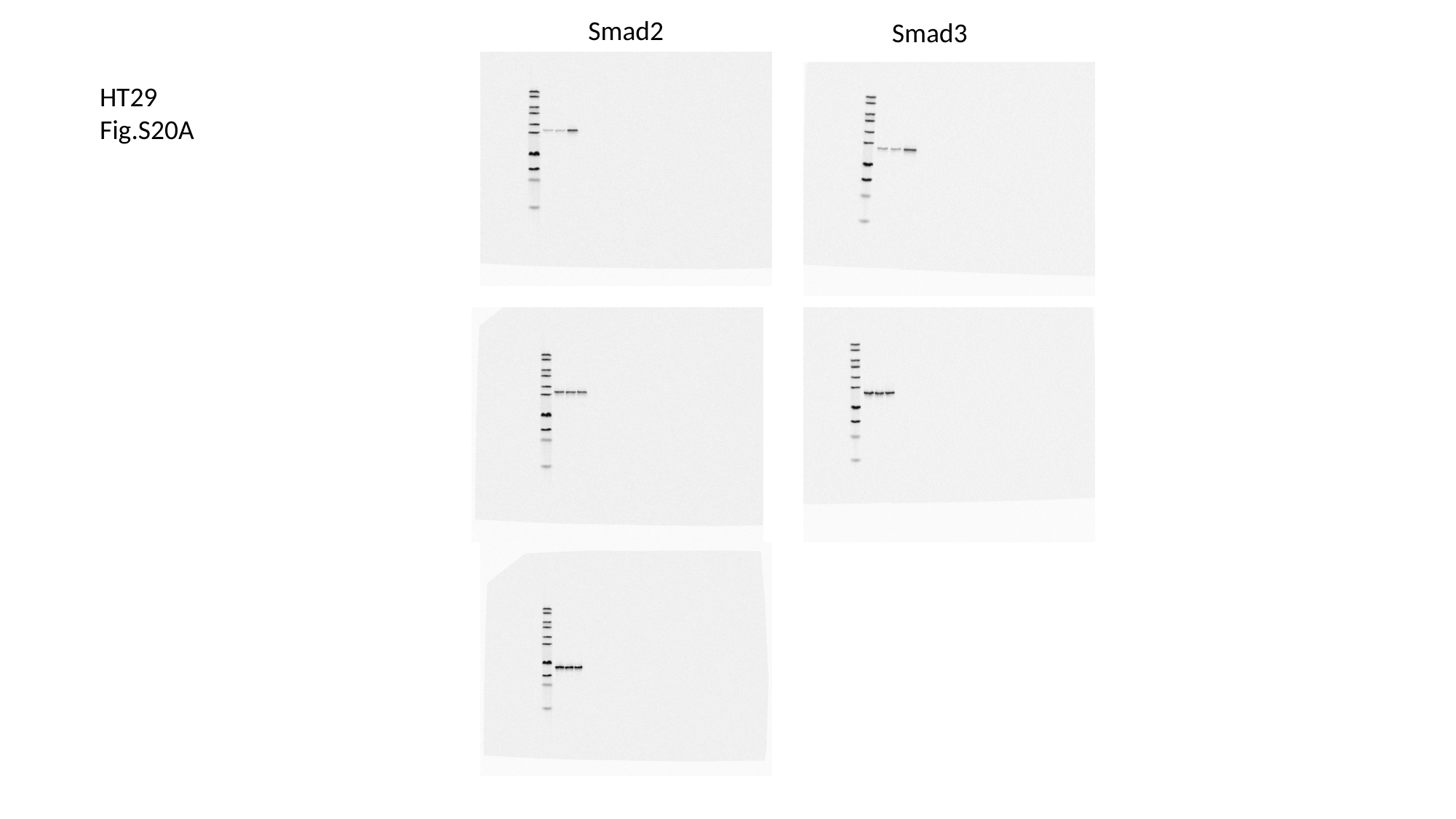

Smad2
Smad3
HT29
Fig.S20A

## Slide 40
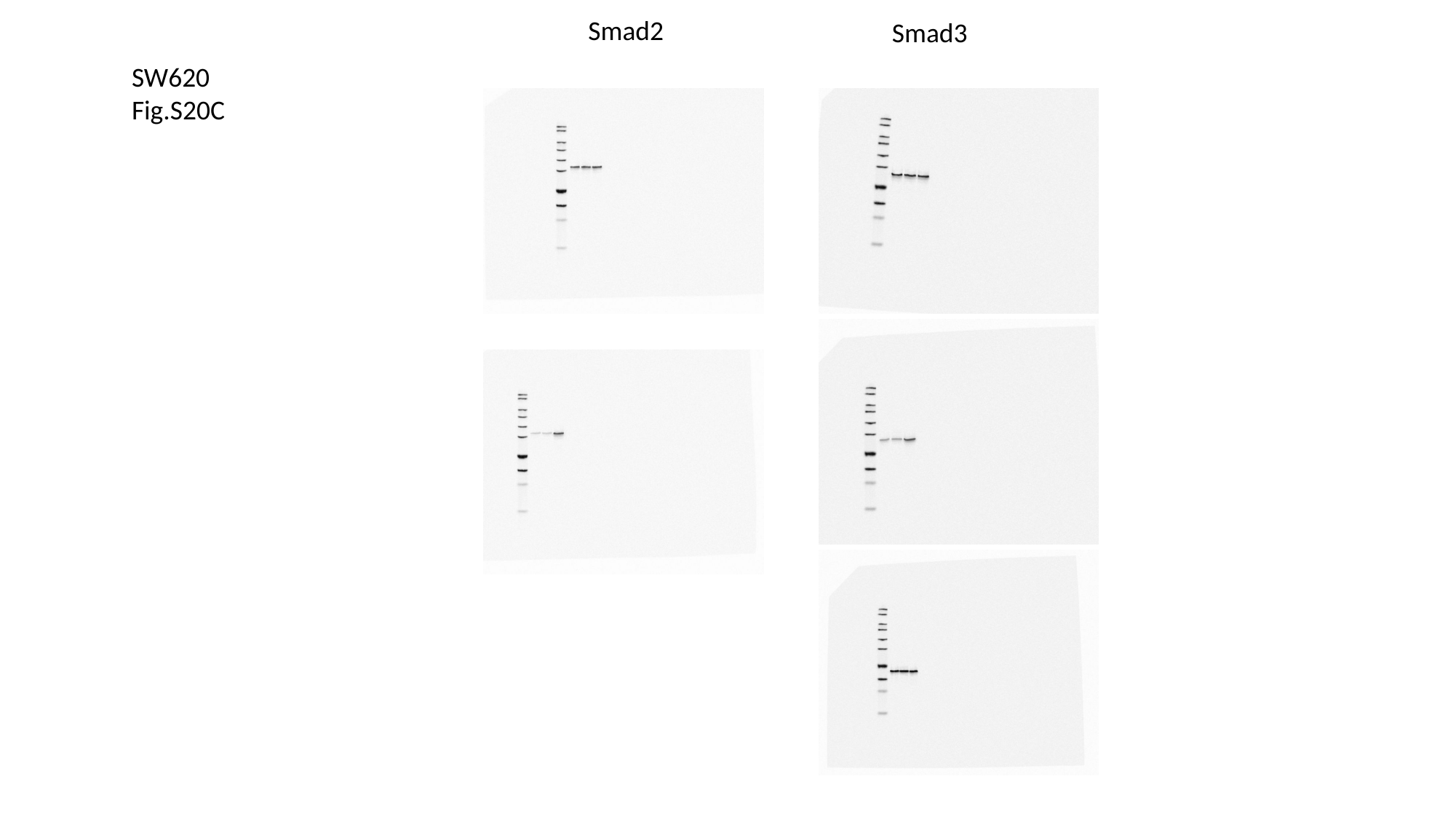

Smad2
Smad3
SW620
Fig.S20C
